# *In silico* studies of anti-oxidative and hot temperament-based phytochemicals as natural inhibitors of SARS-CoV-2 M^pro^

**DOI:** 10.1371/journal.pone.0295014

**Published:** 2023-11-30

**Authors:** Ramin Naderi Beni, Parisa Elyasi-Ebli, Sajjad Gharaghani, Arefeh Seyedarabi

**Affiliations:** 1 Department of Biochemistry, Institute of Biochemistry and Biophysics, University of Tehran, Tehran, Iran; 2 Laboratory of Bioinformatics and Drug Design, Institute of Biochemistry and Biophysics, University of Tehran, Tehran, Iran; HUE: Horus University, EGYPT

## Abstract

Main protease (M^pro^) of SARS-CoV-2 is considered one of the key targets due to its role in viral replication. The use of traditional phytochemicals is an important part of complementary/alternative medicine, which also accompany the concept of temperament, where it has been shown that hot medicines cure cold and cold medicines cure hot, with cold and hot pattern being associated with oxidative and anti-oxidative properties in medicine, respectively. Molecular docking in this study has demonstrated that a number of anti-oxidative and hot temperament-based phytochemicals have high binding affinities to SARS-CoV-2 M^pro^, both in the monomeric and dimeric deposited states of the protein. The highest ranking phytochemicals identified in this study included savinin, betulinic acid and curcumin. Complexes of savinin, betulinic acid, curcumin as well as Nirmatrelvir (the only approved inhibitor, used for comparison) bound to SARS-CoV-2 M^pro^ were further subjected to molecular dynamics simulations. Subsequently, RMSD, RMSF, Rg, number of hydrogen bonds, binding free energies and residue contributions (using MM-PBSA) and buried surface area (BSA), were analysed. The computational results suggested high binding affinities of savinin, betulinic acid and curcumin to both the monomeric and dimeric deposited states of M^pro^, while highlighting the lower binding energy of betulinic acid in comparison with savinin and curcumin and even Nirmatrelvir, leading to a greater stability of the betulinic acid-SARS-CoV-2 M^pro^ complex. Overall, based on the increasing mutation rate in the spike protein and the fact that the SARS-CoV-2 M^pro^ remains highly conserved, this study provides an insight into the use of phytochemicals against COVID-19 and other coronavirus diseases.

## Introduction

As stated by the World Health Organization (WHO), viral diseases display serious threats to the global health. There have been numerous viral pandemics over the past two decades. One example has been the Severe Acute Respiratory Syndrome Coronavirus (SARS-CoV) in 2002 to 2003 in China and Hong Kong. Later in 2009, the world was challenged by the H1N1 influenza and afterwards in 2012, a new respiratory syndrome coronavirus, the Middle East Respiratory Syndrome Coronavirus (MERS-CoV), appeared in the middle east, Saudi Arabia [1]. In 2019, a new respiratory syndrome coronavirus, called the 2019 novel coronavirus, appeared initially in China and then spread all over the world. The International Committee on Taxonomy of Viruses (ICTV) named the virus Severe Acute Respiratory Syndrome Coronavirus 2 (SARS-CoV-2) and the WHO officially announced a new name for the disease; coronavirus disease 2019 (COVID-19) [2,3]. COVID-19 has since become a global pandemic and up until mid November 2023, over 697,000,000 confirmed infected cases, and over 6,900,000 million deaths have been reported, with over 669,000,000 recovered cases [4]. The number of confirmed cases and deaths reported are still on the rise.

SARS-CoV-2 is an enveloped, positive-sense, single-stranded RNA virus and the length of the genome is around 30 Kb. The replication of SARS-CoV-2 depends on polyproteins pp1a and pp1ab, which are encoded by about two-thirds of the 5’ end of the genome. These polyproteins are later processed into 16 non-structural proteins (NSPs), by two viral proteases, the main protease (M^pro^, also called the 3C like protease, 3CLpro) and the papain like protease (PLpro) [5–7]. The M^pro^, therefore, is one the proteins that has the potential of being a key target for antiviral drug development, since it is essential in the infection process and viral replication of SARS-CoV-2 [8,9].

SARS-CoV-2 M^pro^ consists of a dimer. Each monomer has three domains, with domains I & II consisting of residues 8−101 and 102−184, respectively, forming an antiparallel β-barrel structure, while domain III, consists of residues 201−303, mainly composed of α-helices. SARS-CoV-2 M^pro^ includes a Cys145-His41 catalytic dyad, and a cleft between domains I and II, where the inhibitor/substrate binding site is located. Each N-terminus residue, or N-finger, that is stuck between domains II and III of the monomer and domain II of the other monomer, plays a crucial role in forming an active site of M^pro^. In comparison with the monomer, which is principally inactive, the M^pro^ dimer is extremely active [10–12].

There has been growing evidence that natural plants/herbs have been used in the form of teas, topical ointments and dietary supplements to relieve respiratory ailments including cough and bronchospasm [13–15]. In addition, there is a growing research interest on natural products with anti-inflammatory and anti-oxidative abilities to prevent and clarify the pathogenesis of various diseases that involve chronic inflammatory responses by the host to self-protect and remove the harmful effects such as those brought about by pathogens [16]. Interestingly, a comprehensive review published in 2018 [17], reported that traditional herbal medicines are important part of complementary/alternative medicine and accompany the concept of ‘temperament’, which is of a great deal of importance not only in the body but also in disease, foods and drugs. Accordingly, it was stated that the temperament is divided into two main categories, the hot and cold. The imbalance of temperament may it be body, medicine or food, can therefore cause disease [18,19]. Even more interesting, some studies refer to cold and hot pattern in terms of oxidative and anti-oxidative properties in medicine, respectively [20]. Traditional Chinese medicine, states that hot medicines are supposed to cure cold and cold medicines are supposed to cure hot diseases [20–23]. A study in 2014 [24], revealed that food with hot (e.g. thyme) and cold (e.g. sumac) properties affect people of different temperaments. Another study showed the effect of medicine with hot or cold properties to have varying effects on patients with different temperaments [19]. Interestingly, the amount of water content decreases with the hot temperament [25]. Additionally, it has been reported that the coldness (and wetness) of the brain i.e. having a cold temperament, was associated with the severity of the COVID-19 infection and hence regarded as a factor to be considered in preventing the disease or in predicting the course of the disease [26].

The initial step taken in this study was to assess M^pro^ structural conservation using a number of known ligand-bound coronovirus structures. This was then followed by analysis of five PBD structures of SARS-CoV-2 M^pro^ itself, consisting of 6lu7, 6y2f, 6wtt, 6m0k and 6lze, with promising IC_50_ and EC_50_ values, for further emphasis on structural conservation and identification of SARS-CoV-2 M^pro^ residues involved in binding interactions with ligands/inhibitors. The reliability of molecular docking versus experimentally derived structural information was assessed, followed by evaluation of six antiviral drugs also used for COVID-19 including Remdesivir, Darunavir, Oseltamivir, Lopinavir, Ritonavir, Ribavirin and in particular Nirmatrelvir, as an approved inhibitor [27]. Subsequently, 38 compounds from plants/herbs/trees were selected in this study, with emphasis on their therapeutics properties and antiviral effects, and assessed via molecular docking. The phytochemicals with the highest binding affinities were then subjected to computational studies including molecular dynamics (MD) simulation analysis involving calculations of root mean square deviation (RMSD), root mean square fluctuation (RMSF), radius of gyration (Rg) and number of hydrogen bonds, binding free energy and residue contribution calculations by using molecular mechanics Poisson–Boltzmann surface area (MM-PBSA) and buried surface area (BSA) analysis. The study compared the monomeric (inactive) versus dimeric (active) states of the SARS-CoV-2 M^pro^ deposited structure, to assess the binding interactions of the selected phytochemicals, using Nirmatrelvir as a reference. The results highlighted the presence of certain phytochemicals, with anti-oxidantive and hot-temperament properties, as potential inhibitors of SARS-CoV-2 M^pro^.

## Results

### Bioinformatics analyses of coronavirus M^pro^ structures with bound ligands/inhibitors

In this study, initially the fasta format of the SARS-CoV-2 M^pro^ sequence was taken and a protein blast search performed in ncbi against all known coronavirus M^pro^ PDB structures, in order to assess M^pro^ structural conservation. The outcome of the search was assessed and the non-mutated, native M^pro^ structures with bound inhibitors were selected and assessed by multiple sequence alignment (S1 Fig in S1 File). As seen in S1 Table in S1 File, the sequence identity of various coronavirus M^pro^ compared to SARS-CoV-2 M^pro^ ranged from the highest at 96% for SARS-CoV to about 41% for human coronavirus.

**Fig 1:**
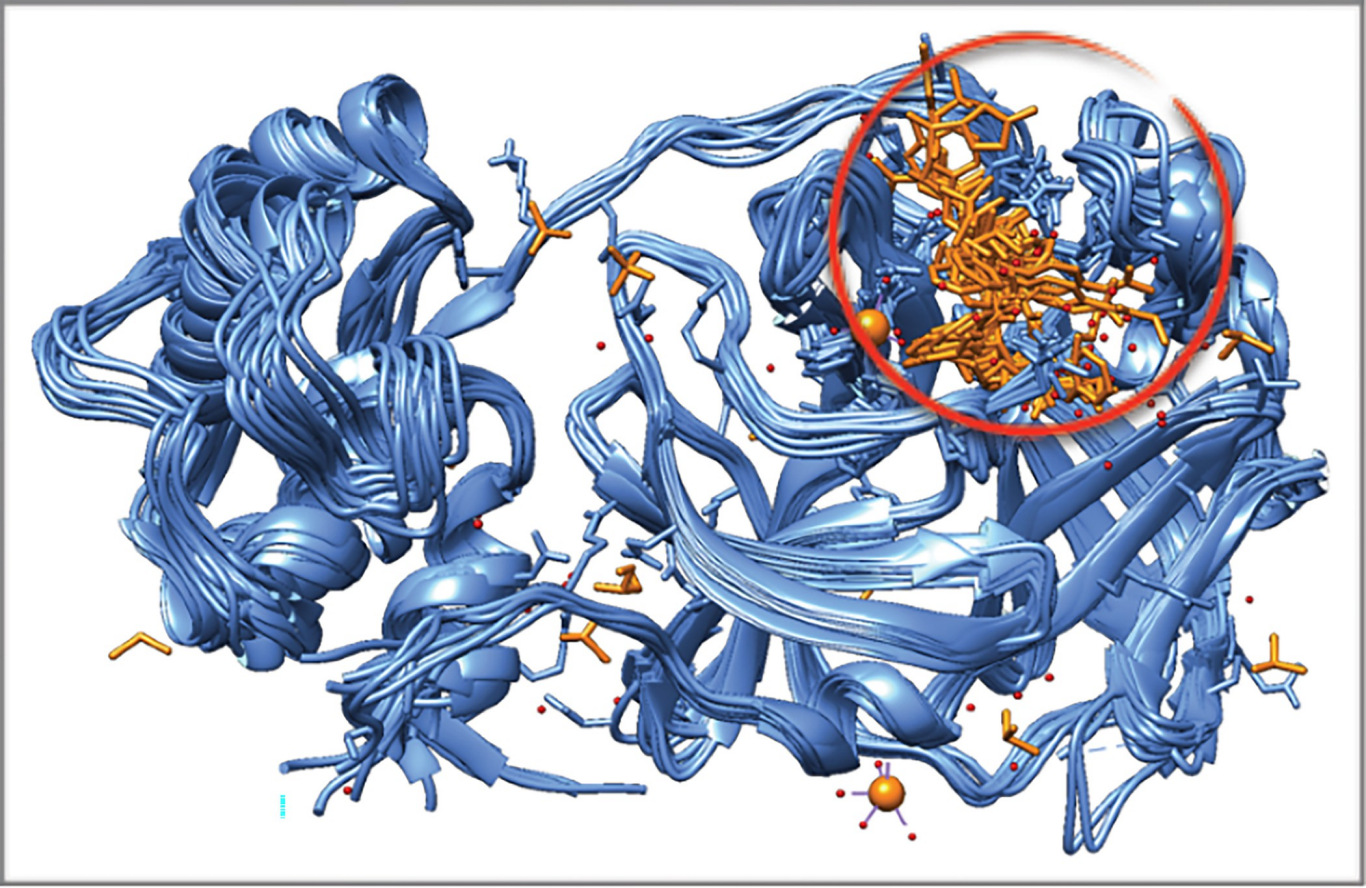
Ribbon representation of coronavirus M^pro^ structures in complex with ligands/inhibitors superposed, showing the main ligand binding site, using SARS-CoV-2 sequence as the query. The main ligands/inhibitors are circled in red. The water molecules are shown as small red balls. The figure was generated in Chimera. PDB information about the M^pro^ structures used for superposition in this figure are given in S1 Table in S1 File.

The ligand binding mode in each structure was then analysed by Ligplot (S2 Fig in S1 File) in order to identify the residues and type(s) of binding interactions involved (whether covalent, hydrogen bonding or hydrophobic interaction), also shown in S1 Table in S1 File. The Ligplot analysis showed that Cys145 was involved in covalent bonding in most of the ligand bound SARS- and MERS-related M^pro^ structures and even in gastroenteritis virus and human coronavirus M^pro^ structures, except for PDB IDs 4mds, 2op9, 2qiq (SARS-related M^pro^) and PDB ID 4ylu (MERS-related M^pro^), representing non-covalent inhibitors. Subsequently, the coronavirus structures were superposed (using Chimera) showing the presence of a common inhibitor binding site (Fig 1). As reported in S1 Table in S1 File, common residues involved in ligand binding via hydrogen bonding included the following residues (or their equivalent in terms of residue position): Phe140, Gly143, His163, His164, Glu166 and Gln189. Residue Cys145 (or its equivalent) was also seen to be involved in hydrogen bonding with ligands, as well as His41, which was shown to be involved in hydrogen bonding to ligands in some of the structures. The common residues involved in hydrophobic interactions included but was not limited to Thr25/Thr26, His41, Met49, Leu141, Asn142, Met165, Asp187 and Arg188. Residues such as Phe140, Gln189, and Cys145 were also involved in hydrophobic interactions in some of the M^pro^ ligand-bound structures. Additionally, it was clear that the ligand-bound M^pro^ structures most closely related to the SARS-CoV-2 M^pro^ sequence, had 7–8 hydrogen bondings and 14–16 hydrophobic interactions as well as a covalent bonding involving the Cys145 residue. It should be added here that SARS-CoV-2 M^pro^ has 12 cysteines occupying positions 16, 22, 38, 44, 85, 117, 128, 145, 156, 160, 265 and 300. Out of those, cysteines at positions 16, 38, 117 and 145 were identified to be conserved (S3 Fig in S1 File), amongst the coronavirus sequences analysed and aligned in this study (S1 Fig in S1 File).

### Comparison between experimentally derived structures of five synthetic covalent inhibitors bound to SARS-CoV-2 M^pro^ and that of molecular docking results

Five experimentally derived structures of SARS-CoV-2 M^pro^ with PDB IDs 6lu7, 6y2f, 6wtt, 6m0k and 6lze and corresponding synthetic covalent inhibitors, N3, alpha-ketoamide inhibitor (13b), GC-376, and peptidomimetic inhibitors 11a and 11b, respectively, were chosen in this study, based on their promising IC_50_ and EC_50_ values (S2 Table in S1 File).

As reported in S2 Table in S1 File, common residues involved in ligand binding via hydrogen bonding in all of the five structures included Phe140, Gly143 (except for PDB ID 6wtt), His163, His164 (except for PDB ID 6lu7) and Glu166. Residue Cys145 was also seen to be involved in hydrogen bonding in all structures as well as associated with all the inhibitors via covalent bonding. The most potent of inhibitors with an EC_50_ value of 0.53 ± 0.01 μM was related to PDB ID 6lze, where the following residues including His41, Met49, Tyr54, Leu141, Asn142, Ser144, Met165, His172, Asp187, Arg188 and Gln189, were involved in hydrophobic interactions with the ligand.

For the molecular docking analysis, the ligands from the five structures were taken out of the halo structures (which were all deposited in the monomeric state of SARS-CoV-2 M^pro^ in asymmetric unit, except for PDB ID 6wtt) and then docked using DockThor with the apo/unbound SARS-CoV-2 M^pro^ structure (PDB ID 6yb7; containing SARS-CoV-2 M^pro^ in the monomeric state of the protein in the asymmetric unit), and results presented in S3 Table in S1 File. N3 had the highest binding affinity to M^pro^ (-8.636 Kcal/mol) based on molecular docking analysis in this study. A comparative analysis of the results from the molecular docking and that of the actual solved structures revealed that residues involved in ligand binding in both experimentally solved structures and molecular docking results included His41, Asn142, Gly143, Cys145, Met165, Glu166 and Gln189. Covalent binding of Cys145 was not detected in the molecular docking results, but instead Cys145 was observed to be involved in hydrophobic interactions in all structures except for the M^pro^-alpha-ketoamide inhibitor (13b) complex, where Cys145 was not involved in the binding interaction at all (S3 Table in S1 File), contrary to the experimental data.

### Molecular docking of six antiviral drugs also used for COVID-19

Molecular docking analyses of six drugs used for treatment of COVID-19, targeting the SARS-CoV-2 M^pro^, including Remdesivir [28,29], Darunavir [30], Oseltamivir [29], Lopinavir [30,31], Ritonavir [29,30] and Ribavirin [32], were also performed using the DockThor program. The results revealed that the highest binding affinity was -8.894 Kcal/mol for Remdesivir followed by -8.399 Kcal/mol for Darunavir (S4 Table in S1 File). Remdesivir was found to bind to SARS-CoV-2 M^pro^ via hydrogen bonding to Glu166 and 9 hydrophobic interactions involving His41, Cys44, Phe140, Leu141, Asn142, Cys145, His163, Pro168 and Gln189. The binding interaction of Remdesivir through molecular docking seemed quite plausible since it involved common residues that have been identified in experimentally derived structures of potent inhibitors such as that of the peptidomimetic inhibitor(11a)-bound to SARS-CoV-2 M^pro^ (with the lowest EC_50_ value of 0.53 ± 0.01 μM; S2 Table in S1 File), including common residues His41, Phe140, Leu141, Asn142, Cys145, His163, Glu166 and Gln189.

### Molecular docking of 38 phytochemicals with SARS-CoV-2 M^pro^

The DockThor program was further used to evaluate the binding of 38 active constituents/phytochemicals from the following herbs/plants/trees including ginger, turmeric, elderberry, cinnamon, garlic, onion, black cumin, peppermint, broccoli, dill, black pepper, *Acanthopanax henryi* plant, Boswellia plant, Birch and Camphor tree (S5 and S6 Tables in S1 File), with the SARS-CoV-2 M^pro^. The active constituents/phytochemicals of the herbs/plants/trees were selected based on their anti-oxidative properties and antiviral effectiveness and benefits (using almost 300 supporting references; please see S5 and S6 Tables in S1 File).

Looking at the deposited structures of SARS-CoV-2 M^pro^, most had reported the monomeric state (i.e. the asymmetric unit of the crystal structures mainly contained a monomer of the SARS-CoV-2 M^pro^), with the homodimeric (dimeric) state deposited in some structures, which actually represents the biologically active form of SARS-CoV-2 M^pro.^ Therefore, in this study, the apo/unbound SARS-CoV-2 M^pro^ structures in the monomeric and dimeric states, with PDB IDs 6yb7 and 7ali, respectively, were both used for molecular docking. The emphasis on the DockThor outputs and the ranking of the compounds was based on affinity prediction values (Kcal/mol) of different ligands in virtual screening experiments considering the top-energy pose (according to Total Energy) of each compound, in both the monomeric and dimeric states of SARS-CoV-2 M^pro^ (Table 1A and 1B and S7 and S8 Figs in S1 File).

**Table 1:** Molecular docking results of top ranking compounds savinin, betulinic acid, and curcumin with (A) SARS-CoV-2 M^pro^ monomer and (B) SARS-CoV-2 M^pro^ dimer, respectively, based on affinity of binding. The top-energy pose (according to Total Energy) of each compound is presented (with an RMSD of 0.0). The DockThor molecular docking program was used for analysis of interactions between SARS-CoV-2 M^pro^ monomer and dimer and the three compounds, using default settings. Ligplot was used to reveal the residues involved in binding and the mode of interaction with the ligands. The SARS-CoV-2 M^pro^ monomer and SARS-CoV-2 M^pro^ dimer (chain A) used in this study were from PDB IDs 6yb7 and 7ali, respectively.

**(A) Molecular docking with SARS-CoV-2 M**^**pro**^ **monomer (PDB 6yb7)**				
**Herbs/plant/tree**	**Residues involved in hydrogen bonding (Å)**	**Residues involved in hydrophobic interactions**	**Number of amino acids involved in hydrophobic interactions**	**DockThor** **a) Affinity (Kcal/mol)** **b) Total energy** **c) Van der Waals Energy** **d) Electrostatic energy**
**Savinin** (***Acanthopanax henryi* plant)**	Gly143-N:Ligand-O2 (3.28) His163-NE2:Ligand-O5 (3.24)	Thr25; His41; Met49; Phe140; Leu141; Asn142; Ser144; Cys145; Met165; Glu166; Gln189	11	a) -8.864 b) 11.242 c) -28.605 d) -2.371
**Betulinic acid** (**Birch tree)**	-	Thr24; Thr25; Thr26; Cys44; Thr45; Ser46; Met49; Asn142; Gly143; Glu166; Gln189	11	-8.644 50.231 -18.759 -9.808
**Curcumin** **keto form** (**Turmeric)**	1. Thr26-N:Ligand-O5 (3.18)	Thr25; His41; Met49; His164; Met165; Glu166; Pro168; Gln189; Thr190; Gln192	10	-8.373 3.262 -24.451 -4.937
**(B) Molecular docking with SARS-CoV-2 M**^**pro**^ **dimer (PDB 7ali)**				
**Herbs/plant/tree**	**Residues involved in hydrogen bonding (Å)**	**Residues involved in hydrophobic interactions**	**Number of amino acids involved in hydrophobic interactions**	**DockThor** **a) Affinity (Kcal/mol)** **b) Total energy** **c) Van der Waals Energy** **d) Electrostatic energy**
**Curcumin** **keto form** (**Turmeric)**	Thr26-N:Ligand-O5 (3.04)	Thr25; Met49; Tyr118; Asn119; Asn142; Gly143; His164; Met165; Gln189	9	a) -8.554 b) 37.040 c) -24.955 d) -7.019
**Betulinic acid** (**Birch tree)**	-	Thr24; Thr25; Thr26; His41; Ser46; Met49; Asn119; Asn142; Gly143	9	-8.548 b) 129.576 c) 13.191 d) -1.699
**Savinin** (***Acanthopanax henryi* plant)**	-	Thr25; Cys44; Met49; Leu141; Asn142; Ser144; Cys145; His163; Glu166; Gln189	10	a) -8.518 b) 30.730 c) -25.170 d) -0.944

### Molecular docking using PDB ID 6y7b, containing the deposited monomeric state of SARS-CoV-2 M^pro^ in the asymmetric unit

**Fig 2:**
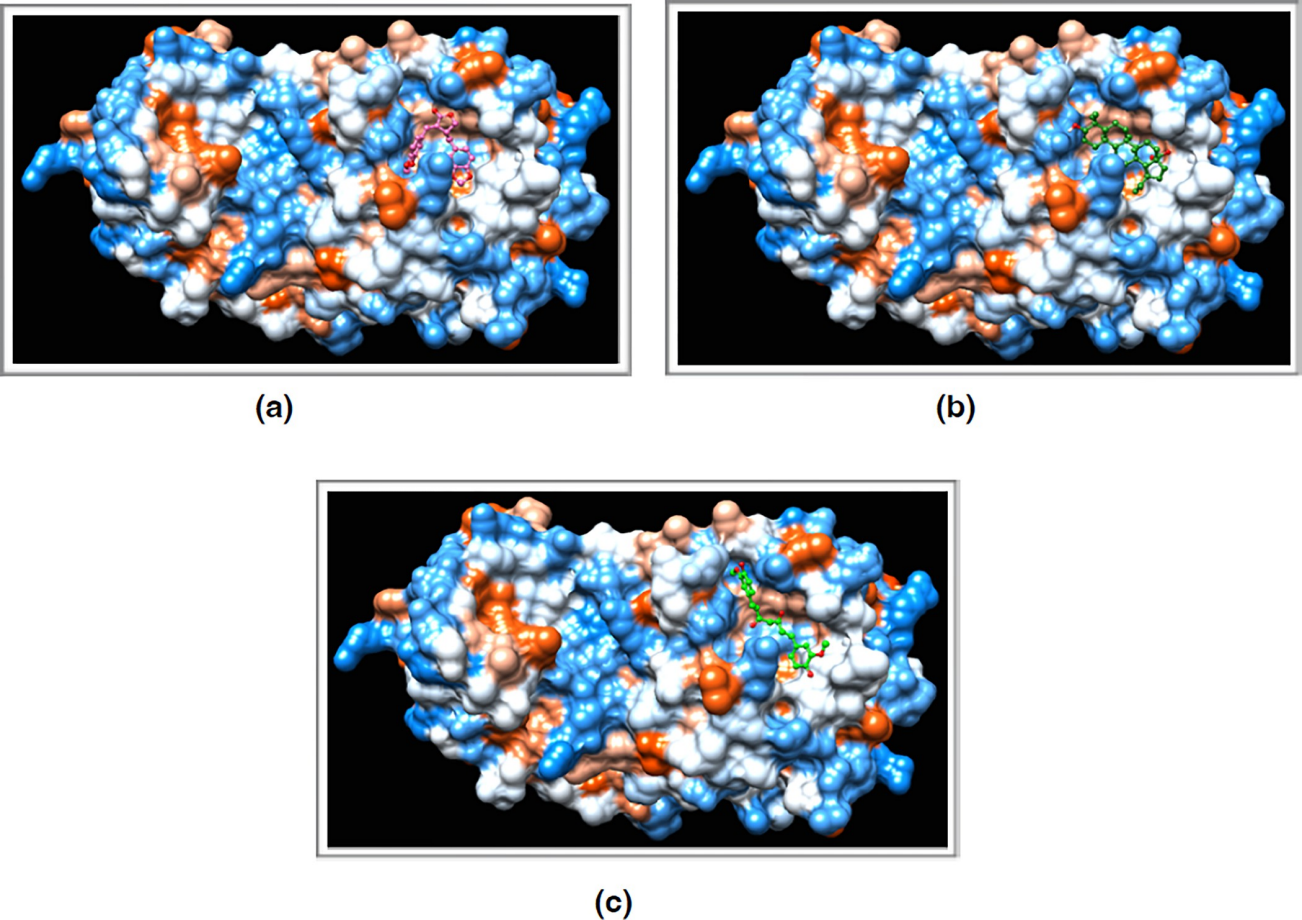
Hydrophobic surface representation of the highest scoring herbal compound-SARS-CoV-2 M^pro^ monomer complexes. **(a-c)** Savinin, betulinic acid and curcumin, respectively, in complex with SARS-CoV-2 M^pro^ monomer using DockThor. The phytochemicals are shown in ball and stick representation. The figure was generated using Chimera.

**Fig 3:**
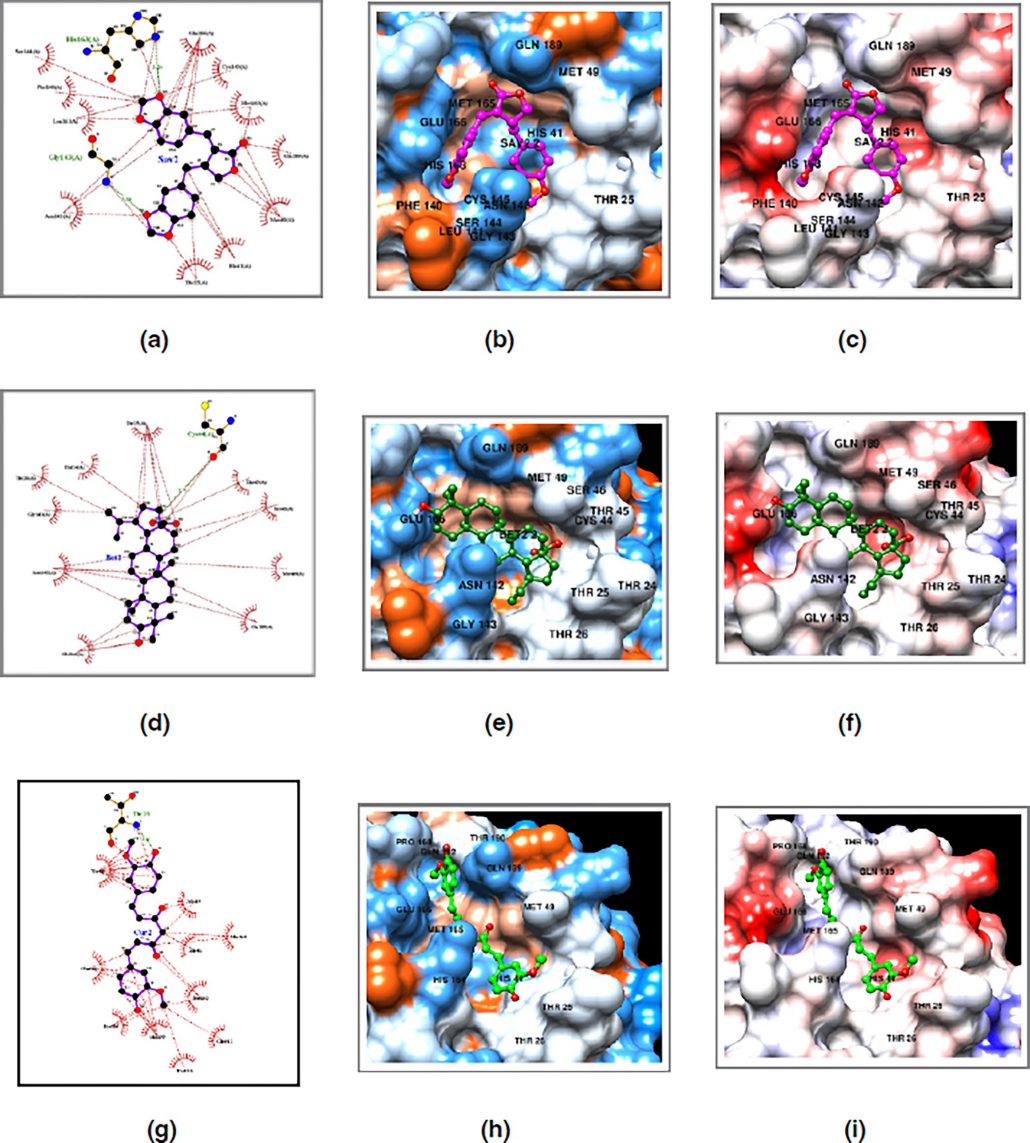
Structural analysis of savinin-, betulinic acid- and curcumin-SARS-CoV-2 M^pro^ monomer complexes as revealed by molecular docking, with the highest scores in terms of affinity. (a, d and g) Ligplot analyses of the savinin-, betulinic acid- and curcumin-SARS-CoV-2 M^pro^ monomer complexes, respectively, showing M^pro^ residues involved in hydrogen bonding and hydrophobic interactions. (b, e and h) Hydrophobic representation of the savinin-, betulinic acid- and curcumin-SARS-CoV-2 M^pro^ monomer complexes, respectively. (c, f and i) Electrostatic potential representation of the savinin-, betulinic acid- and curcumin-SARS-CoV-2 M^pro^ monomer complexes, respectively. The residues involved in interaction with savinin, betulinic acid and curcumin are labelled. The figure was generated using the Ligplot and Chimera programs.

The results of docking using PDB ID 6yb7, containing the monomeric state of SARS-CoV-2 M^pro^ in the asymmetric unit, showed that the phytochemical savinin from the *Acanthopanax henryi* plant and betulinic acid from birch tree had the highest two scores in terms of affinity (-8.864 and -8.644 Kcal/mol, respectively), followed by curcumin from turmeric (-8.373 Kcal/mol) (Table 1A and Figs 2 and 3). Referring to the savinin-SARS-CoV-2 M^pro^ molecular docking results analysed by LigPlot (Fig 3A) and Chimera’s hydrophobic and electrostatic interaction analyses (Fig 3B and 3C, respectively), savinin was bound to SARS-CoV-2 M^pro^ through two hydrogen bondings involving Gly143 and His163, and 11 hydrophobic interactions involving residues Thr25, His41, Met49, Phe140, Leu141, Asn142, Ser144, Cys145, Met165, Glu166 and Gln189. This seems a plausible interaction as savinin and the most potent synthetic SARS-CoV-2 inhibitor listed in S2 Table in S1 File, interacted with the following common residues (as shown through experimental data) including His41, Met49, Phe140, Leu141, Asn142, Gly143, Cys145, His163, Met165, Glu166 and Gln189. Additionally, the drugs mentioned in S4 Table in S1 File, and in particular Remdesivir, interacted with common residues similar to savinin, including His41, Phe140, Leu141, Asn142, Cys145, His163; Glu166 and Gln189. Additionally, according to a previous in silico docking study [33], where savinin was shown to bind to SARS-CoV-2 M^pro^ with a binding affinity of -8.3 kcal/mol (weaker binding affinity than that reported in this study), His163 (in addition to Ser144) was involved via hydrogen bonding (Gly143 was not reported in that study). Furthermore, similar residues including those identified in this study were involved in hydrophobic interactions with savinin, including His41, Phe140, Cys145, Met165 and Glu166 [33]. Out of the residues involved in binding interaction with savinin in our study, His41, Phe140, Gly143, Cys145, His163 and Glu166 were conserved amongst the native M^pro^ sequences analysed in this study (S1 Fig in S1 File).

As for the binding interaction of betulinic acid to SARS-CoV-2 M^pro^ analysed by LigPlot and Chimera (Fig 3D–3F), Cys44 was involved in hydrogen bonding and a total of 10 residues were involved in hydrophobic interactions including Thr24, Thr25, Thr26, Thr45, Ser46, Met49, Asn142, Gly143, Glu166 and Glu189. According to a previous in silico docking study [34], betulinic acid was found to inhibit SARS-CoV-2 M^pro^ with an IC_50_ value of 14.55 as well as a binding energy of −8.1 kcal/mol, and had exhibited hydrogen bonding with His41 and Phe140 (this is different to what we have seen in this current study). Out of the residues involved in binding interaction with betulinic acid, Cys44 and Glu166 were the two residues conserved amongst the native M^pro^ sequences analysed in this study (S1 Fig in S1 File).

Additionally, the analysis of binding interactions of curcumin to SARS-CoV-2 M^pro^ by LigPlot and Chimera (Fig 3G–3I), revealed that Thr26 was involved in hydrogen bonding and a total of 10 residues were involved in hydrophobic interactions including Thr25, His41, Met49, His164, Met165, Glu166, Pro168, Gln189, Thr190 and Gln192. Out of the residues involved in binding interaction with curcumin, His41, Glu166 and Gln192 were the three residues conserved amongst the native M^pro^ sequences analysed in this study (S1 Fig in S1 File).

### Molecular docking using PDB ID 7ali, containing the dimeric and biologically active form of SARS-CoV-2 M^pro^

**Fig 4:**
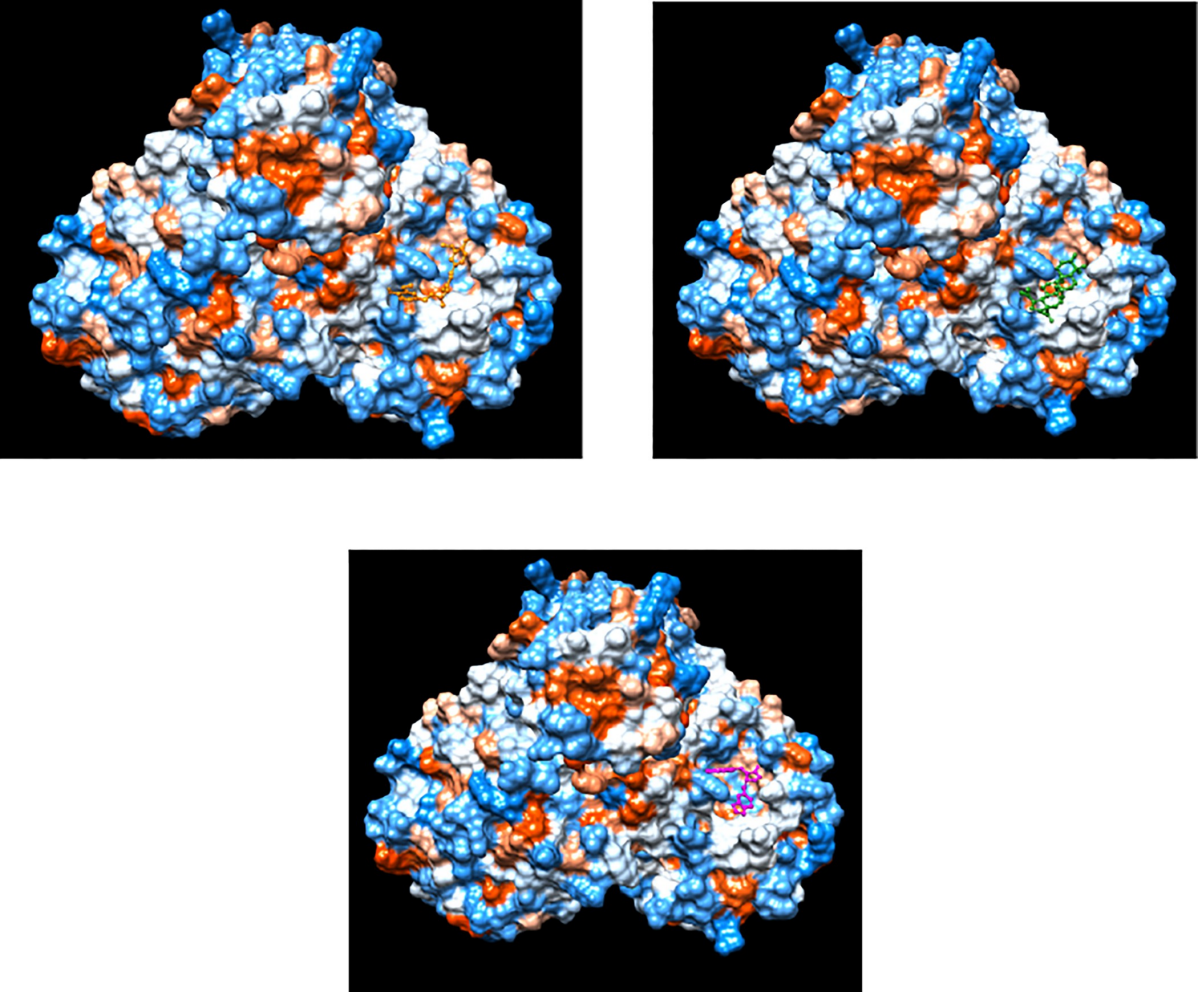
Hydrophobic surface representation of the highest scoring herbal ligand-SARS-CoV-2 M^pro^ dimer complexes. (a-c) Curcumin, betulinic acid and savinin, respectively, in complex with SARs-CoV-2 M^pro^ dimer using DockThor. The herbal ligands are shown in ball and stick representation. The figure was generated using Chimera.

**Fig 5:**
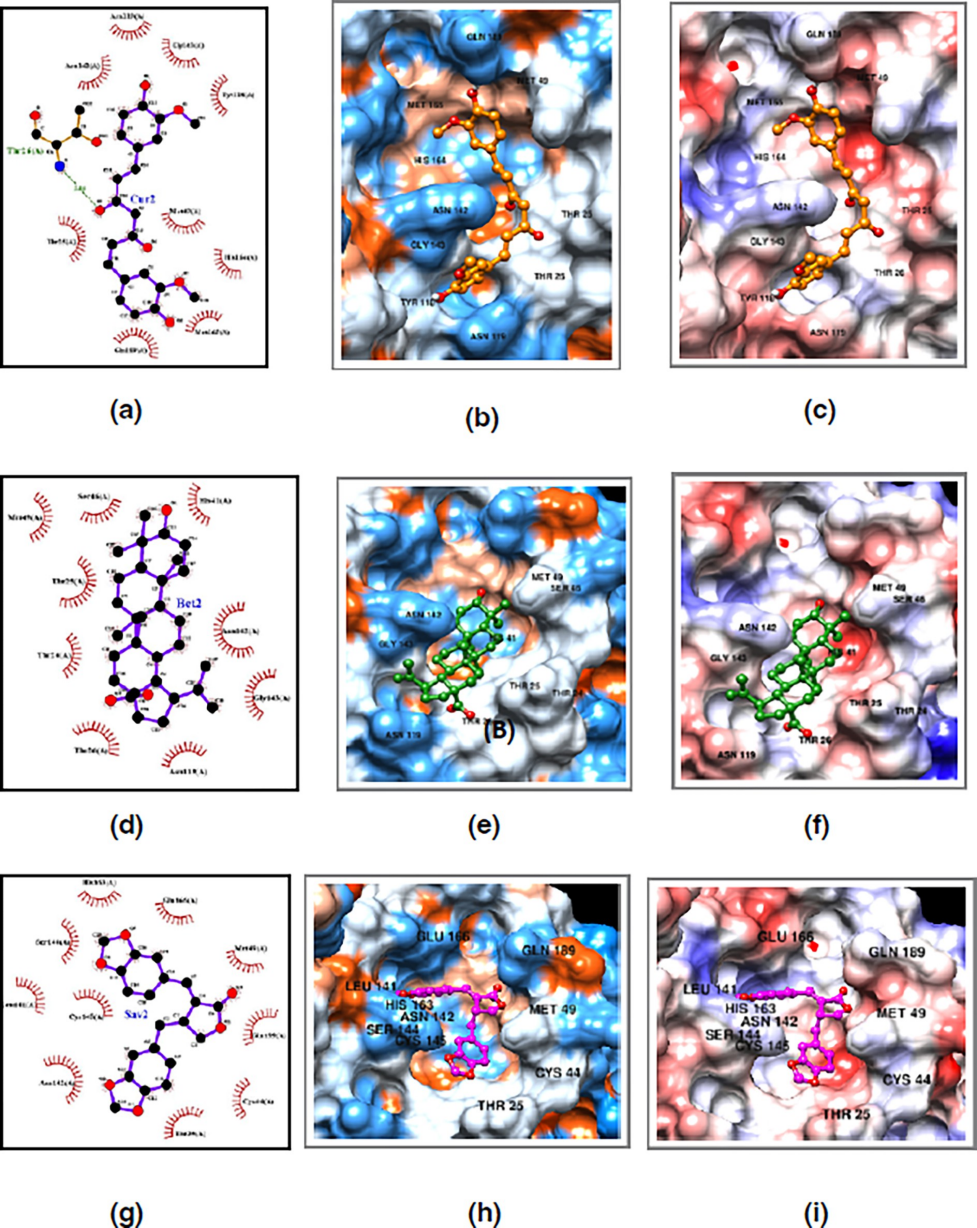
Structural analysis of the curcumin- and betulinic acid- and savinin-SARS-CoV-2 M^pro^ dimer complexes as revealed by molecular docking, with the highest scores in terms of affinity. **(**a, d and g) Ligplot analyses of the curcumin- and betulinic acid- and savinin-SARS-CoV-2 M^pro^ dimer complexes, respectively, showing M^pro^ residues involved in hydrogen bonding and hydrophobic interactions. (b, e and h) Hydrophobic representation of the curcumin-, betulinic acid- and savinin-SARS-CoV-2 M^pro^ dimer complexes, respectively. (c, f and i) Electrostatic potential representation of curcumin-, betulinic acid- and savinin-SARS-CoV-2 M^pro^ dimer complexes, respectively. The residues involved in interaction with either curcumin, betulinic acid or savinin are labelled. The figure was generated using the Ligplot and Chimera programs.

The results of molecular docking using PDB ID 7ali, containing the dimeric state of SARS-CoV-2 M^pro^, showed that in contrary to the monomeric state of M^pro^ where savinin, betulinic acid and curcumin had the highest consecutive scores in terms of affinity, instead curcumin ranked first with the highest score in terms of affinity, followed by betulinic acid and savinin (-8.554, -8.548 and -8.518 Kcal/mol, respectively) (Table 1B and Figs 4 and 5). Having said that, the binding affinities were very close to each other.

Curcumin was bound to SARS-CoV-2 M^pro^ through a hydrogen bonding with Thr26 and 9 hydrophobic interactions involving residues Thr25, Met49, Tyr118, Asn119, Asn142, Gly143, His164, Met165 and Gln189 (Fig 5A–5C). Compared to curcumin bound to the monomeric state of SARS-CoV-2 M^pro^, the common residues included Thr26 (via hydrogen bonding), Thr25, Met49, Asn142, His164, Met165 and Gln189. Residue Cys145 was not seen in the binding interaction with curcumin, neither in the monomeric nor the dimeric states of SARS-CoV-2 M^pro^.

There were no hydrogen bondings for docking results of betulinic acid and savinin bound to SARS-CoV-2 M^pro^ in the dimeric state. Both ligands appeared to bind via 9 and 10 hydrophobic interactions, respectively. Residues involved in binding to betulinic acid included Thr24, Thr25, Thr26, His41, Ser46, Met49, Asn119, Asn142 and Gly143 (Fig 5D–5F). Compared to the docking results using the monomeric state of SARS-CoV-2 M^pro^, the common residues involved in hydrophobic interactions included Thr24, Thr25, Thr26, Ser46, Met49, Asn142 and Gly143. Consistent with curcumin-bound to SARS-CoV-2 M^pro^, Cys145 was not involved in binding to betulinic acid, neither in the monomeric nor dimeric states of SARS-CoV-2 M^pro^. As for savinin, the following residues were involved in the hydrophobic interactions: Thr25, Cys44, Met49, Leu141, Asn142, Ser144, Cys145, His163, Glu166 and Gln189 (Fig 5G–5I). Compared to the molecular docking results using the monomeric state of SARS-CoV-2 M^pro^, the common residues involved in hydrophobic interactions included Thr25, Met49, Asn142, Ser144, Cys145, Glu166 and Gln189. Residue Cys145 was present in hydrophobic bonding interactions to savinin in both the monomeric and dimeric states of SARS-CoV-2 M^pro^.

Referring to S9 Table in S1 File, all of the three phytochemicals including savinin, betulinic acid and curcumin, with the highest scores in terms of binding affinity, using both the monomeric and dimeric states of SARS-CoV-2 M^pro^, were also listed to have antioxidant/hot-temperament based properties, as well as antiviral effects; savinin and betulinic acid, in particular, were reported to have antiviral effects against SARS-CoV-2 [33,34].

### Re-docking of savinin, betulinic acid and curcumin

As a means of gaining more confidence in our results from molecular docking, re-docking of the best pose of savinin, betulinic acid and curcumin with SARS-CoV-2 M^pro^ were performed in DockThor and results revealed the following RMSD values including 0.233 Å, 0.041 Å and 0.433 Å, respectively. As can be clearly observed, the betulinic acid pose for binding to SARS-CoV-2 M^pro^ had the lowest and hence the best re-docking RMSD value, followed by savinin and curcumin.

### Covalent versus non-covalent SARS-CoV-2 M^pro^ inhibitors

As stated previously, SARS-CoV-2 M^pro^ includes a Cys145-His41 catalytic dyad (where the ionization of sulfhydryl group of Cys145 and imidazole group of His41 play an important role in the catalytic processing of substrate hydrolysis), and a cleft between domains I and II, where the inhibitor/substrate binding site is located. His41 and Cys145 were shown to be involved in savinin binding to the SARS-CoV-2 M^pro^ (in the monomeric state) and helps to further recommend savinin as an effective covalent inhibitor. Furthermore, although DockThor failed to reveal the covalent binding of Cys145 to the ligands of confirmed covalent inhibitors, there is a high probability that Cys145 is involved in covalent interaction with savinin. Having said that, non-covalent inhibitors also exist such as that seen in PDB ID 7l0d, with the inhibitor ML188 bound to SARS-CoV-2 M^pro^ (deposited in the monomeric state). Non-covalent inhibitors, like ML188, may actually be more potent than covalent inhibitors [35]. Therefore, it is not necessary to have a covalent interaction between Cys145 and the inhibitor in order to have a strong inhibition against SARS-CoV-2 M^pro^. S10 Table in S1 File shows that the non-covalent inhibitor ML188 binds to SARS-CoV-2 M^pro^ via four hydrogen bondings involving Asn142, Gly143 (with two hydrogen bondings) and His163. Additionally, 14 residues are involved in hydrophobic interactions including Thr25, Thr26, Leu27, His41, Met49, Leu141, Phe140, Ser144, Cys145, His164, Glu166, Asp187, Arg188 and Gln189. As can be clearly seen, both the His41 and Cys145 residues are involved in the binding but via hydrophobic interactions. Molecular docking of ML188 with PDB ID 6yb7 (monomeric state of SARS-CoV-2 M^pro^, same as that of the experimentally observed deposited state of SARS-CoV-2 M^pro^ in PDB ID 7l0d), for comparison, revealed a high binding affinity of -8.883 Kcal/mol (S11 Table in S1 File), showing two hydrogen bondings involving His163 and Glu166 and 9 hydrophobic interactions involving residues including His41, Ser46, Phe140, Asn142, Gly143, Cys145, His164, Met165 and Gln189. The common residues between the experimentally derived structure of the ML188-SARS-CoV-2 M^pro^ complex and that the of the molecular docking result, included His41, Asn142, Gly143, His163, Cys145, His146 and Gln189 (through either hydrogen bonding/hydrophobic interactions).

### Nirmatrelvir as an approved specific inhibitor of SARS-CoV-2 M^pro^

To make the results of this study more meaningful, the structure of Nirmatrelvir bound to SARS-CoV-2 M^pro^ (PDB ID 7te0), as an approved inhibitor of the only FDA approved drug against COVID-19, PAXLOVID, regarded as a combination therapy of nirmatrelvir and ritonavir, was used in this study [27].

Nirmatrelvir has been reported (S10 Table in S1 File) to bind to Cys145, His163, Glu166 and Gln192 through hydrogen bonding. Nirmatrelvir also binds to Cys145 via a covalent interaction. Additionally, 8 residues are involved in hydrophobic interactions with Nirmatrelvir including His41, Met49, Phe140, Asn142, Gly143, Met165, His172 and Gln189. Molecular docking of Nirmatrelvir with PDB ID 6yb7 (monomeric state of SARS-CoV-2 M^pro^, same as that of the experimentally observed deposited state of SARS-CoV-2 M^pro^ in PDB ID 7te0), for comparison, revealed a binding affinity of -7.793 Kcal/mol (S11 Table in S1 File), showing a hydrogen bonding with Gly143 and 9 hydrophobic interactions involving residues Ser46, Phe140, Asn142, Cys145, His163, Glu166, Leu167, Pro168 and Gln189. The common residues between the experimentally derived structure of the Nirmatrelvir-SARS-CoV-2 M^pro^ complex and that the of the molecular docking result (using the monomeric state of SARS-CoV-2 M^pro^), included residues Phe140, Gly143, Asn142, Cys145, His163, Glu166 and Gln189 (through either hydrogen bonding/hydrophobic interactions). Additional molecular docking using the dimeric state of SARS-CoV-2 M^pro^ (PDB ID 7ali) revealed a stronger binding affinity of -8.404 Kcal/mol and the involvement of Glu166 in hydrogen bonding as well as 11 residues involved in hydrophobic interactions, including Thr25, Thr26, Leu27, His41, Ser46, Met49, Asn119, Asn142, Gly143, Cys145 and Gln189 (S11 Table in S1 File). The common residues between the experimentally derived structure of the Nirmatrelvir-SARS-CoV-2 M^pro^ complex and that the of the molecular docking result (using the dimeric state of SARS-CoV-2 M^pro^), included residues His41, Met49, Asn142, Gly143, Cys145, Glu166 and Gln189 (through either hydrogen bonding/hydrophobic interactions).

### ADMET analysis

The four ligands including savinin, betulinic acid, curcumin and Nirmatrelvir (as the reference ligand in this study) were all evaluated using the ADMETlab2.0 online program [36]. A summary of some of the calculated parameters for each ligand are given in S12 Table in S1 File. Three rules under the category of medicinal chemistry, described as Lipinski, Pfizer and GSK rules, were assessed in addition to the parameters shown in S12 Table in S1 File. Nirmatrelvir, satisfied many of the factors relating to the analysis in addition to the Lipinski and Pfizer rules. Curcumin satisfied all the three rules and had the highest favourable calculated values for the parameters evaluated compared to savinin and betulinic acid (S12 Table in S1 File). Savinin was accepted by the Lipinski and GSK rules, while betulinic acid was accepted by the Lipinski rule only. The golden triangle (where a molecular weight of between 50 or less and 200 or more, with a logD of less than or equal to 5 indicated a more favourable ADMET profile) revealed that only Nirmatrelvir, savinin and curcumin satisfied the criteria.

### Validation of the molecular docking results

To validate the results of molecular docking, MD simulation analyses including calculations of RMSD, RMSF, Rg and number of hydrogen bonds,BSA analysis and MM-PBSA, were performed for savinin-, betulinic acid- and curcumin-SARS-CoV-2 M^pro^ complexes as well as Nirmatrelvir-SARS-CoV-2 M^pro^ complex, as a means to make a comparison with the only approved inhibitor of SARS-CoV-2 M^pro^. The validation was performed using both the monomeric and dimeric states of SARS-CoV-2 M^pro^, both in the apo and halo forms.

### MD simulation analysis of the complexes

MD simulation, a method to investigate the movements of atoms and molecules over a given period of time, was performed to discover the stability and intermolecular interactions of the phytochemicals and Nirmatrelvir with the SARS-CoV-2 M^pro^ (in both the monomeric and dimeric states).

### RMSD anlaysis

**Fig 6:**
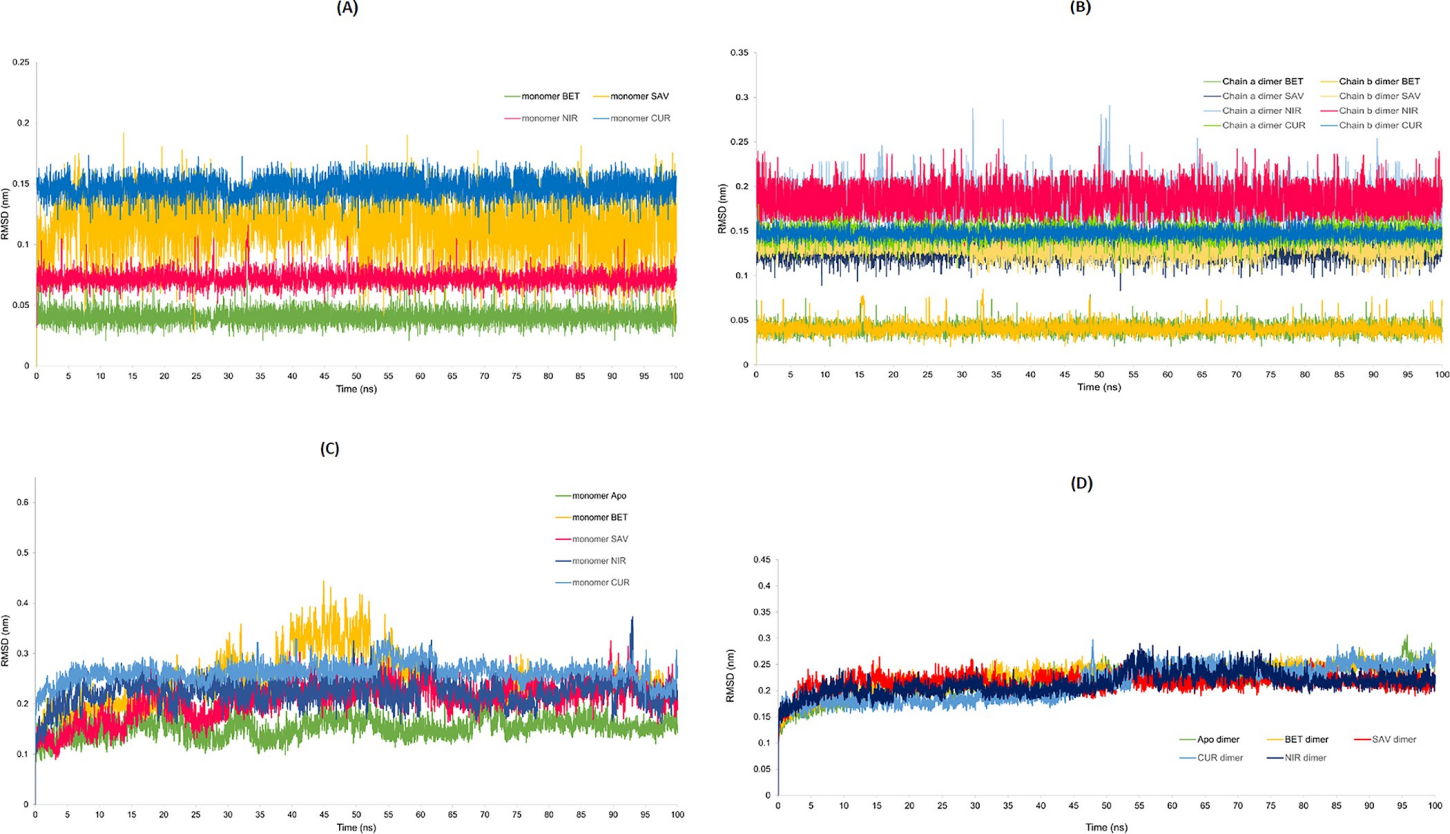
RMSD plots of ligands from both monomeric and dimeric states of SARS-CoV-2 M^pro^ and the RMSD plots of the apo and ligand-bound SARS-CoV-2 M^pro^ complexes in the dimeric state. (A) RMSD of the ligands from the ligand-monomeric SARS-CoV-2 M^pro^ complexes. Betulinic acid (green) showed the highest stability followed by Nirmatrelvir (red), Savinin (yellow) and Curcumin (navy blue). (B) RMSD of the ligands from the ligand-dimeric SARS-CoV-2 M^pro^ complexes. Both chains A and B of Betulinic acid, Savinin, Curcumin and Nirmatrelvir are presented. (C) RMSD of the ligand-monomeric SARS-CoV-2 M^pro^ complexes. (D) RMSD of the ligand-dimeric SARS-CoV-2 M^pro^ complexes. Apo SARS-CoV-2 M^pro^ (green); Betulinic acid-SARS-CoV-2 M^pro^ (yellow), Savinin-SARS-CoV-2 M^pro^ (red), Nirmatrelvir-SARS-CoV-2 M^pro^ (dark blue) and curcumin-SARS-CoV-2 M^pro^ (light blue). Apo-SARS-CoV-2 M^pro^ (Apo), Betulininc acid-SARS-CoV-2 M^pro^ complex (BET), Savinin-SARS-CoV-2 M^pro^ complex (SAV), Curcumin-SARS-CoV-2 M^pro^ complex (CUR) and Nirmatrelvir-SARS-CoV-2 M^pro^ complex (NIR).

RMSD, an indicator of the stability of the structures during simulation, was calculated for the backbone atoms of apo- and ligand bound-SARS-CoV-2 M^pro^ structures. The RMSD values of the ligands from the ligand-monomeric SARS-CoV-2 M^pro^ complexes converged over a range of 0.10–0.45 nm (Fig 6A), while, the RMSD of the ligands from the ligand-dimeric SARS-CoV-2 M^pro^ complexes converged over a range of 0.15–0.3 nm (Fig 6B). As seen in Fig 6A and 6B, the RMSD plots of the ligands through MD simulation of both monomeric and dimeric states of SARS-CoV-2 M^pro^ showed betulinic acid and curcumin with the lowest and highest RMSD values, respectively. The RMSD values of Nirmatrelvir bound to SARS-CoV-2 M^pro^ in the dimeric state exhibited higher RMSD values than curcumin. Overall, the presented results indicated the stability of the complexes and ligands during the MD simulations. Despite the stable RMSD values of the betulinic acid-, savinin- and Nirmatrelvir-SARS-CoV-2 M^pro^ dimer complexes and the apo dimer, the curcumin-SARS-CoV-2 M^pro^ dimer experienced a 10 nm rise, at the mid-point of the 45 ns to 55 ns period (Fig 6D). As for the RMSD of monomeric complexes and the apo monomer, a greater rise was observed in the RMSD value for the betulinic-SARS-CoV-2 M^pro^ monomer complex (Fig 6C).

**Fig 7:**
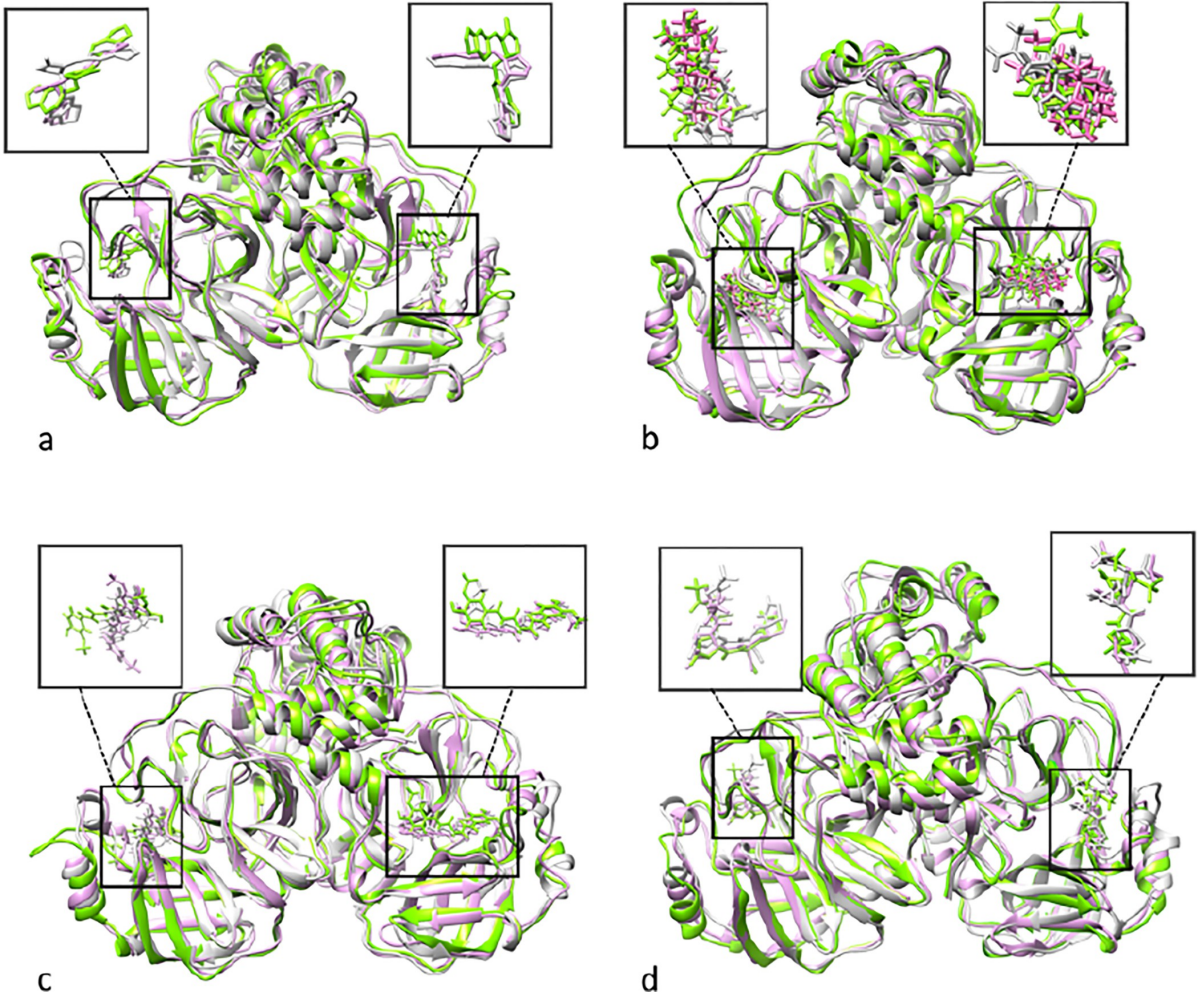
Overlay of ligand-dimeric SARS-CoV-2 M^pro^ complexes at 0, 50 and 100 ns intervals of MD simulations. (a) Savinin- SARS-CoV-2 M^pro^ complex, (b) Betulinic acid-SARS-CoV-2 M^pro^ complex, (c) Curcumin-SARS-CoV-2 M^pro^ complex and (d) Nirmatrelvir-SARS-CoV-2 M^pro^ at 0, 50 and 100 ns (in grey, pink and green, respectively) of the MD simulation.

Fig 7 represents the overlay of the ligand-dimeric SARS-CoV-2 M^pro^ complexes at 0, 50 and 100 ns intervals of MD simulations. The overlayed structures of the dimers revealed that the positions of the monomers remained consistent relative to each other.

### RMSF analysis

**Fig 8:**
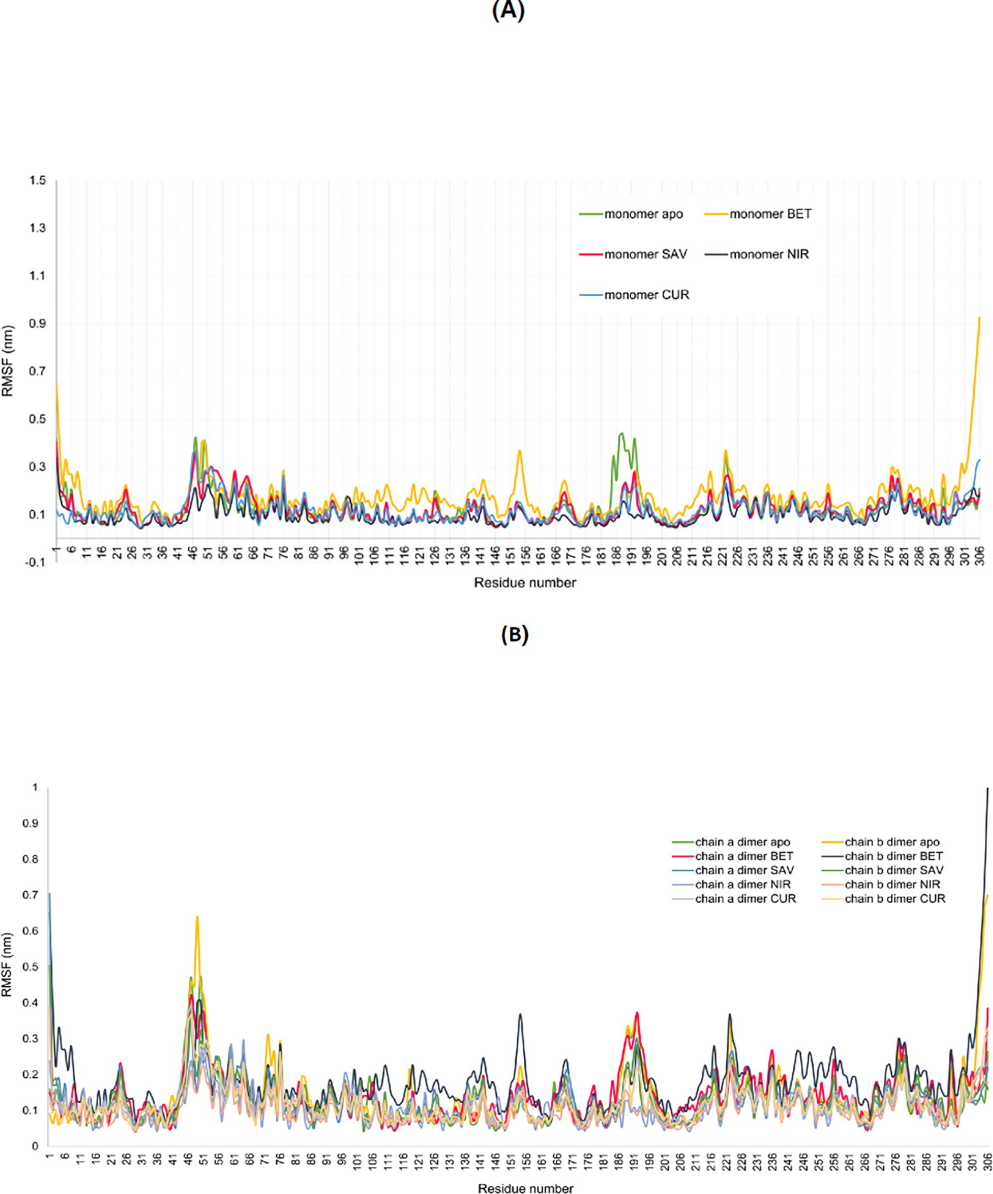
RMSF analysis of apo- and ligand-bound SARS-CoV-2 M^pro^ structures, in both the monomeric and dimeric states. (A) RMSF values of apo- and ligand- bound SARS-CoV-2 M^pro^ structures, in the monomeric state. (B) RMSF values of apo- and ligand-bound SARS-CoV-2 M^pro^ structures, in the dimeric state. Chains A and B of SARS-CoV-2 M^pro^ in the dimeric state were analysed separately.

The RMSF analysis was used to investigate fluctuation in the residues during the simulation. The overall RMSF values for apo- and ligand-bound SARS-CoV-2 M^pro^ structures, in the dimeric state were relatively similar, however, Met49 showed lower fluctuation when stabilised by the ligands (Fig 8). RMSF values of betulinic acid was higher than other complexes denoting the higher fluctuation of the conformation. ΔRMSF of apo- and Nirmatrelvir-SARS-CoV-2 M^pro^ complex for Met49 was 0.495 nm, indicating the higher stability of this residue in the presence of Nirmatrelvir. ΔRMSF of Met49 in apo- and betulinic acid-SARS-CoV-2 M^pro^ complex was 0.344 nm. This suggests that Nirmatrelvir reduced the fluctuation of Met49, more than betulinic acid. Similarly, Tyr154 was highly unstable in the betulinic acid-SARS-CoV-2 M^pro^ complex, while other complexes experienced lower fluctuations. Overall, RMSF values indicated that all complexes had a similar behaviour during the MD simulation, in both the monomeric and dimeric states of SARS-CoV-2 M^pro^ (Fig 8A and 8B).

### Rg analysis

**Fig 9:**
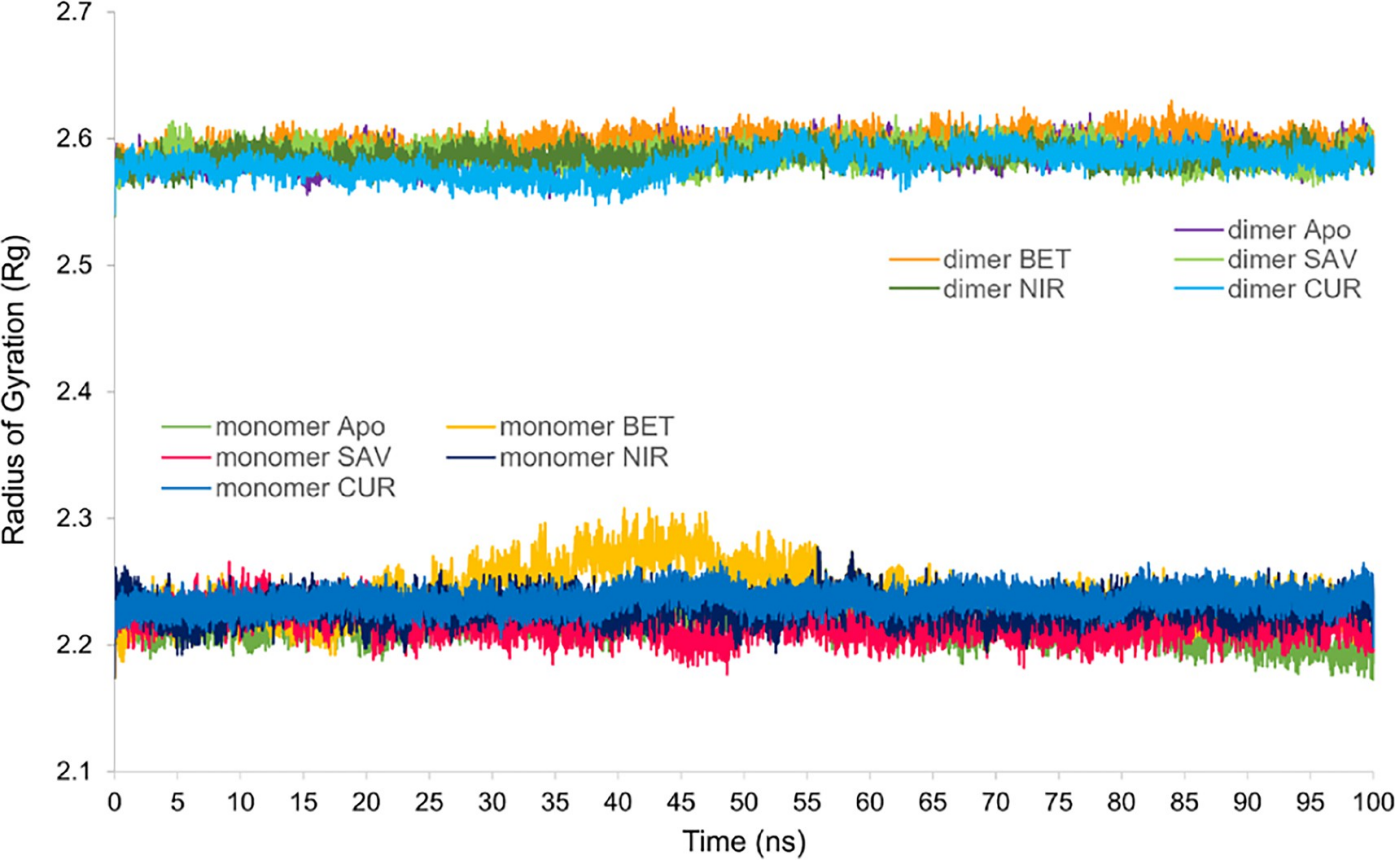
Radius of Gyration of apo- and ligand-bound SARS-CoV-2 M^pro^ structures, both in the monomeric and dimeric states. Apo-SARS-CoV-2 M^pro^ (Mpro), Betulininc acid-SARS-CoV-2 M^pro^ complex (BET), Savinin-SARS-CoV-2 M^pro^ complex (SAV), Curcumin-SARS-CoV-2 M^pro^ complex (CUR) and Nirmatrelvir-SARS-CoV-2 M^pro^ complex (NIR). Dimer denotes the dimeric form of the SARS-CoV-2 M^pro^ protein, either in apo or halo forms.

The Rg analysis measures the degree of structural compactness against time, where conformational stability of the protein maintains a steady value of Rg and misfolding increases the Rg. Fluctuation of the Rg values, between 2.56 and 2.63 nm, indicated that the conformation of all the apo- and ligand-SARS-CoV-2 M^pro^ complex structures, in the dimeric state, were reserved during the simulation (Fig 9). The curcumin-SARS-CoV-2 M^pro^ complex, in the dimeric state, had experienced a 0.1 nm rise, between 40 and 50 ns, which was consistent with the rise observed in RMSD values (Fig 6C). The apo- and ligand-SARS-CoV-2 M^pro^ complex structures, in the monomeric state, presented a lower fluctuation range, between 2 to 2.30 nm, confirming the conformational stability of the structures (Fig 9).

### Number of hydrogen bonds

**Fig 10:**
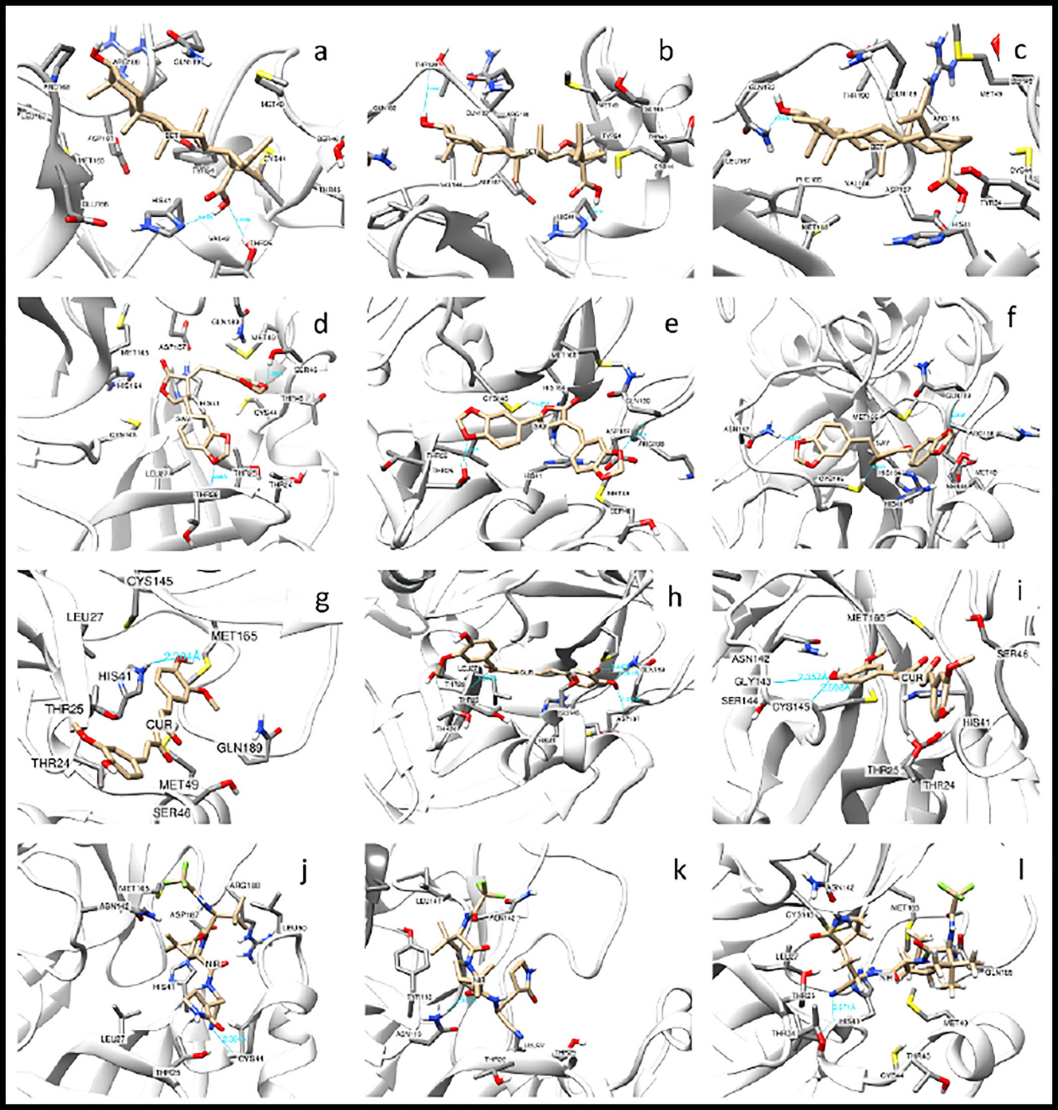
Hydrogen bonding analysis between the ligands and the monomeric state of SARS-CoV-2 M^pro^ as revealed by MD. (a, b and c) Hydrogen bonds formed between betulinic acid and SARS-CoV-2 M^pro^. ^(^d, e and f) Hydrogen bonds formed between savinin and SARS-CoV-2 M^pro^. (g, h and i) Hydrogen bonds formed between curcumin and SARS-CoV-2 M^pro^. (j, k and l) Hydrogen bonds formed between Nirmatrelvir and SARS-CoV-2 M^pro^. (a) Thr25 and His41, (b) Thr190 and His41 and (c) Gln192 and His41 participated in hydrogen bonding with betulinic acid. (d) Ser46 and main chain of Thr26, (e) Thr25, Cys145 and main chain of Gln189 and (f) Asn142, Cys145 and main chain of Gln189 formed hydrogen bonded with savinin. (g) His41, (h) Gln189, Asp187, Thr26 and (i) Gly143 and Cys145 formed hydrogen bonds with curcumin. (j) Cys44, (k) Asn119 and (l) His41 hydrogen bonded with Nirmatrelvir.

**Fig 11:**
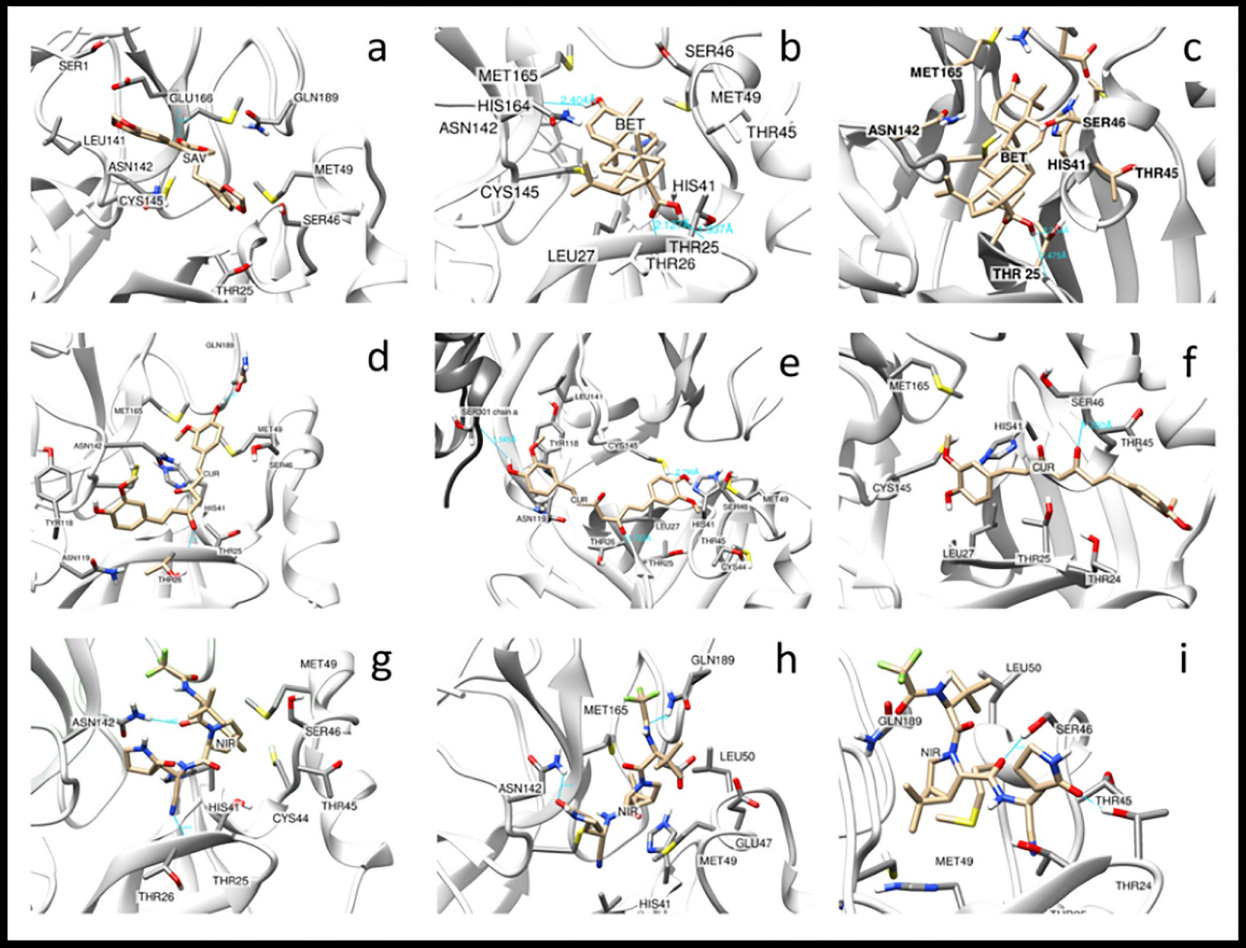
Hydrogen bonding analysis between the ligands and the dimeric state of SARS-CoV-2 M^pro^ as revealed by MD. (a) Hydrogen bonding formed between savinin and Glu166 of SARS-CoV-2 M^pro^. (b and c) Hydrogen bondings formed between betulinic acid and residues Thr25, Thr26 and His164 of SARS-CoV-2 M^pro^. (d, e and f) Hydrogen bondings formed between curcumin and residues Thr26 and Gln189 of SARS-CoV-2 M^pro^, as well as Ser301 of the adjacent monomer. (g, h and i) Hydrogen bondings formed between Nirmatrelvir and residues Thr25, Thr45, Ser46, Asn142 and Gln189 of SARS-CoV-2 M^pro^.

As shown in Figs 10 and 11, multiple hydrogen bondings were formed between both the monomeric and dimeric states of SARS-CoV-2 M^pro^ and the ligands, respectively. In MD simulation of betulinic acid-SARS-CoV-2 M^pro^ complex (using the monomeric state of SARS-CoV-2 M^pro^), hydrogen bonds with Thr25, His41, Thr190 and Gln192 were observed (Fig 10A–10C). Additionally, the MD simulation of betulinic acid-SARS-CoV-2 M^pro^ using the dimeric state of SARS-CoV-2 M^pro^ suggested that His164 participated in the formation of a hydrogen bond and double hydrogen bonds were formed with Thr25 side chain and an additional hydrogen bond with Thr24 main chain (Fig 11B and 11C). The MD simulation results of savinin-SARS-CoV-2 M^pro^ complex in the monomeric state presented hydrogen bonding with side chains of Thr25, Thr26, Ser46, Asn142, Cys145 and main chain of Gln189 (Fig 10D–10F). The savinin-SARS-CoV-2 M^pro^ complex in the dimeric state revealed a hydrogen bonding between Glu166 and savinin (Fig 11A). In MD simulation of the curcumin-SARS-CoV-2 M^pro^ complex in the monomeric state, hydrogen bonds were observed between His41 (Fig 10G), main chain of Asp187, Gln189 and main chain of Thr26 (Fig 10H), and main chain of Gly143 and Cys145 (Fig 10I). In the curcumin-SARS-CoV-2 M^pro^ complex in the dimeric state, hydrogen bonds were formed between curcumin and residues Thr25, Thr26, Ser46, Cys145, Gln189 and Ser301 of the adjacent monomer (Fig 11D–11F). As for the MD results of Nirmatrelvir-monomeric SARS-CoV-2 M^pro^ complex, hydrogen bondings were formed between Nirmatrelvir and residues His41, Cys44 and Asn119 (Fig 10J–10L). Nirmatrelvir-dimeric SARS-CoV-2 M^pro^ complex exhibited hydrogen bonds formed between Nirmatrelvir and residues Thr26, Thr45, Ser46, Asn142 and Gln189, (Fig 11G–11I).

### Binding free energies and residue contributions

To investigate the overall contributions of the residues to the binding energy of the ligands to the dimeric state of SARS-CoV-2 M^pro^, binding free energy calculations were performed. Using g_mmpbsa package [37,38], Van der Waal, electrostatic, polar, solvent-accessible surface area (SASA) and the total binding free energies, were calculated for the dimeric complexes for 100 ns MD simulations (Table 2). The highest binding energy belonged to betulinic acid at -83.579 kJ/mol, while savinin had the lowest binding energy. Nirmatrelvir with a total binding energy of -60.521 kJ/mol, had the highest binding energy after betulinic acid. Curcumin, with a total binding energy of -50.588 kj/mol, higher than the binding energy for savinin (-48.284 kJ/mol), had a lower total binding energy compared to Nirmatrelvir. Despite the unfavoured effect of polar interactions for the binding of the ligands, hydrophobic non-polar interactions played the main role in ligand binding. These data suggested that betulinic acid might be a more effective inhibitor for SARS-CoV-2 M^pro^.

**Table 2:** Summary of the MM/PBSA results of the ligand-bound SARS-CoV-2 M^pro^ dimeric complexes. The values are given in kJ/mol.

	Savinin	Betulinic acid	Curcumin	Nirmatrelvir
**van der Waal energy**	-76.413 +/- 11.956	-139.438 +/- 18.895	-87.981 +/- 49.483	-112.561 +/- 29.193
**Electrostattic energy**	-11.433 +/- 6.398	-15.829 +/- 4.319	-20.927 +/- 16.627	-22.496 +/- 7.959
**SASA energy**	-10.226 +/- 1.018	-19.471 +/- 1.233	-11.039 +/- 6.020	-12.481 +/- 1.198
**Binding energy**	-48.284 +/- 10.103	-83.579 +/- 18.11	-50.588 +/- 32.187	-60.521 +/- 12.439

**Fig 12:**
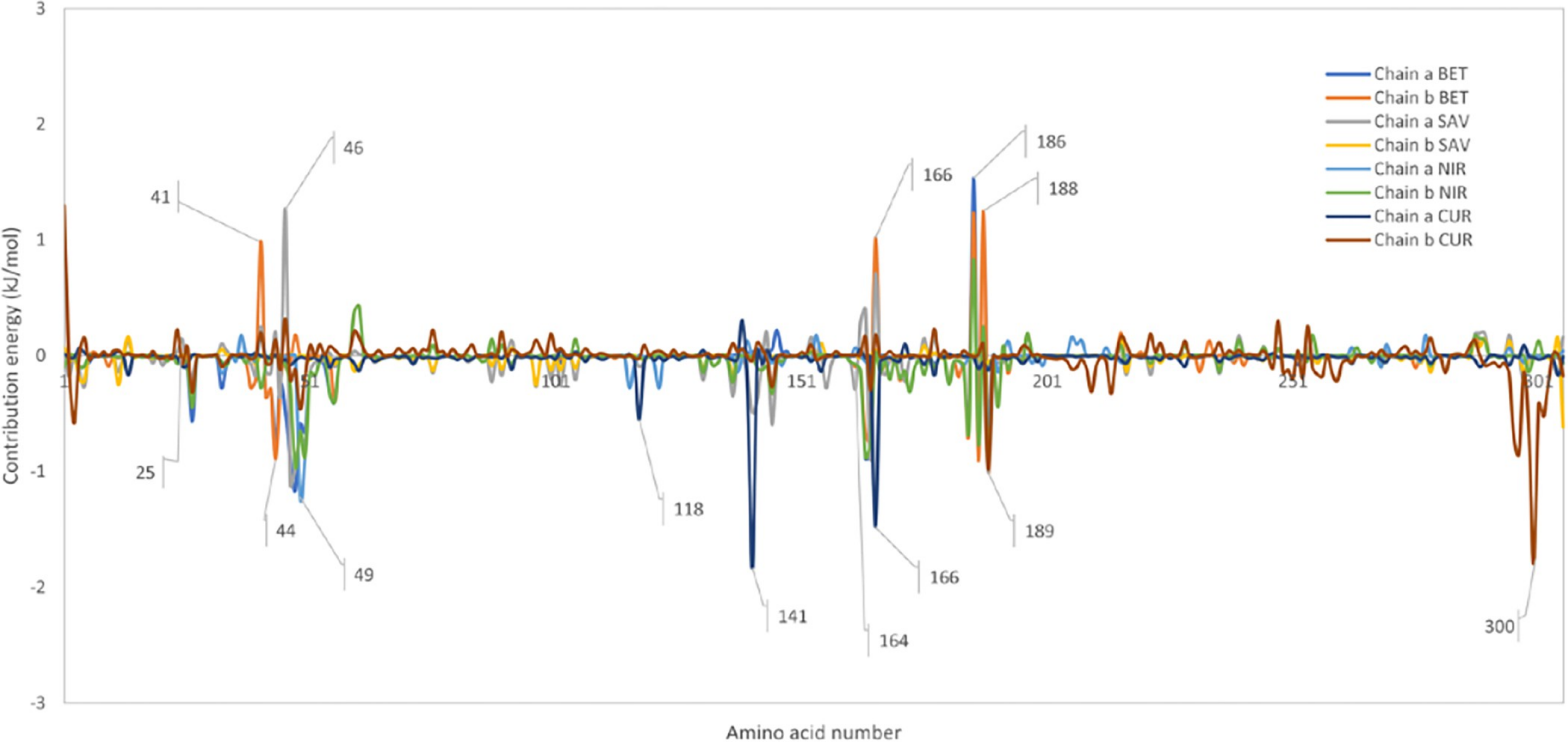
Contribution energy of residues from the ligand-bound SARS-CoV-2 M^pro^ complexes, in the dimeric state of the protein. The contribution energies of residues from SARS-CoV-2 M^pro^ in the betulinic acid-, Savinin-, Curcumin- and Nirmatrelvir-bound SARS-CoV-2 M^pro^ complexes are given. The positive values in kJ/mol indicates the negative contribution of the residue for ligand binding and the negative values represent the probable positive role of the residues in stabilisation of the ligand binding to the SARS-CoV-2 M^pro^.

In order to inspect the contribution of residues in binding to ligands, contribution energies were also calculated for each residue of ligand-bound SARS-CoV-2 M^pro^ compelxes, in the dimeric state of the protein (Fig 12). As can be observed from the results, in the Nirmatrelvir-SARS-CoV-2 M^pro^ complex, the following residues contributed positively to the energy of binding including His41, Val42, Gln142, His164 and Asp187. As for the curcumin-SARS-CoV-2 M^pro^ complex, Thr26, Gln142, Gly143 and Gly302 (from the other monomer) contributed to the binding. His41, had overall destabilised the binding of curcumin despite the hydrogen bond formed (Fig 11D). This suggests a low formation rate of this hydrogen bond and probably a more disrupting role of this residue in the binding pocket occupied by curcumin. Fig 12 further shows that the contribution energy calculated for betulinic acid-SARS-CoV-2 M^pro^ complex involved residues Thr25, His41, Gln189, Thr190, and Gln192 and ensured the stability of the betulinic acid during the MD simulation, while Val186 and Arg188 destabilised the ligand binding. Even though Glu166 led to higher stability of curcumin, in complex with betulinic acid, it destabilised the interaction. Similarly, Thr25, Thr26, Ser46, Gln142, Cys145, His164, Glu166 and Gln189 contributed to the energy of savinin binding to SARS-CoV-2 M^pro^.

### Buried surface area analysis

**Fig 13:**
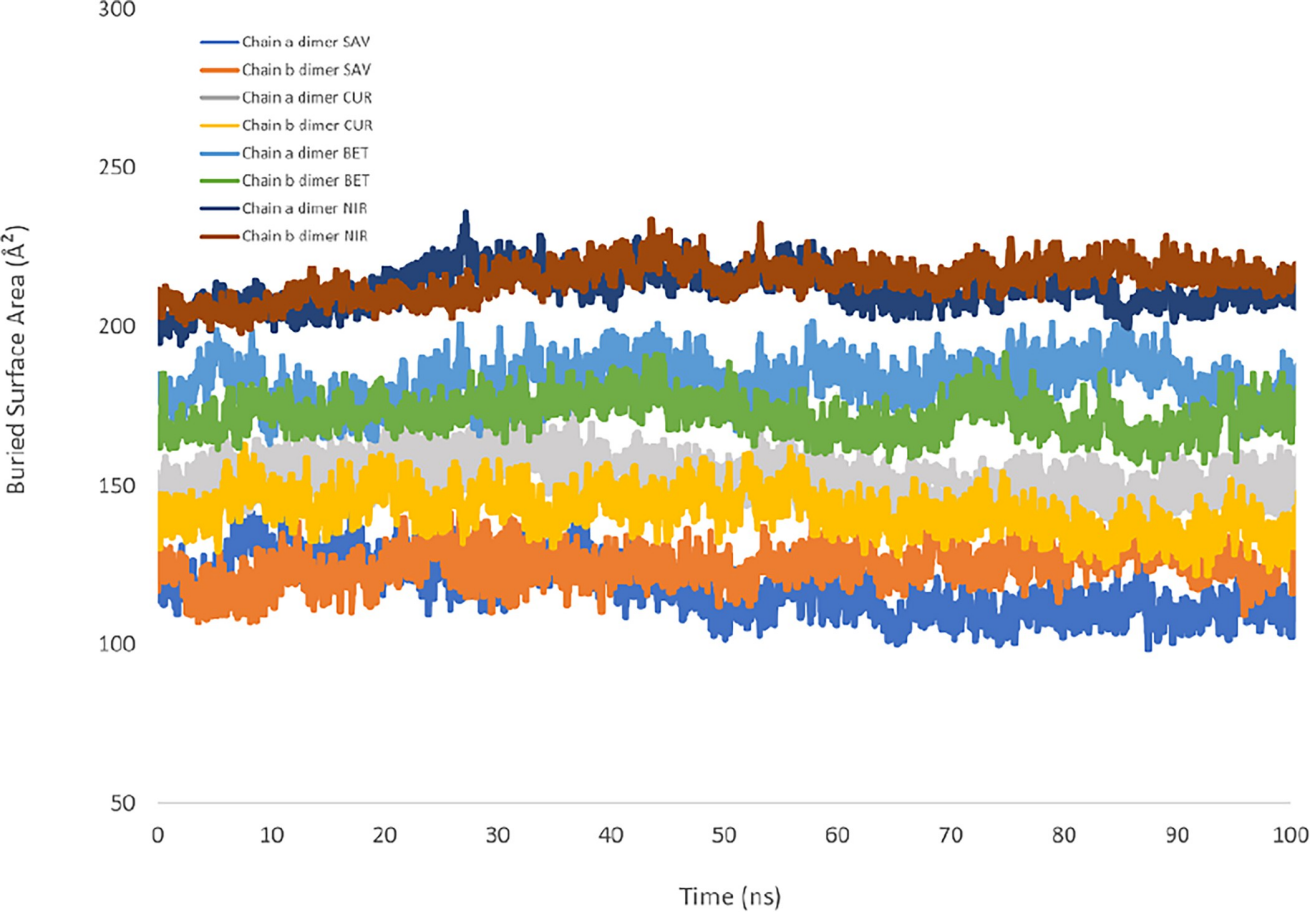
Buried Surface Area analysis of ligand-bound SARS-CoV-2 M^pro^ complexes, in the dimeric state of the protein. This figure compared the BSA value of each ligand in relation to the chain it was bound to. Nirmatrelvir (NIR) exhibited the highest BSA values indicating more area covered by this ligand. Savinin (SAV) presented the lowest values and hence higher access of solvent molecules to the binding pocket. Betulinic acid (BET) and curcumin (CUR) stood in middle, with betulinic acid covering more buried surface area than curcumin.

Buried Surface Area (BSA) analysis was performed based on a previous study [39]. The overlayed structures of the dimers (Fig 7) revealed that the positions of the monomers remained consistent relative to each other. Based on the consistency of conformation, for the calculation of SASA, each ligand and its corresponding monomer were selected for this calculation. As depicted in Fig 13, throughout the MD simulation, savinin followed by curcumin had the lowest BSA, allowing more solvent molecules to access the binding pocket. Conversely, Nirmatrelvir exhibited the highest BSA, indicating a greater occupation of the binding site, which is consistent with the bulky structure of this molecule. Despite the smaller structure of betulinic acid in comparison with other ligands, it covered the binding pocket more than savinin and curcumin, which might be due to the hydrophobic interactions in the binding pocket. Furthermore, the absence of abrupt changes in the BSA values suggested that the ligands occupied the binding pocket steadily, as demonstrated by the RMSD plot of each ligand in the dimeric form (Fig 6D).

## Conclusions

One of the essential proteins needed for the infection process and viral replication of SARS-CoV-2 is the 3C-like cysteine protease, also known as the Main protease (M^pro^), with a potential of being a key target for antiviral drug development with a conserved substrate binding site.

Using molecular docking it was possible to evaluate the binding affinities of 38 phytochemicals with SARS-CoV-2 M^pro^, in both the monomeric and dimeric states of the protein (as deposited asymmetric states of SARS-CoV-2 M^pro^ in the protein data bank). Three top scoring phytochemicals in terms of binding affinity, were identified to bind SARS-CoV-2 M^pro^ in the monomeric and dimeric states of the protein, respectively, including savinin from the *Acanthopanax henryi* plant (-8.864 and -8.518 Kcal/mol), betulinic acid from birch tree (-8.644 and -8.548 Kcal/mol) and curcumin from turmeric (-8.373 and -8.554 Kcal/mol). Savinin, betulinic acid and curcumin were reported to have therapeutic properties with antioxidant properties and hence hot temperaments [20], as well as antiviral effects [33,34]. To make the results more meaningful, the only approved inhibitor Nirmatrelvir [27] was used for comparison. Molecular docking was performed on Nirmatrelvir with SARS-CoV-2 M^pro^, in both the monomeric and dimeric states of the protein with the following binding affinities of -7.798 and -8.404 kcal/mol, respectively. ADMET analysis was conducted on all four ligands, showing that curcumin followed by savinin and betulinic acid, could satisfy the calculated rules as drugs (in terms of favourable criteria), compared to Nirmatrelvir.

In order to validate the molecular docking results, MD simulation analyses including calculations of RMSD, RMSF, Rg and number of hydrogen bonds, MM-PBSA, and BSA analysis, were performed on apo and savinin-, betulinic acid-, curcumin-, and Nirmatrelvir-bound SARS-CoV-2 M^pro^, in both the monomeric and dimeric states of the protein. The molecular behaviour and conformational dynamic differences between the monomeric and dimeric states of SARS-CoV-2 M^pro^, were assessed. The results showed that the active site residues interacted with the ligands via hydrogen bonding. For example, savinin, curcumin and Nirmatrelvir formed hydrogen bonds with Gln189. Cys145 also hydrogen bonded with curcumin and savinin. It should be noted here, that while Cys145 is commonly seen to be associated in covalent interactions with inhibitors, potent non-covalent inhibitors such as ML188 also exist [35], where Cys145 is non-covalently involved through either hydrogen bonding or hydrophobic interactions giving an even higher affinity of binding as assessed by molecular docking in this study (-8.518 Kcal/mol).

Moreover, His41 interacted with betulinic acid, curcumin and Nirmatrelvir via hydrogen bonds. Out of the ligands analysed, only curcumin formed hydrogen bonding with a residues from the neighbouring monomer (Ser301), which may emphasise on the effect of certain ligands in the stabilisation of the dimeric/active state of SARS-CoV-2 M^pro^. The MM-PBSA tool was used to calculate the binding energies and found that betulinic acid had the highest affinity for SARS-CoV-2 M^pro^ active site. Nirmatrelvir also had a high affinity but lower than betulinic acid. The analysis of the contribution energies of the residues for binding to the ligands revealed that Gln189 and His164 had positive effects. Buried surface area (BSA) analysis evaluated the ligand coverage in the binding pocket and showed that betulinic acid had a lower coverage than Nirmatrelvir, meaning that more water molecules could access the binding pocket. It was also concluded that hydrophobic non-polar interactions played the main role in ligand binding.

Overall, the findings from this study seem important, since the use of traditional medicine is advantageous over synthetic drugs, which can help overcome the toxicity and side effects of synthetic drugs. It is therefore highly recommended that experimental data for the binding of betulinic acid, savinin and curcumin bound to SARS-CoV-2 M^pro^ should be pursued. The study of phytochemicals against SARS-CoV-2 M^pro^ is most important at the time where there is still an ongoing number of infected cases and deaths reported due to increasing rate of mutations in the spike protein of SARS-CoV-2 [40], which renders vaccination less effective [41]. Additionally, the concept of temperament is highly important and can affect the severity of a disease as reported [26].

## Methods

### Bioinformatics analyses

Initially the fasta format of the SARS-CoV-2 M^pro^ sequence was taken and a protein blast search was performed in ncbi against all known coronavirus M^pro^ PDB structures. Subsequently, the outcome of the search was assessed and the non-mutated, native M^pro^ structures with bound inhibitors were selected and assessed by multiple sequence alignment. The ligand binding mode (whether bound via hydrogen bonding or hydrophobic interactions) in each structure was then analysed by Ligplot [42] and the common residues of coronavirus M^pro^ involved in binding to the ligands assessed.

### Compound selection

The selection of compounds from plants/herbs/trees in this study were based on almost 300 references (please see supplementary data, S5 and S6 Tables in S1 File), with emphasis on the therapeutic and in particular antioxidant and antiviral properties of the active constituents.

### Molecular docking

The molecular docking program ‘DockThor’ [43–45] was used to assess the binding of herbal compounds/phytochemicals, considering the initial results of docking five synthetic inhibitors, including N3, α-ketoamide inhibitor, GC-376, and peptidomimetic inhibitors 11a and 11b [10,11,46–51], from experimentally solved structures of SARS-CoV-2 M^pro^ in complex with the inhibitors. This was done in order to better understand how the DockThor program worked and to gain confidence in the results. In continuation, the docking program was used and the best solutions (with RMSD of 0.0) from the DockThor outputs were used and analysed by Ligplot and Chimera [52]. The emphasis on the DockThor output and the ranking of the compounds was based on affinity prediction values (Kcal/mol) of different ligands in virtual screening experiments considering the top-energy pose (according to Total Energy) of each compound. User defined docking in DockThor included grid centre values of 12, 0, 24 for axes x, y and z, respectively, for SARS-CoV-2 M^pro^ in the monomeric state (pdb ID 6yb7) and -10, -10, 40 for axes x, y, z, respectively, in the dimeric state (pdb ID 7ali). These regions covered the substrate binding area in SARS-CoV-2 M^pro^ (deposited either in the monomeric/dimeric states) as identified via superposition of previously ligand-bound structures of coronavirus M^pro^. Default settings were used for the DockThor runs. Additionally, six drugs used for COVID-19 including Remdesivir, Darunavir, Oseltamivir, Lopinavir, Ritonavir and Ribavirin; a non-covalent inhibitor ML188; and an approved covalent inhibitor Nirmatrelvir, were all docked with SARS-CoV-2 M^pro^, using the same methods described here and results tabulated.

### Re-docking

Validation of the docking protocol was performed for the savinin-, betulinic acid- and curcumin-bound SARS-CoV-2 M^pro^ complexes in DockThor [43–45]. Initially the ligands were omitted from the files containing the complex information and used as the ‘protein file’. The best ranking ligand pose with RMSD of 0.0 obtained in the initial docking was then used as the ‘ligand’ and re-docking performed. The final result containing the best ranking ligand pose was compared with the reference conformation (the pose of the ligand upon initial docking), leading to the calculated RMSD values.

### ADMET calculations

The ADMET calculations were performed using the ADMETlab2.0 [36].

### MD simulation

The top scoring savinin-, betulinic acid-, curcumin- and Nirmatrelvir-bound SARS-CoV-2 M^pro^ complexes, as well as the apo structure of SARS-CoV-2 M^pro^, were subjected to MD simulation (in both the monomeric and dimeric states), using GROMACS 2019.1 simulation package [53]. The MD simulations were conducted using CHARMM36 forcefield [54]. Structural parameters for savinin, betulinic acid, curcumin and Nirmatrelvir, based on CHARMM36 force field, were obtained from CGenFF webserver [55,56]. Structures were solvated with TIP3P water model [57] in a cubic box, a 1 nm distance between protein and box edges. The system was neutralized by adding sufficient counter charge ions. Energy minimisation of the system was performed by steepest descent integrator [58]. Subsequently, the simulations were performed with Berendsen [59] and Parrinello-Rahman [60] coupling algorithms and following SHAKE integrator [61]. The simulation results from the GROMACS software were analysed as RMSD, RMSF, Rg and number of Hydrogen bonds. Occupancy of the hydrogen bonds, during the MD simulations, were calculated from the number of hydrogen bonds formed between the SARS-CoV-2 M^pro^ and the ligand in each frame using Microsoft Excel (available at https://office.microsoft.com/excel.). UCSF Chimera was used for the visualisation of the hydrogen bonds and generation of figures. For MD simulation of either the apo or ligand-bound SARS-CoV-2 M^pro^ structures, molecular docking results using PDB IDs 6yb7 and 7ali for monomeric and dimeric states, respectively, were used.

### Binding free energies and residue contributions

Binding free energies (ΔG_Bind_) of protein-ligand (or protein-DNA and protein-protein) complexes in a solvent can be calculated from theMM-PBSA. Binding free energies and residue contributions for savinin-,betulinic acid-, curcumin- and Nirmatrelvir-SARS-CoV-2 M^pro^ complexes were calculated using the g_mmpbsa package [38]. The MM-PBSA calculation was performed based on the protocol available at https://rashmikumari.github.io/g_mmpbsa.

### BSA analysis

BSA analysis was performed based on a previous study [37]. The SASA for each ligand was computed using Visual Molecular Dynamics (VMD) [62] and a TCL script, following the Richards and Lee method with a water probe size of 1.4 Å [63]. Subsequently, the BSA was calculated using the formula BSA = 0.5*(SASA ligand + SASA protein–SASA complex).

## Supporting information

S1 File S1-S3 Figs and S1-S12 Tables are provided in the file entitled “Supporting Information”. The Figure legends and Table titles are also included in the “Supporting Information” file.
(PDF)
