# Supplementary material for: In silico studies of anti-oxidative and hot temperament-based phytochemicals as natural inhibitors of SARS-CoV-2 Mpro
Source: PLoS One. 2023 Nov 30;18(11):e0295014. doi: 10.1371/journal.pone.0295014 (PMC10688677; doi:10.1371/journal.pone.0295014)
Supplement: S1 File — The Figure legends and Table titles are also included in the “Supporting Information” file. (PDF) [file pone.0295014.s001.pdf]

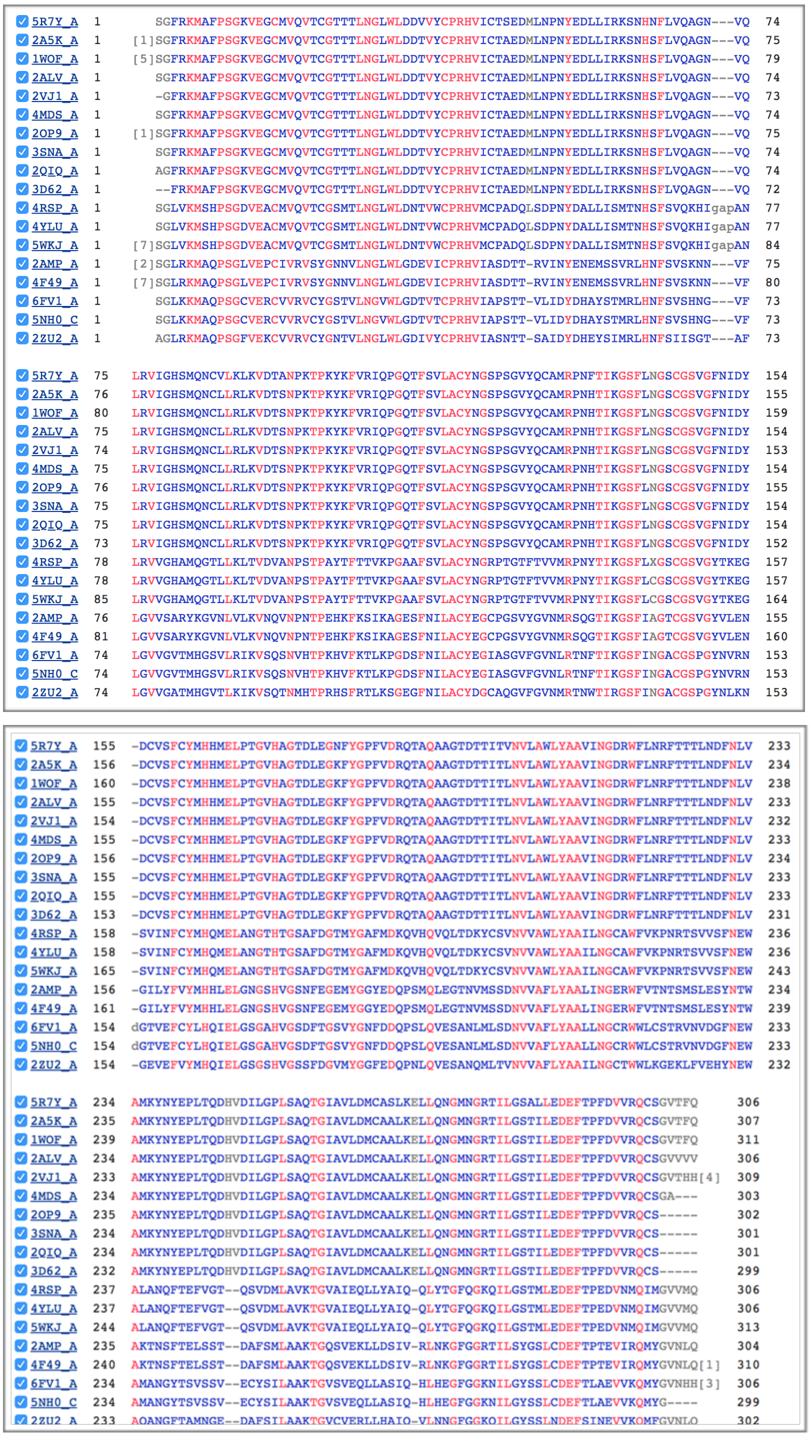

Figure S1. Sequence alignment of various coronavirus Mpro **sequences using SARS-CoV-2 sequence as the query sequence.** The search was for pdb entries in ncbi using protein blast. The residues in red are conserved residues.

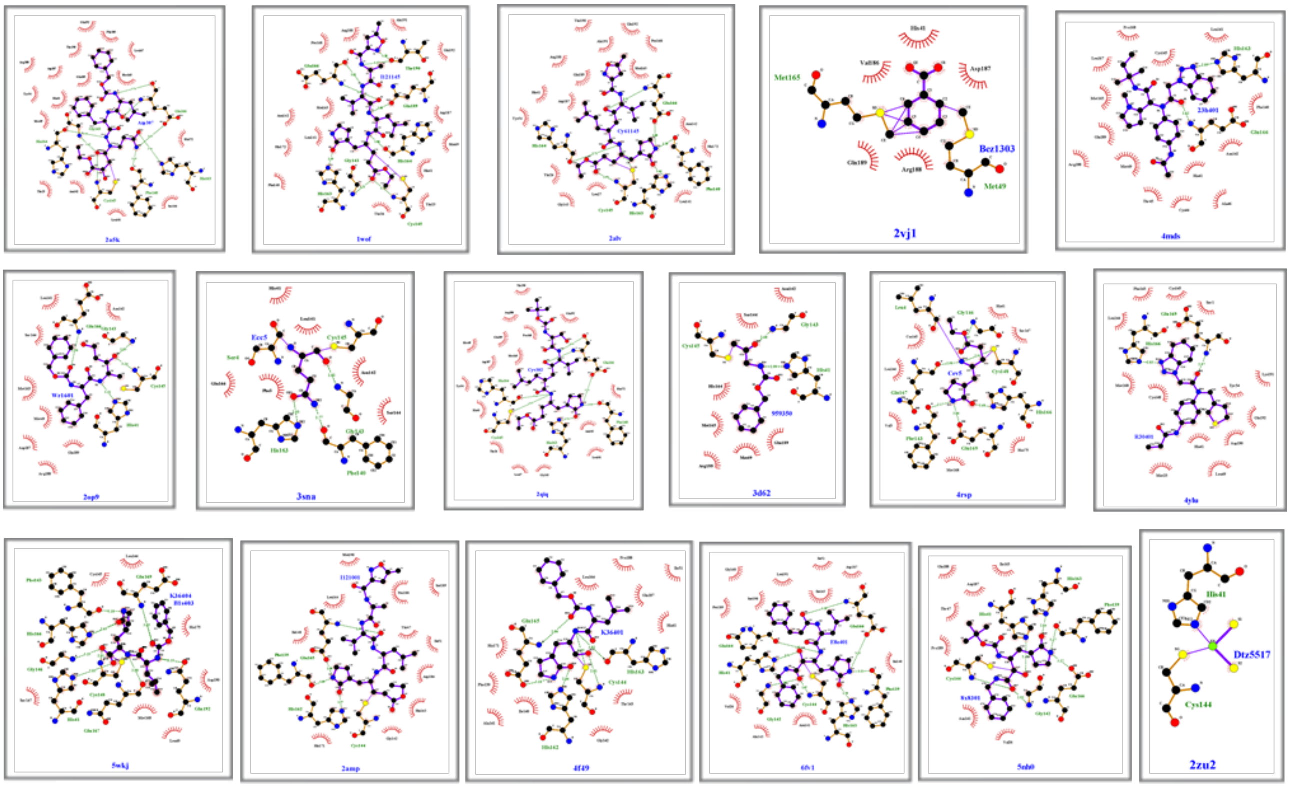

Figure S2. Ligplot analysis of various Coronavirus M^pro structures in complex with a ligand/ inhibitor to identify functional residues involved in ligand binding and highlight the structural conservations in complex formation. The figure was generated using Ligplot.

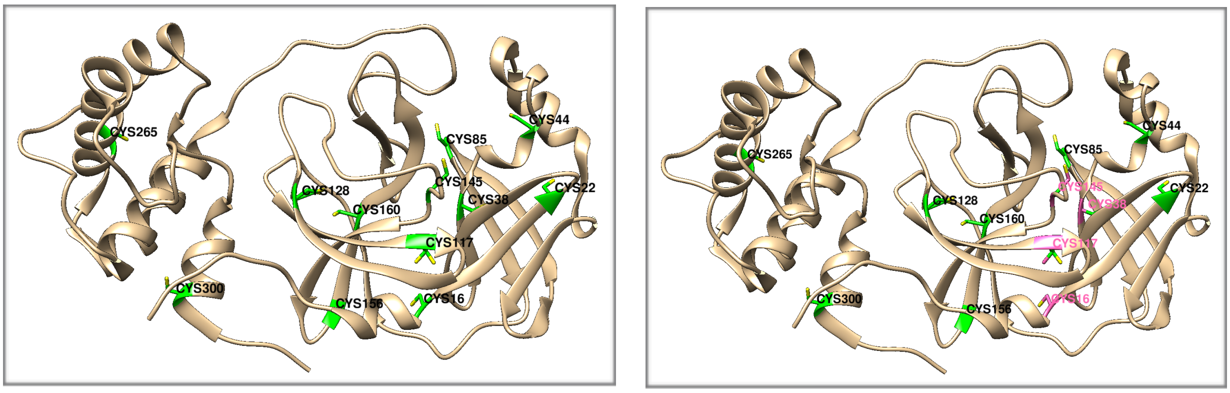

(a) **(b)**

Figure S3. Ribbon representation of the structure of SARS-CoV-2 M^{pro} in the monomeric form. (a) The overall number of cysteines are shown in green. (b) Conserved cysteines are shown in pink along with the non-conserved cysteines in green. The figure was generated using Chimera.

Table S1. Analysis of binding interactions between the ligands and the M^{pro} of various coronoviruses. The percentage sequence identity of each M^{pro} related to SARS-CoV-2 M^{pro} are given. The ligand binding analysis was performed using Ligplot and the sequence of SARS-CoV-2 M^{pro} was used as the query.

PDB ID and origin of main protease	Residues involved in hydrogen bonding (Å)	Residue involved in covalent bonding	Residues involved in hydrophobic interactions	Sequence identity in percentage relative to SARS-CoV-2 main protease
**2a5k**<br>**SARS-related**	1. Phe140-O:Ligand-NDE (3.25)<br>2. Gly143-N:Ligand-OBI (2.67)<br>3. Cys145-N:Ligand-OBI (3.19)<br>4. His163-NE2:Ligand-OBD (2.75)<br>5. His164-O:Ligand-NAX (2.94)<br>6. Glu166-N:Ligand-O (2.59)<br>7. Glu166-O:ligand-N (2.73)<br>8. Glu166-OE2:Ligand-NBE (3.28)	Cys145-SG:Ligand-CBM	Thr25; His41; Met49; Tyr54; Leu141; Asn142; Ser144; Met165; Leu167; His172; Phe185; Asp187; Arg188; Gln189; Thr190; Gln192	96.08
**1wof**<br>**SARS-related**	1. Gly143-N:Ligand-O6 (3.21)<br>2. Cys145-N:Ligand-O6 (3.10)<br>3. His163-NE2:Ligand-O8 (2.59)<br>4. His164-O:Ligand-N5 (2.86)<br>5. Glu166-N:Ligand-O4 (2.88)<br>6. Glu166-O:Ligand-N3 (3.01)<br>7. Gln189-OE1:Ligand-N4 (3.00)	Cys145-SG:Ligand-C21	Thr25; Thr26; His41; Met49; Phe140; Leu141; Asn142; Met165; Pro168; His172; Asp187; Arg188 Ala191; Gln192	96.08
**2alv**<br>**SARS-related**	1. Phe140-O:ligand-N44 (3.28)<br>2. Cys145-SG:Ligand-N31 (2.76)<br>3. Cys145-N:Ligand-O36 (2.93)<br>4. His163-NE2:Ligand-O46 (2.64)<br>5. His164-O:Ligand-N31 (2.71)<br>6. Glu166-OE2:Ligand-N44 (3.08)<br>7. Glu166-N:ligand-O19 (3.11)	Cys145-SG:Ligand-C33	Thr26; Leu27; His41; Tyr54; Leu141; Asn142 Gly143; Met165; Pro168; His172; Asp187; Arg188; Gln189; Thr190; Ala191; Gln192	96.04

PDB ID and origin of main protease	Residues involved in hydrogen bonding (Å)	Residue involved in covalent bonding	Residues involved in hydrophobic interactions	Sequence identity in percentage relative to SARS-CoV-2 main protease
**2vj1<br>SARS-related<br>(two compounds bound)**	1st compound<br>Cys145-N:Ligand-OAH (3.15)<br><br>2nd compound<br>-	Cys145-SG:Ligand-CAC<br><br>1. Met49-CE:Ligand-C2<br>2. Met165-SD:Ligand-C6<br>3. Met165-SD:Ligand-C5<br>4. Met165-CE:Ligand-C4<br>5. Met165-CE:Ligand-C5<br>6. Met165-CE:Ligand-C6	Leu141; Asn142; Gly143; His163; Glu166<br><br>His41; Val186; Asp187; Arg188; Gln189	96.04
**4mds<br>SARS-related**	1. His163-NE2:Ligand-N5 (2.89)<br>2. Glu166-N:Ligand-O1 (2.82)	-	His41; Cys44; Thr45; Ala46; Met49; Phe140; Leu141; Asn142; Cys145; Met165; Leu167; Pro168; Arg188; Gln189	95.71
**2op9<br>SARS-related**	1. His41-NE2:Ligand-N1 (3.31)<br>2. Gly143-N:Ligand-O2 (3.05)<br>3. Cys145-N:Ligand-O2 (3.24)<br>4. Glu166-N:Ligand-O4 (2.96)	-	Met49; Leu141; Asn142; Ser144; Met165; Asp187; Arg188; Gln189	96.01
**3sna<br>SARS-related**	1. Gly143-N:Ligand-O (2.82)<br>2. Phe140-O:Ligand-NE2 (2.77)<br>3. His163-NE2:Ligand-OE1 (2.93)	1. Ser4-C:Ligand-N<br>2. Cys145-SG:Ligand-C	Phe3; His41; Leu141; Asn142; Ser144; Glu166	96.01
**2qiq<br>SARS-related**	1. Phe140-O:Ligand-N16 (3.00)<br>2. Cys145-SG:Ligand-N20 (3.17)<br>3. His163-NE2:Ligand-O18 (2.72)<br>4. His164-O:Ligand-N20 (3.14)<br>5. Glu166-OE2:Ligand-N16 (2.72)<br>6. Glu66-O:Ligand-N36 (3.02)<br>7. Glu166-N:Ligand-O31 (3.07)	-	Thr26; Leu27; His41; Met49; Tyr54; Leu141; Asn142; Gly143; Met165; Pro168; His172; Asp187; Arg188; Gln189; Thr190; Gln192	95.68
**3d62<br>SARS-related**	1. His41-NE2:Ligand-N10 (2.99)<br>2. Gly143-N:Ligand-O15 (2.68)	Cys145-SG:Ligand-C16	Met49; Asn142; Ser144; His164; Met165; Arg188; Gln189	95.99
**4rsp<br>MERS-related**	1. Phe143-O:Ligand-N69 (3.17)<br>2. Gly146-N:Ligand-O88 (3.05)<br>3. Cys148-SG:Ligand-N49 (2.91)<br>4. His166-NE2:Ligand-O66 (2.68)<br>5. Glu169-OE1:Ligand-N69 (3.06)	1. Leu4-C:Ligand-N49<br>2. Cys148-SG:Ligand-C16	Val3; His41; Leu144; Cso145; Ser147; Met168; His175	50.65

PDB ID and origin of main protease	Residues involved in hydrogen bonding (Å)	Residue involved in covalent bonding	Residues involved in hydrophobic interactions	Sequence identity in percentage relative to SARS-CoV-2 main protease
**4ylu MERS-related**	1. His166-NE2:Ligand-N24 (2.83)<br>2. Glu169-N:Ligand-O01 (2.87)	-	Ser1; Met25; His41; Leu49; Tyr54; Phe143; Leu144; Cys145; Cys148; Met168; Asp190; Lys191; Gln192	50.65
**5wkj MERS-related**	1. His41-NE2:Ligand-O22 (2.83)<br>2. Phe143-O:Ligand-N28 (3.10)<br>3. Gly143-N:Ligand-O22 (3.25)<br>4. Cys148-N:Ligand-O22 (2.81)<br>5. Cys148-SG:Ligand-N19 (2.88)<br>6. His166-NE2:Ligand-O30 (2.65)<br>7. Gln167-O:Ligand-N19 (2.90)<br>8. Gln192-OE1:Ligand-N11 (3.33)	Cys148-SG:Ligand-C21	Leu49; Leu144; Cys145; Ser147; Met168; His175; Asp190	50.65
**2amp gastroenteritis virus**	1. Phe139-O:Ligand-N6 (3.09)<br>2. His162-NE2:Ligand-O8 (2.57)<br>3. Glu165-OE1:Ligand-N6 (2.98)<br>4. Glu165-N:Ligand-O4 (2.99)	Cys144-SG:Ligand-C21	Thr47; Ile51; Ile140; Gly142; His163; Leu164; His171; Asp186; Pro188; Ser189; Met190	44.44
**4f49 gastroenteritis virus**	1. Cys144-N:Ligand-O22 (2.81)<br>2. Cys144-SG:Ligand-N19 (2.93)<br>3. His162-NE2:Ligand-O30 (2.86)<br>4. His163-O:Ligand-N19 (3.02)<br>5. Glu165-N:Ligand-O18 (3.06)	Cys144-SG:Ligand-C3	His41; Ile51; Phe139; Ile140; Ala141; Gly142; Thr143; Leu164; His171; Gln187; Pro188	44.44
**6fv1 Human coronavirus**	1. His41-NE2:Ligand-O58 (2.51)<br>2. Gly142-N:ligand-O67 (2.98)<br>3. Cys144-N:Ligand-O67 (2.87)<br>4. Phe139-O:Ligand-N49 (3.12)<br>5. His163-NE2:Ligand-O48 (2.78)<br>6. Glu166-N:Ligand-O01 (3.20)<br>7. Glu166-OE2:Ligand-N49 (3.23)<br>8. Gln164-O:Ligand-N38 (3.03)	Cys144-SG:Ligand-C57	Val26; Ile51; Ile140; Asn141; Ala143; Ile165; Gly168; Asp187; Pro189; Ser190; Leu191	44.41
**5nh0 Human coronavirus**	1. His41-NE2:Ligand-O58 (2.49)<br>2. Gly142-N:ligand-O67 (2.93)<br>3. Cys144-N:Ligand-O67 (2.91)<br>4. Phe139-O:Ligand-N49 (3.27)<br>5. His163-NE2:Ligand-O48 (2.84)<br>6. Glu166-OE1:Ligand-N49 (3.27)	Cys144-SG:Ligand-C57	Val26; Thr47; Asn141; Ile165; Asp187; Gln188; Pro189	44.55

PDB ID and origin of main protease	Residues involved in hydrogen bonding (Å)	Residue involved in covalent bonding	Residues involved in hydrophobic interactions	Sequence identity in percentage relative to SARS-CoV-2 main protease
**2zu2 Human coronavirus**	-	1. His41-NE2:Ligand-Zn<br>2. Cys144-SG:Ligand-Zn	-	41.04

Table S2. Description of experimentally derived binding interactions of five inhibitors associated with SARS-CoV-2 M^{pro}, their IC_{50} and EC_{50} values and chemical structures. The PDB IDs included 6lu7, 6y2f, 6wtt, 6m0k and 6lze. The compounds are listed in the order of their EC_{50} values, highest (least potent) to lowest (most potent).

Inhibitors	PDB ID	(i): IC_{50} (ii): EC_{50} (μM)	Residues involved in binding			Structure	Ref
			Hydrophobic bonds	Hydrogen bonds (Å)	Covalent bonds		
**N3**	6LU7	(i): Not available (ii): 16.77 ± 1.70	Thr24, Thr25, Thr26, His41, Leu141, Asn142, Met165, Pro168, His172, Ala191, Gln192	Phe140-O:Ligand-N6 (3.13) Gly143-N:Ligand-O (2.80) Cys145-SG:Ligand- N5 (3.15) His163-NE2:Ligand- O8 (2.52) Glu166-O:Ligand-N (2.83) Glu166-N:Ligand-O (2.98) Gln189-OE1:Ligand- N (2.93) Thr190-O:Ligand-N (2.85)	Cys145-SG:Ligand-C20	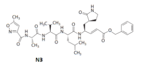	(1-5)
**α-ketoamide inhibitor (13b)**	6Y2F	(i): 0.67 ± 0.18 (ii) 4–5	Thr26, Met49, Leu141, Asn142, Met165, Leu167, Pro168, His172, Asp187, Gln189	Phe140-O:Ligand-N49 (3.20) Gly143-N:Ligand-O41 (2.76) Cys145-N:Ligand-O41 (3.03) His163-NE2:Ligand- O48 (2.57) His164-O:Ligand-N38 (2.83) Glu166-O:Ligand-N23 (2.96) Glu166-N:Ligand-O22 (2.89)	Cys145-SG:Ligand-C57	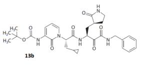	(6)
**GC-376**	6WTT	(i): 0.030 ± 0.008 (ii): 3.14 ± 1.01	His41, Met49, Leu141, Asn142, Gly143, Met165, Pro168, His172, Asp187, Thr190, Ala191, Gln192	Phe140-O:Ligand-N28 (3.19) Cys145-SG:Ligand-N19 (2.90) Cys145-N:Ligand-O22 (2.93) His163-NE2:Ligand- O30 (2.66) His164-O:Ligand-N19 (2.86) Glu166-OE1:Ligand-N28 (3.33) Glu166-N:Ligand-O10 (3.00) Gln189-OE1:Ligand-N11 (3.02)	Cys145-SG:Ligand-C21	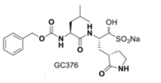	(7)

**peptidomimetic inhibitor (11b)**	6M0K	(i): 0.040 ± 0.002<br>(ii): 0.72 ± 0.09	His41, Met49, Leu141, Asn142, Ser144, Met165, His172, Val186, Asp187,Arg188	Phe140-O:Ligand-N29 (3.26)<br>Gly143-N:Ligand-O33 (3.28)<br>Cys145-SG:Ligand-N23 (2.86)<br>Cys145-N:Ligand-O33 (2.83)<br>His163-NE2:Ligand- O31 (2.71)<br>His164-O:Ligand-N23 (3.29)<br>Glu166-O:Ligand-N11 (2.57)<br>Glu166-N:Ligand-O01 (2.81)<br>Glu166-OE2:Ligand-N29 (2.96)	Cys145-SG:Ligand-C32	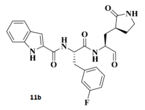	(8)
**peptidomimetic inhibitor (11a)**	6LZE	(i): 0.053 ± 0.005<br>(ii): 0.53 ± 0.01	His41, Met49, Tyr54, Leu141, Asn142, Ser144, Met165, His172, Asp187, Arg188, Gln189	Phe140-O:Ligand-N28 (3.19)<br>Gly143-N:Ligand-O32 (3.32)<br>Cys145-SG:Ligand-N22 (2.89)<br>Cys145-N:Ligand-O32 (2.88)<br>His163-NE2:Ligand- O30 (2.67)<br>His164-O:Ligand-N22 (3.23)<br>Glu166-O:Ligand-N11 (2.64)<br>Glu166-N:Ligand-O01 (2.90)<br>Glu166-OE2:Ligand-N28 (3.04)	Cys145-SG:Ligand-C31	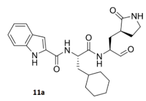	(8)

Table S3. Comparison between molecular docking results using DockThor and experimentally derived results of five SARS-CoV-2 M^{pro} structures with different ligands/ inhibitors bound. The PDB IDs included 6lu7, 6y2f, 6wtt, 6m0k and 6lze. Common residues also found in binding interactions in the experimentally derived M^{pro}-ligand complex structures are shown in bold. The inhibitors are listed based on their score of binding affinity, from highest to lowest.

**Inhibitors**	**Residues involved in hydrogen bonding (Å)**	**Residues involved in hydrophobic interactions**	**Number of amino acids involved in hydrophobic interactions**	**Dockthor**<br>**a) Affinity (Kcal/mol)**<br>**b) Total energy**<br>**c) Van der Waals Energy**<br>**d) Electrostatic energy**
**N3**	1. **Thr190**-O:ligand-N5 (2.90)	**Thr24, Thr25,** Ser46, Met49, **Cys145, Met165, Glu166, Pro168, Gln189**	9	a) -8.636<br>b) 29.291<br>c) -32.035<br>d) -5.250
**alpha-ketoamide inhibitor (13b)**	**1. Glu166**-OE1:Ligand- O6 (2.58)<br>**2. Asn142**-ND2:Ligand-O3 (2.82)	Thr25, Ser46, **Met49**, **Met165**, **Pro168**, **Gln189**	6	a) -8.330<br>b) 30.718<br>c) -21.871<br>d) -13.353
**GC-376**	1. Thr26-O:ligand- N3 (2.74)<br>2. **His41**-ND1:ligand-O4 (3.27)<br>3. **His41**-O:ligand- O4 (3.34)<br>4. **Gln189**-OE1:Ligand-N1 (2.91)	Thr25, Leu27, Cys44, Ser46, **Met49, Gly143, Cys145**, **Met165, Glu166,** Leu167, Arg188	11	a) -8.313<br>b) -19.099<br>c) -25.918<br>d) -8.866
**peptidomimetic inhibitor (11b)**	**1. Gly143**-N:Ligand-O4 (2.76)<br>**2.** Gln189-OE1:Ligand- N1 (2.78)	Thr25, **His41,** Cys44, Ser46, **Ans142, Cys145, Glu166**	7	a) -8.082<br>b) 5.558<br>c) -23.135<br>d) -14.946
**Peptidomimetic inhibitor (11a)**	**1. Asn142**-ND2:ligand- O1 (3.35)<br>**2. Gly143**-N:ligand-O3 (2.81)<br>**3. His163**-NE2:ligand-O4 (2.43)<br>**4. Glu166**-N:Ligand-O[illegible] (3.28)	**His41,** Ser46, **Phe140, Cys145, Met165,** Pro168, **Gln189**	7	a) -7.620<br>b) -1.292<br>c) -17.567<br>d) -20.598

Table S4. Molecular docking results of six drugs used against SARS-CoV-2 M^{pro} including Remdesivir, Darunavir, Oseltamivir, Lopinavir, Ritonavir and Ribavirin. The drugs are listed based on their binding affinities from highest to lowest.

Drugs used for COVID-19	Residues involved in hydrogen bonding (distance in Å)	Residues involved in hydrophobic interactions	Number of amino acids involved in hydrophobic interactions	DockThor<br>a) Affinity (Kcal/mol)<br>b) Total energy<br>c) Van der Waals Energy<br>d) Electrostatic energy	2D Chemical Structures from pubchem
**Remdesevir**	Glu166-OE1:Ligand-O2 (2.64)	His41; Cys44; Phe140; Leu141; Asn142; Cys145; His163; Pro168; Gln189	9	8. -8.894<br>9. 12.089<br>8. -30.276<br>8. -13.325	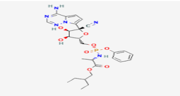
**Darunavir**	Asn142-ND2:Ligand-O6 (2.81)	Thr25; His41; Ser46; Met49; Glu166; Gln189	6	7. -8.399<br>8. 25.635<br>9. -25.220<br>10. -5.963	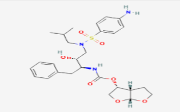
**Oseltamivir**	Asn142-OD1:Ligand-N1 (2.65)<br><br>Glu166-OE1:Ligand-N2 (2.45)	His41; Met49; Leu141; Cys145; Met165; Gln189; Thr190	7	3. -7.958<br>4. -3.116<br>5. -12.327<br>6. -29.734	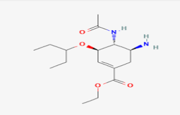
**Lopinavir**	Phe140-O:Ligand-N (3.00)<br><br>His163-NE2:Ligand-O1 (2.64)	Leu141; Asn142; Gly143; Cys145; Met165; Glu166	6	5. -7.366<br>6. 571.554<br>7. -14.379<br>8. -9.389	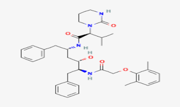
**Ritonavir**	-	His41; Cys44; Met49	3	4. -7.099<br>5. 449.135<br>6. -15.151<br>3. -7.533	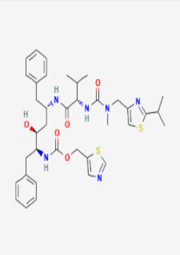
**Ribavirin**	Phe140-O:Ligand-N4 (2.86)<br><br>Asn142-OD1:Ligand-O2 (2.77)<br><br>Asn142-ND2:Ligand-O2 (3.27)<br><br>His163-NE2:Ligand-O5 (2.46)<br><br>Gln189-OE1:Ligand-O4 (2.73)	Met49; Met165; Glu166	3	8. -6.705<br>9. 18.402<br>10. -7.955<br>11. -22.399	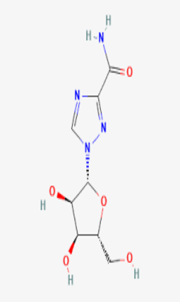

Table S5. List of herbs/plants/trees used in this study including their therapeutic properties and antiviral effects.

Herbs/plants/ trees (family)	Therapeutics properties	Antiviral effects
Ginger (*Zingiberaceae*)	antioxidant (9-11), antiplatelet (12), anti-Inflammatory (13), antibacterial (14), antitumor (15), antifungal (16), antihyperlipidemic (17), antispasmodic (18), antihypertensive (19), anti-allergic (20), antidiabetic (21), anti-obesity (22), anti-diarrheal (23), anxiolytic (24), antiemetic (24), immunosuppressive (25), cardioprotective (26), radioprotective (27)	herpes simplex virus type 1 (28), herpes simplex virus type 2 (29), human respiratory syncytial virus (HRSV) (30), avian influenza virus H9N2 (31), hepatitis C virus (32), rhinovirus (33), chikungunya virus (34), feline calicivirus (FCV) (35), caprine alphaherpesvirus 1 (CpHV-1) (36)
Turmeric or Curcuma longa L. (*Zingiberaceae*)	antioxidant (9, 37), anti-inflammatory (38), antibacterial (39), anti-proliferative (40), antiprotozoal (41), antifungal (42), antidiabetic (43), anti-obesity (44), antidepressant (45), anticonvulsant (46), immunomodulatory (40), hepatopreventive (47)	herpes simplex virus type 1 (HSV-1) (48), human immunodeficiency virus (HIV) (49), influenza A virus (IAV) (50), Hepatitis B virus (HBV) (51), Hepatitis C virus (HCV) (52), dengue virus (DENV) (53)
Elderberry or *Sambucus nigra L.* *(Adoxaceae)*	antioxidant (54-56), anti-inflammatory (57), antibacterial (58), anti-cancer (59), antidiabetic (60), antidepressant (61), anticonvulsant (62), immune-enhancing (63)	infectious bronchitis virus (IBV) (64), herpes simplex virus type 1 (65), influenza A virus (58, 65-68), influenza B virus (58, 65, 67, 68), HIV-1 virus (69), feline immunodeficiency virus (FIV) (70)
Cinnamon *(Lauraceae)*	antioxidant (71-73), anti-inflammatory (74), antibacterial (75), anti-cancer (76), antifungal (77), antihypertension (78), anti-allergic (79), antidiabetic (80), antiulcerogenic (81), antipyretic (82)	HIV-1 (83), HIV-2 (83), influenza A/ PR/8 virus (84)
Garlic or *Allium sativum L.* *(Liliaceae)*	antioxidant (85-87), anti-inflammatory (88), antibacterial (89), anti-cancer (90), antiparasitic (91), antifungal (92), antihypertensive (93), anti-atherosclerotic (94), antidiabetic (95), anti-obesity (96), immunomodulatory (97)	herpes simplex virus type 1 (98), herpes simplex virus type 2 (99), human cytomegalovirus (HCMV) (100), HIV (101), avian influenza virus H9N2 (31), influenza B virus (98)

Onion or *Allium cepa L. (Liliaceae)*	antioxidant (102-104), antiplatelet (105), anti-inflammatory (106), antibacterial (103), anti-cancer (107), antiparasitic (108), antifungal (109), antidiabetic (110), anti-obesity (96)	Herpes Simplex Virus type-1 (HSV-1) (111), human immunodeficiency virus (HIV) (112), Adenovirus (113), Newcastle Disease Virus (NDV) (114)
Black cumin or *Nigella sativa L. (Ranunculaceae)*	antioxidant (115, 116), anti-inflammatory (117), antibacterial (118), anti-cancer (119), antiparasitic (120), antifungal (121), antiasthmatic (122), antidiabetic (123), anti-obesity (124), anti-anxiety (125), anti-arthritic (126), anticonvulsant (127), analgesic (128), cardioprotective (129), gastroprotective (130), hepatoprotective (131), immunopotentiating (132)	murine cytomegalovirus (MCMV) (133), Epstein–Barr virus (EBV) (134), human immunodeficiency virus (HIV) (135), avian influenza virus H9N2 (136), Influenza virus H5N1 (137), hepatitis C virus (HCV) (138, 139), Zucchini yellow mosaic virus (ZYMV) (140)
Peppermint or *Mentha piperita L. (Lamiaceae)*	antioxidant (141-143), anti-inflammatory (144), antibacterial (143), anti-cancer (145), anthelmintic (146), antifungal (147), antispasmodic (148), anti-allergic (149), antidiabetic (150), antinociceptive (151), antitussive (152)	respiratory syncytial virus (RSV) (153), herpes simplex virus type 1 (HSV-1) (154), herpes simplex virus type 2 (HSV-2) (154), human immunodeficiency virus-1 (HIV-1) (155)
Broccoli or *Brassica oleracea L. var. italica (Brassicaceae)*	antioxidant (156-158), anti-inflammatory (159), antibacterial (160), anti-cancer (161), antiasthmatic (162), antihypertensive (163), antidiabetic (164), osteoarthritis prevention (165), reduce plasma LDL cholesterol (166), immunomodulatory (167)	Influenza A Virus (168)
Dill or *Anethum graveolens L. (Umbelliferae or Apiaceae)*	antioxidant (169), anti-inflammatory (170), antibacterial (171), anticarcinogen (172), antispasmodic (173), antifungal (174), antihyperlipidemic (175), anti-atherosclerotic (176), antidiabetic (177), mucosal protective (178)	Herpes simplex type-1 (HSV-1) (179), parainfluenza type-3 (PI-3) (179)
Black pepper or *Piper nigrum L. (Piperaceae)*	antioxidant (180-182), anti-inflammatory (183), antibacterial (184), anti-cancer (185, 186), hypolipidemic (187), antifungal (182), anxiolytic (188), antidepressant (188), analgesic (183), hepatoprotective (182), immunomodulatory (186)	Human para influenza virus (189), vesicular stomatitis Indiana virus (189), coxsackie virus type B3 (CVB3) (190)
Acanthopanax henryi (Oliv.) Harms (Araliaceae)	antioxidant (191), anti-inflammatory (192), antibacterial (193), anti-adipogenic (194), neuroprotective (191)	

Boswellia species *(Burseraceae)*	antioxidant (195, 196), anticoagulant (195), anti-inflammatory (196), antibacterial (197), antitumor (198), antifungal (199), anti-adiposity (200), antinociceptive (196), Immunomodulatory (201)	herpes simplex virus type 1 (HSV-1) (202), Influenza virus A (202), Hepatitis C Virus (HCV) (203), vesicular stomatitis virus (VSV) (204), chikungunya virus (CHIKV) (204)
Birch tree *(Betulaceae)*	antioxidant (205), anti-inflammatory (206), antibacterial (207), anti-cancer (208), antinociceptive (206), gastroprotective (209), hepatoprotective (205), Immunomodulatory (210)	herpes simplex virus type 1 (HSV-1) (211, 212), herpes simplex virus type 2 (HSV-2) (212)
Camphor tree or *Cinnamomum camphora* (L.) *(Lauraceae)*	antioxidant (213), anti-inflammatory (214), antibacterial (215), antifungal (216), antigenotoxic (217)	Influenza A virus (218)

Table S6. List of active constituents/phytochemicals from the herbs/plants/trees used in this study.

Herbs/plants/trees (family)	Main or active constituents
Ginger (*Zingiberaceae*)	6-shogaol, 6-gingerol, 8-gingerol, zingiberene (219, 220)
Turmeric or *Curcuma longa L.* *(Zingiberaceae*)	curcumin, demethoxycurcumin, bisdemethoxycurcumin (221)
Elderberry or *Sambucus nigra L.* *(Adoxaceae)*	caffeic acid, 3,4-dihydroxyphenylacetic (57)
Cinnamon *(Lauraceae)*	cinnamaldehyde, cinnamate, cinnamic acid, trans-cinnamaldehyde, eugenol, linalool (222, 223)
Garlic or *Allium sativum L.* *(Liliaceae)*	alliin, allicin (diallyl-dithiosulfinate), diallyl sulfide (DAS), diallyl disulfide (DADS), diallyl trisulfide (DATS), allylMethyl sulfide (AMS), allylmethyl disulfide (AMDS), allylmethyl trisulfide (AMTS) (224-226)
Onion or *Allium cepa L.* *(Liliaceae)*	kaempferol, myricetin, quercetin (227)
Black cumin or *Nigella sativa L.* *(Ranunculaceae)*	thymoquinone, p-cymene, carvacrol, t-anethole, thymol, β-pinene (228)
Peppermint or *Mentha piperita L. (Lamiaceae)*	menthol, menthone (229)
Broccoli or *Brassica oleracea L. var. italica* *(Brassicaceae)*	sulforaphane (SFN) (230)
Dill or *Anethum graveolens L.* *(Umbelliferae or Apiaceae)*	isorhamnetin, kaempferol, quercetin (231)
Black pepper or *Piper nigrum L.* *(Piperaceae)*	piperine (232)
Acanthopanax henryi (Oliv.) Harms (Araliaceae)	savinin (233)
Boswellia species *(Burseraceae)*	boswellic acids (BAs) (234)

Birch tree<br>*(Betulaceae)*	betulinic acid (BA) (235)
Camphor tree or<br>*Cinnamomum camphora* (L.)<br>*(Lauraceae)*	camphor (216)

Table S7. Molecular docking results of herbal compounds with SARS-CoV-2 M^{pro} in the monomeric state, based on affinity of binding. The top-energy pose (according to Total Energy) of each compound is presented (with an RMSD of 0.0). The table also provides 2D chemical structures of the compounds from PubChem. The DockThor molecular docking program was used for analysis of interaction between SARS-CoV-2 M^{pro} monomer and the different ligands, using default settings. Ligplot was used to reveal the residues involved in binding and the mode of interaction with the ligands. The SARS-CoV-2 M^{pro} monomer used in this study was from PDB ID 6yb7.

Herbs/plants/ trees and origin	Residues involved in hydrogen bonding (distance in Å)	Residues involved in hydrophobic interactions	Number of amino acids involved in hydrophobic interactions	DockThor a) Affinity (Kcal/mol) b) Total energy c) Van der Waals Energy d) Electrostatic [illegible]	2D Chemical Structures from pubchem
**Piperine**<br><br>**Pepper**	1. Gly143-N:Ligand-O1 (2.98)	Thr26; Asn142; Met165; Glu166; Gln189	5	a) -8.168<br>b) 9.634<br>c) -21.330<br>d) -3.656	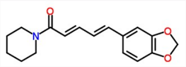
**Isorhamnetin**<br><br>**Dill**	1. Thr25-OG1:Ligand-O6 (2.81)<br>2. Gly143-N:Ligand-O5 (2.86)<br>3. Glu166-O:Ligand-O7 (2.63)	Asn142; Cys145; Met165	3	a) -8.071<br>b) 25.358<br>c) -19.077<br>d) -13.899	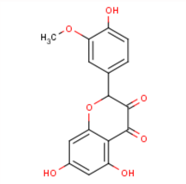
**Frankincense beta-boswellic acid**	-	Phe140; Leu141; Asn142; Cys145; His163; His164; Met165; Glu166	8	a) -7.930<br>b) 1657.809<br>c) -19.811<br>d) -1.447	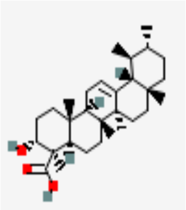
**Myricetin**<br><br>**Onion**	**1.** His41-ND1:Ligand-O6 (2.86)<br>**2.** Thr45-O:Ligand-O8 (2.87)<br>**3.** Gly143-N:Ligand-O4 (2.91)<br>**4.** Ser144-N:Ligand-O4 (3.31)<br>**5.** Ser144-OG:Ligand-O3 (3.32)<br>**6.** Glu166-OE1:Ligand-O5 (2.79)	Thr45; Phe140; Leu141; Asn142; Cys145	5	a) -7.908<br>b) 10.951<br>c) -14.403<br>d) -22.938	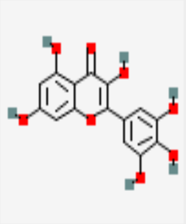

Herbs/plants/ trees and origin	Residues involved in hydrogen bonding (distance in Å)	Residues involved in hydrophobic interactions	Number of amino acids involved in hydrophobic interactions	DockThor a) Affinity (Kcal/mol) b) Total energy c) Van der Waals Energy d) Electrostatic [illegible]	2D Chemical Structures from pubchem
**Eugenol**<br><br>**Cinnamon**	1. Gly143-N:Ligand-O2 (2.99)<br><br>2. Ser144-N:Ligand-O2 (3.34)	Phe140; Leu141; Asn142; Cys145; Met165; Glu166	6	a) -7.654<br>b) 7.922<br>c) -15.290<br>d) -6.352	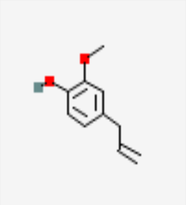
**Desmethoxy-curcumin**<br><br>**Turmeric**	1. Thr24-OG1:Ligand-O1 (2.74)<br>*2.* His163-NE2:Ligand-O4 *(2.29)*	Thr25; Ser46; Met49; Phe140; Leu141; Asn142; Cys145; His164; Met165; Glu166; Gln189	11	a) -7.628<br>b) -4.367<br>c) -0.436<br>d) -28.893	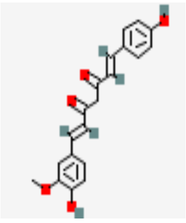
**6-Gingerol**<br><br>**Ginger**	1. Glu166-N:Ligand-O4 (3.14)	Thr25; Thr26; Leu27; His41; Met49; Phe140; Asn142; Gly143; Cys145; His163; His164; Met165	12	a) -7.498<br>b) -3.934<br>c) -19.130<br>d) -9.948	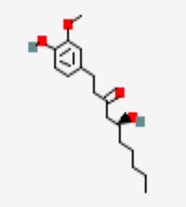
**6-Shogaol**<br><br>**Ginger**	**1.** Gly143-N:Ligand-O1 (2.75)<br>**2.** Glu166-N:Ligand-O3 (2.86)	Asn142; Cys145; Met165; Pro168; Gln189; Thr190	6	a) -7.440<br>b) -8.681<br>c) -13.637<br>d) -14.718	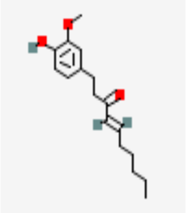
**Menthol**<br><br>**Peppermint**	1. His164-O:Ligand-O (2.85)	His41; Met49; Met165; Glu166; Gln189	5	a) -7.423<br>b) 4.767<br>c) -13.119<br>d) -3.885	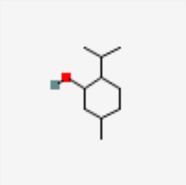
**8-Gingerol**<br><br>**Ginger**	**1.** Gly143-N:Ligand-O3 (2.82)<br>**2.** Cys145-SG:Ligand-O3 (3.22)<br>***3.*** His163-NE2:Ligand-O4 *(2.41)*	His41; Met49; Leu141; Asn142; His164; Met165; Glu166; Pro168; Gln189	9	a) -7.335<br>b) -5.375<br>c) -10.544<br>d) -21.355	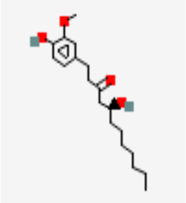
**Bisdementhoxycu rcumin**<br><br>**Turmeric**	1. Thr24-OG1:Ligand-O1 (2.77)<br>*2.* His163-NE2:Ligand-O4 *(2.20)*	Thr25; Ser46; Met49; Phe140; Asn142; Cys145; His164; Met165; Glu166	9	a) -7.311<br>b) -12.114<br>c) 5.102<br>d) -34.564	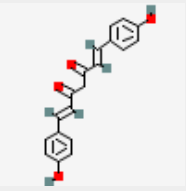

Herbs/plants/ trees and origin	Residues involved in hydrogen bonding (distance in Å)	Residues involved in hydrophobic interactions	Number of amino acids involved in hydrophobic interactions	DockThor<br>a) Affinity (Kcal/mol)<br>b) Total energy<br>c) Van der Waals Energy<br>d) Electrostatic [illegible]	2D Chemical Structures from pubchem
**Diallyl trisulfide**<br><br>**Garlic**	-	Phe140; Leu141; Asn142; His164; Met165; Glu166; Gln189	7	a) -7.277<br>b) -27.535<br>c) -17.267<br>d) -1.561	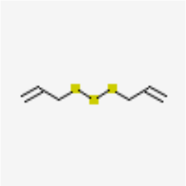
**Kaempferol**<br><br>**Onion/Dill**	**1.** Gly143-N:Ligand-O5 (2.95)<br>***2.*** His163-NE2:Ligand-O6 *(2.25)*	Ser46; Phe140; Leu141; Asn142; Ser144; Cys145; Met165; Glu166; His172	9	a) -7.238<br>b) 99.547<br>c) 2.031<br>d) -29.277	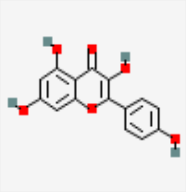
**Menthone**<br><br>**Peppermint**	1. Glu166-N:Ligand-O (2.69)	Met49; Leu141; Asn142; Cys145; Met165	5	a) -7.192<br>b) 6.135<br>c) -10.911<br>d) -5.766	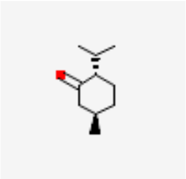
**Zingiberene**<br><br>**Ginger**	-	His41; Asn142; Gly143; Cys145; Met165; Glu166; Pro168; Arg188; Gln189; Thr190	10	a) -7.184<br>b) -6.874<br>c) -15.264<br>d) -3.065	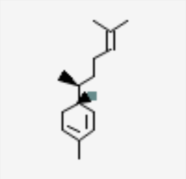
**Allicin**<br><br>**Garlic**	1. Glu166-N:Ligand-O (2.92)	His41; Met49; Leu141; Asn142; Ser144; His164; Met165	7	a) -7.181<br>b) -10.913<br>c) -15.329<br>d) -4.324	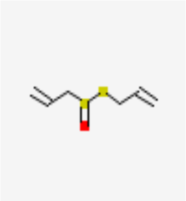
**Quercetin**<br><br>**Onion/Dill**	1. Thr26-N:Ligand-O2 (3.31)<br>**2.** Gly143-N:Ligand-O4 (3.03)<br>3. Glu166-N:Ligand-O5 (2.85)	Thr24; Thr25; Met49; Asn142; Cys145; Met165	6	a) -7.172<br>b) 147.837<br>c) -14.786<br>d) -12.180	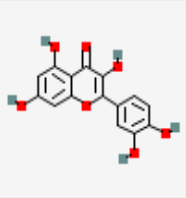
**Allyl methyl trisulfide**<br><br>**Garlic**	-	Met49; Phe140; Asn142; His163; His164; Met165; Glu166	7	a) -7.170<br>b) -29.163<br>c) -15.623<br>d) -0.931	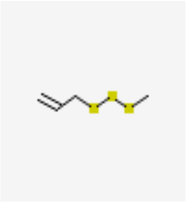

Herbs/plants/ trees and origin	Residues involved in hydrogen bonding (distance in Å)	Residues involved in hydrophobic interactions	Number of amino acids involved in hydrophobic interactions	DockThor a) Affinity (Kcal/mol) b) Total energy c) Van der Waals Energy d) Electrostatic [illegible]	2D Chemical Structures from pubchem
**Camphor**<br><br>**Camphor tree**	1. Glu166-N:Ligand-O (2.68)	Met49; Met165	2	a) -7.158<br>b) 1.170<br>c) -10.461<br>d) -5.902	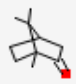
**Diallyl disulfide**<br><br>**Garlic**	-	His41; Phe140; Leu141; Asn142; His164; Met165; Glu166	7	a) -7.120<br>b) -15.258<br>c) -14.897<br>d) -1.979	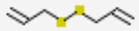
**Cinnamaldehyde**<br><br>**Cinnamon**	1. His163-NE2:Ligand-O (2.67)	His41; Phe140; His164; Met165; Glu166	6	a) -7.001<br>b) -5.883<br>c) -11.242<br>d) -6.090	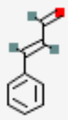
**Allyl methyl disulfide**<br><br>**Garlic**	-	Phe140; Leu141; Asn142; Cys145; His163; Met165; Glu166	7	a) -6.956<br>b) -17.522<br>c) -12.988<br>d) -2.196	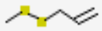
**Diallyl sulfide**<br><br>**Garlic**	-	Phe140; Leu141; Asn142; His164; Met165; Glu166	6	a) -6.915<br>b) -10.556<br>c) -13.443<br>d) -2.096	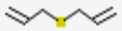
**β-pinene**<br><br>**Black cumin**	-	His41; Met49; His164; Met165; Glu166; Gln189	6	a) -6.865<br>b) 22.587<br>c) -10.185<br>d) -1.398	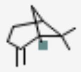
**p-cymene**<br><br>**Black cumin**	-	Phe140; Leu141; Asn142; Gly143; Ser144; Cys145; His163; Glu166	8	a) -6.744<br>b) 5.492<br>c) -10.000<br>d) -4.821	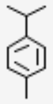

Herbs/plants/ trees and origin	Residues involved in hydrogen bonding (distance in Å)	Residues involved in hydrophobic interactions	Number of amino acids involved in hydrophobic interactions	DockThor a) Affinity (Kcal/mol) b) Total energy c) Van der Waals Energy d) Electrostatic [illegible]	2D Chemical Structures from pubchem
**Sulforaphane**<br><br>**brocolli/ cauliflower/ mustard**	1. His163-NE2:Ligand-O (2.60)	Leu141; Asn142; Cys145; Met165; Glu166; Gln189	6	a) -6.739<br>b) -19.857<br>c) -12.051<br>d) -7.299	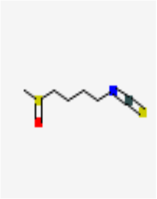
**Thymol**<br><br>**Black cumin**	1. Gly143-N:Ligand-O (2.91)	Phe140; Leu141; Asn142; Ser144; Cys145; His163; Glu166	7	a) -6.655<br>b) 9.230<br>c) -9.220<br>d) -8.068	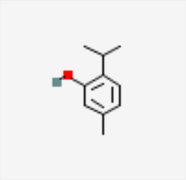
**Allyl methyl sulfide**<br><br>**Garlic**	-	Phe140; Leu141; His163; His164; Met165; Glu166	6	a) -6.642<br>b) -11.017<br>c) -11.018<br>d) -2.885	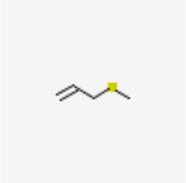
**Cinnamate**<br><br>**Cinnamon**	***1.*** His163-NE2:Ligand-O1 (1.94)	Met49; Phe140; Leu141; Cys145; Met165; His164; Glu166	7	a) -6.557<br>b) -8.101<br>c) 14.246<br>d) -42.097	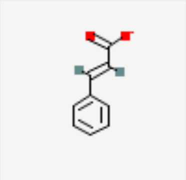
**Thymoquinone**<br><br>**Black cumin**	**1.** Gly143-N:Ligand-O1 (2.74)<br>**2.** Glu166-N:Ligand-O2 (2.81)	His41; Leu141; Asn142; Cys145; His163; Met165	6	a) -6.533<br>b) -7.446<br>c) -9.229<br>d) -10.761	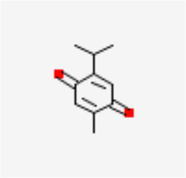
**Cinnamic acid**<br><br>**Cinnamon**	1. His163-NE2:Ligand-O1 (1.95)	Phe140; Leu141; Cys145; Met165; Glu166	6	a) -6.456<br>b) -13.131<br>c) 14.956<br>d) -40.329	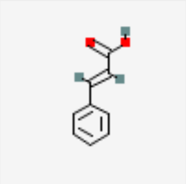
**Carvacrol**<br><br>**Black cumin**	1. Arg188-NH2:Ligand-O (2.91)<br>2. Thr190-OG1:Ligand-O (2.93)	Leu50; Gln189	2	a) -6.453<br>b) 13.043<br>c) -3.811<br>d) -13.245	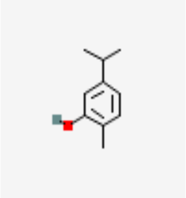

Herbs/plants/ trees and origin	Residues involved in hydrogen bonding (distance in Å)	Residues involved in hydrophobic interactions	Number of amino acids involved in hydrophobic interactions	DockThor a) Affinity (Kcal/mol) b) Total energy c) Van der Waals Energy d) Electrostatic energy	2D Chemical Structures from pubchem
**Caffeic acid**<br><br>**Elderberries**	1. Arg188-NH2:Ligand-O1 (2.78)<br>*2.* Gln189-NE2:Ligand-O3 *(2.46)*	Thr190; Ala191	2	a) -6.374<br>b) 23.321<br>c) 3.261<br>d) -29.924	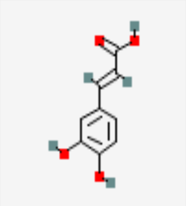
**3,4-dihydroxyphen-ylacetic acid**<br><br>**Elderberries**	1. Arg188-NH2:Ligand-O4 (2.72)<br>2. Gln189-NE2:Ligand-O1 (3.00)<br>*3.* Thr190-OG1:Ligand-O3 *(2.36)*	Leu50; Ala191	2	a) -6.315<br>b) 24.393<br>c) 3.478<br>d) -30.668	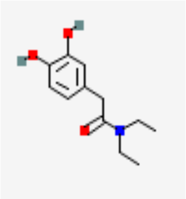
**Alliin**<br><br>**Onion**	1. Ser144-OG:Ligand-O3 (3.20)<br>2. His163-NE2:Ligand-O2 (1.95)	Phe140; Asn142; Cys145; Met165; Glu166	5	a) -6.172<br>b) -22.412<br>c) 16.168<br>d) -50.448	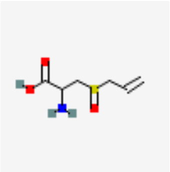

Table S8. Molecular docking results of herbal compounds with SARS-CoV-2 Mpro in the dimeric state, based on affinity of binding. The top-energy pose (according to Total Energy) of each compound is presented (with an RMSD of 0.0). The table also provides 2D chemical structures of the compounds from PubChem. The DockThor molecular docking program was used for analysis of interaction between SARS-CoV-2 Mpro monomer and the different ligands, using default settings. Ligplot was used to reveal the residues involved in binding and the mode of interaction with the ligands. The SARS-CoV-2 Mpro monomer used in this study was from PDB ID 6yb7.

Herbs/plants/ trees and origin	Residues involved in hydrogen bonding (distance in Å)	Residues involved in hydrophobic interactions	Number of amino acids involved in hydrophobic interactions	DockThor a) Affinity (Kcal/mol) b) Total energy c) Van der Waals Energy d) Electrostatic [illegible]	2D Chemical Structures from pubchem
**6-Shogaol**<br><br>**Ginger**	Thr26-O:Ligand-O3 (2.79)	Thr25; His41; Cys44; Ser46: Leu141; Ser144; Cys145	7	a) -8.296<br>b) 24.987<br>c) -23.234<br>d) -3.280	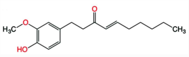
**Piperine**<br><br>**Pepper**	-	Thr25; Thr26; His41; Cys44; Met49; Asn119; Asn142; Tyr118; Gly143	9	a) -8.285<br>b) 11.560<br>c) -23.294<br>d) -0.122	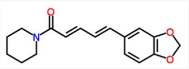
**Frankincense**<br><br>**beta-boswellic acid**	-	Thr24; Thr25; Thr26; His41; Ser46; Met49; Asn119; Asn142; Gly143	9	a) -8.116<br>b) 122.404<br>c) 4.652<br>d) 8.108	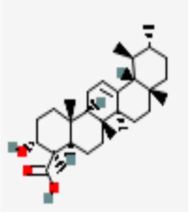
**Desmethoxy-curcumin**<br><br>**Turmeric**	Gly143-N:Ligand-O4 (3.15)<br>Gln189-OE1:Ligand-O5 (2.65)	Thr25; Thr26; His41; Met49; Asn119; Asn142; Cys145	7	a) -8.038<br>b) 26.326<br>c) -21.478<br>d) -9.800	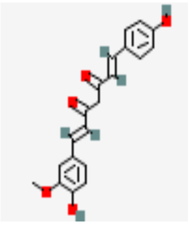
**Zingiberene**<br><br>**Ginger**	-	Thr25; His41; Met49; Leu141; Asn142; Ser144; Cys145; Met165	8	a) -7.995<br>b) 14.834<br>c) -20.337<br>d) -0.307	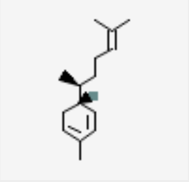

Herbs/plants/ trees and origin	Residues involved in hydrogen bonding (distance in Å)	Residues involved in hydrophobic interactions	Number of amino acids involved in hydrophobic interactions	DockThor a) Affinity (Kcal/mol) b) Total energy c) Van der Waals Energy d) Electrostatic [illegible]	2D Chemical Structures from pubchem
**8-Gingerol**<br><br>**Ginger**	Thr26-N:Ligand-O2 (2.83)<br>Asn142-O:Ligand-O4 (2.81)	Thr25; His41; Met49; Gly143; Tyr118; Asn119; His164	7	a) -7.805<br>b) 19.388<br>c) -22.474<br>d) -5.215	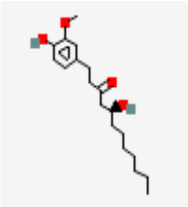
**6-Gingerol**<br><br>**Ginger**	Thr26-O:Ligand-O1 (2.75)<br>His164-O:Ligand-O4 (2.88)	Thr25; His41; Asn119; Asn142; Gly143; Cys145; Met165	7	a) -7.790<br>b) 19.699<br>c) -21.176<br>d) -6.910<br>e) 0.000	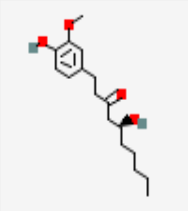
**Isorhamnetin**<br><br>**Dill**	Thr24-O:Ligand-O7 (2.65)<br>Gln189-OE1:Ligand-O6 (2.54)	Thr25; Thr26; His41; Ser46; Asn142; Gly143; Cys145	7	a) -7.758<br>b) 30.511<br>c) -13.577<br>d) -15.337	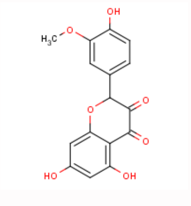
**Bisdementhoxycurcumin**<br><br>**Turmeric**	Asn119-OD1:Ligand-O3 (3.01)<br>Gly143-N:Ligand-O2 (2.93)<br>Gln189-OE1:Ligand-O4 (2.67)	Thr26; Met49; Asn142; Cys145	4	a) -7.742<br>b) 14.417<br>c) -18.119<br>d) -12.027	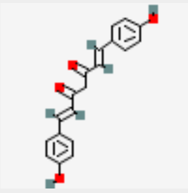
**Kaempferol**<br><br>**Onion/Dill**	Thr24-O:Ligand-O6 (2.80)<br>Gln189-OE1:Ligand-O5 (2.55)	Thr25; Thr26; His41; Ser46; Met49; Asn142; Cys145	7	a) -7.577<br>b) 19.576<br>c) -13.226<br>d) -13.963	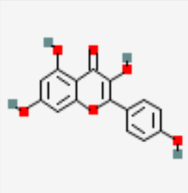
**Quercetin**<br><br>**Onion/Dill**	Thr24-O:Ligand-O7 (2.78)<br>Gln189-OE1:Ligand-O5 (2.54)	Thr25; Thr26; His41; Ser46; Met49; Asn142; Cys145	7	a) -7.576<br>b) 19.628<br>c) -13.192<br>d) -13.919	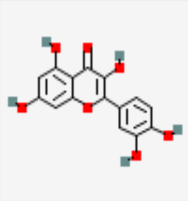

Herbs/plants/ trees and origin	Residues involved in hydrogen bonding (distance in Å)	Residues involved in hydrophobic interactions	Number of amino acids involved in hydrophobic interactions	DockThor a) Affinity (Kcal/mol) b) Total energy c) Van der Waals Energy d) Electrostatic [illegible]	2D Chemical Structures from pubchem
**Myricetin**<br><br>**Onion**	Thr24-O:Ligand-O7 (2.65)<br>His41-ND1:Ligand-O5 (2.73)<br>Asn142-ND2:Ligand-O2 (3.11)	Thr25; Thr26; Met49; Gly143	4	a) -7.546<br>b) 20.464<br>c) -15.001<br>d) -11.606	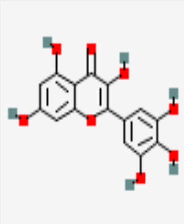
**Eugenol**<br><br>**Cinnamon**	Thr26-O:Ligand-O2 (2.65)<br>Thr26-N:Ligand-O2 (3.25)	Thr25; His41; Cys44; Met49; Asn142; Gly143; Cys145	7	a) -7.488<br>b) 25.677<br>c) -14.819<br>d) -4.712	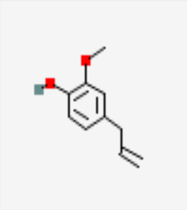
**p-cymene**<br><br>**Black cumin**	-	His41; Met49; Leu141; Asn142; Cys145; His164; Glu166	7	a) -7.467<br>b) 24.993<br>c) -14.967<br>d) 0.080	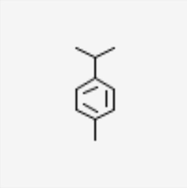
**Carvacrol**<br><br>**Black cumin**	His41-ND1:Ligand-O (2.70)	Thr25; Cys44; Met49; Met165	4	a) -7.399<br>b) 20.109<br>c) -12.159<br>d) -6.382	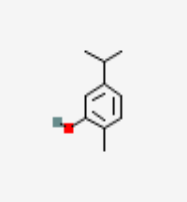
**Diallyl trisulfide**<br><br>**Garlic**	-	Phe140; Leu141; Asn142; Cys145; Met165; Glu166	6	a) -7.391<br>b) -24.637<br>c) -17.025<br>d) -0.358	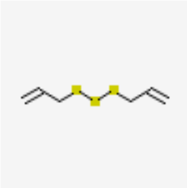
**Thymol**<br><br>**Black cumin**	Asn142-OD1:Ligand-O (2.73)	Thr25; His41; Cys44; Met49	4	a) -7.361<br>b) 20.526<br>c) -13.728<br>d) -4.810	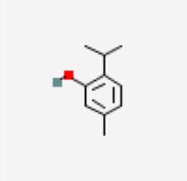
**Alliin**<br><br>**Onion**	Glu166-N:Ligand-O (3.04)	His41; Leu141; His164; Met165	4	a) -7.356<br>b) -6.153<br>c) -15.680<br>d) -2.309	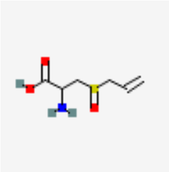

Herbs/plants/ trees and origin	Residues involved in hydrogen bonding (distance in Å)	Residues involved in hydrophobic interactions	Number of amino acids involved in hydrophobic interactions	DockThor a) Affinity (Kcal/mol) b) Total energy c) Van der Waals Energy d) Electrostatic [illegible]	2D Chemical Structures from pubchem
**β-pinene**<br><br>**Black cumin**	-	Thr25; His41; Cys44; Ser46; Met49; Asn142	6	a) -7.353<br>b) 22.322<br>c) -13.400<br>d) -0.138	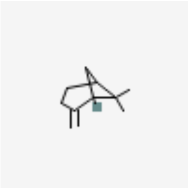
**Menthol**<br><br>**Peppermint**	Asn142-OD1:Ligand-O (2.70)	Thr25; His41; Cys44; Met49	4	a) -7.332<br>b) 9.513<br>c) -12.884<br>d) -4.533	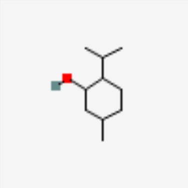
**Menthone**<br><br>**Peppermint**	-	Thr25; Thr26; His41; Ser46; Met49; Asn142	6	a) -7.290<br>b) 8.533<br>c) -13.964<br>d) -0.317	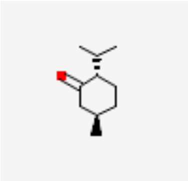
**Sulforaphane**<br><br>**brocolli/ cauliflower/ mustard**	-	His41; Cys44; Met49; His163; His164	6	a) -7.270<br>b) -9.747<br>c) -17.400<br>d) -1.368	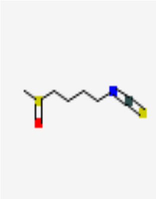
**Thymoquinone**<br><br>**Black cumin**	Gly143-N:Ligand-O2 (2.82)	Thr26; His41; Met49; Asn142; Cys145	5	a) -7.253<br>b) 0.550<br>c) -13.443<br>d) -3.578	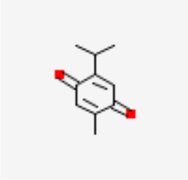
**Allyl methyl trisulfide**<br><br>**Garlic**	-	Met49; Leu141; Ser144; His164; Met165; Glu166	6	a) -7.239<br>b) -26.852<br>c) -15.245<br>d) -0.569	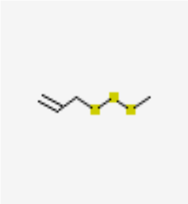
**Camphor**<br><br>**Camphor tree**	-	Thr25; His41; Cys44; Met49; Asn142; Cys145	6	a) -7.221<br>b) 6.695<br>c) -13.363<br>d) -0.135	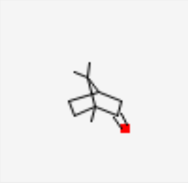

Herbs/plants/ trees and origin	Residues involved in hydrogen bonding (distance in Å)	Residues involved in hydrophobic interactions	Number of amino acids involved in hydrophobic interactions	DockThor a) Affinity (Kcal/mol) b) Total energy c) Van der Waals Energy d) Electrostatic [illegible]	2D Chemical Structures from pubchem
**Diallyl disulfide**<br><br>**Garlic**	-	His41; Cys44; Met49; Leu141; His164	5	a) -7.219<br>b) -9.888<br>c) -14.744<br>d) -0.427	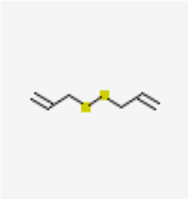
**Allicin**<br><br>**Garlic**	Glu166-N:Ligand-O (3.01)	His41; Leu141; Asn142; His164; Met165	5	a) -7.197<br>b) -6.159<br>c) -15.326<br>d) -2.526	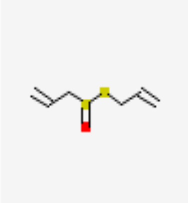
**Cinnamaldehyde**<br><br>**Cinnamon**	His163-NE2:Ligand-O (2.61)	His41; Phe140; Asn142; Cys145; His164; Glu166; His172	7	a) -7.122<br>b) 2.256<br>c) -13.246<br>d) -4.873	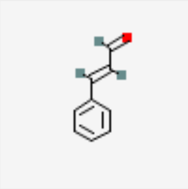
**Allyl methyl disulfide**<br><br>**Garlic**	-	Phe140; Leu141; His164; Met165; Glu166	5	a) -7.058<br>b) -14.558<br>c) -13.433<br>d) -0.282	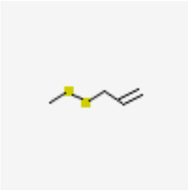
**Diallyl sulfide**<br><br>**Garlic**	-	Phe140; Leu141; Ser144; His163	4	a) -6.994<br>b) -5.316<br>c) -13.551<br>d) -0.388	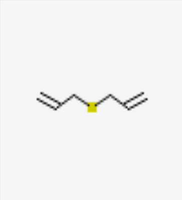
**Caffeic acid**<br><br>**Elderberries**	His163-NE2:Ligand-O3 (2.48)<br>Gln189-OE1:Ligand-O1 (3.00)<br>Gln189-OE1:Ligand-O2 (2.82)	Met49; Leu141; Asn142; Cys145; Met165; Glu166	6	a) -6.758<br>b) -6.677<br>c) -10.157<br>d) -16.387	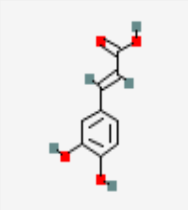
**Allyl methyl sulfide**<br><br>**Garlic**	-	Phe140; Leu141; His163; His164; Met165; Glu166	6	a) -6.748<br>b) -7.333<br>c) -10.626<br>d) -1.492	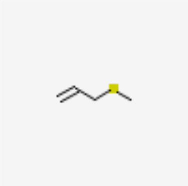

Herbs/plants/ trees and origin	Residues involved in hydrogen bonding (distance in Å)	Residues involved in hydrophobic interactions	Number of amino acids involved in hydrophobic interactions	DockThor a) Affinity (Kcal/mol) b) Total energy c) Van der Waals Energy d) Electrostatic [illegible]	2D Chemical Structures from pubchem
**3,4-dihydroxyphen-ylacetic acid**<br><br>**Elderberries**	Asn142-OD1:Ligand-O1 (2.65)<br>Ser144-OG:Ligand-O4 (2.83)<br>Cys145-SG:Ligand-O4 (3.31)<br>His163-NE2:Ligand-O3 (2.21)	His41; Phe140; Leu141; His164	4	a) -6.691<br>b) 9.694<br>c) -3.507<br>d) -23.581	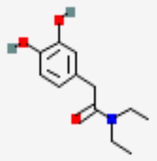
**Cinnamic acid**<br><br>**Cinnamon**	His163-NE2:Ligand-O1 (1.98)	Phe140; Leu141; Cys145; His164; Met165; Glu166	6	a) -6.668<br>b) -1.514<br>c) 1.285<br>d) -23.305	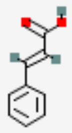
**Cinnamate**<br><br>**Cinnamon**	His163-NE2:Ligand-O1 (2.12)	Phe140; Leu141; Cys145; His164; Met165; Glu166	6	a) -6.629<br>b) -2.256<br>c) -4.028<br>d) -17.470	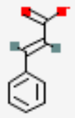

Table S9. List of specific therapeutic properties and antiviral effects of savinin, betulinic acid and curcumin.

Active constituent/ Phytochemical	Therapeutics Properties	Antiviral Effects
Savinin	Antioxidant (236), anti-inflammatory (237), anti-cancer (238), antiestrogenic (239), neuroprotective (240)	SARS-CoV (241)
Betulinic acid (BA)	Antioxidant (242), antiplatelet (243), anti-inflammatory (244), antibacterial (245), anti-cancer (246), antinociceptive (247), antimalarial (248), antifungal (249), anti-obesity (250), hepatoprotective (251), antihypertensive (252), antidiabetic (253), antidepressant (254), immunomodulatory (255)	SARS-CoV (241), human immunodeficiency virus (HIV) (256, 257), herpes simplex virus type 1 (HSV-1) (211, 258, 259), ECHO 6 virus (259), Influenza A/PR/8 Virus (260), Hepatitis B virus (HBV) (261), Hepatitis C virus (HCV) (262)
Curcumin	Antioxidant (37, 263), anticoagulant (264), anti-inflammatory (265), antibacterial (266), anti-cancer (267), antiparasitic (268), antifungal (269), Antifibrotic (270), anti-endometriotic (271), antidiabetic (272), anti-obesity (273), antidepressant (274), anti-arthritic (275), radioprotective (276), neuroprotective (277), immunomodulatory (278), cholesterol-lowering (279), wound healing (280), treat Alzheimer's disease (281)	SARS-CoV (241), Transmissible gastroenteritis virus (TGEV) (282), Respiratory syncytial virus (RSV) (283), Porcine reproductive and respiratory syndrome virus (PRRSV) (284), herpes simplex virus type 1 (HSV-1) (285), Herpes simplex virus type 2 (HSV-2) (286), Kaposi's sarcoma-associated herpesvirus (KSHV) (287), Epstein-Barr virus (EBV) (288), Human cytomegalovirus (HCMV) (289), human immunodeficiency virus-1 (HIV-1) (286, 290-294), vesicular stomatitis virus (VSV) (204), chikungunya virus (CHIKV) (204, 295), Zika virus (ZIKV) (295), Coxsackievirus B3 (CVB3) (296), Enterovirus 71 (EV71) (297), Hepatitis B virus (HBV) (298, 299), Hepatitis C virus (HCV) (300, 301), Influenza A virus (IAV) (302, 303), Human norovirus (HuNoV) (304), Japanese encephalitis virus (JEV) (305), dengue virus type 2 (306), Rift Valley fever virus (RVFV) (307), Ebola virus (308), Viral hemorrhagic septicemia virus (VHSV) (309)

Table S10. Description of experimentally derived binding interactions of a non-covalent inhibitor and the only approved covalent inhibitor of SARS-CoV-2 M^{pro}. The PDB IDs included 7l0d and 7te0, respectively. Ligplot was used to reveal the residues involved in binding interactions as well as the mode of interactions. The 2D chemical structures of the inhibitors were obtained from the deposited structure files.

Ligand/ inhibitor	PDB ID	Residues involved in binding			Structure	Ref
		Hydrophobic bonds	Hydrogen bonds (distance in Å)	Covalent bonds		
**ML188**<br><br>**(A non-covalent inhibitor)**	7l0d	Thr25; Thr26; Leu27; His41; Met49; Leu141; Phe140; Ser144; Cys145; His164; Glu166; Asp187; Arg188; Gln189	Asn142-ND2:Ligand-O1 (2.96) Gly143-N:Ligand-O1 (3.09) Gly143-N:Ligand-O3 (3.20) His163-NE2:Ligand-N3 (2.90)		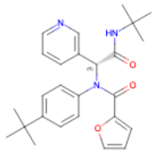	(310)
**Nirmatrelvir**<br><br>**(An approved inhibitor)**	7te0	His41; Met49; Phe140; Asn142; Gly143; Met165; His172; Gln189	Cys145-SG:Ligand-N1 (2.85) His163-NE2:Ligand-O1 (2.57) His164-O:Ligand-N1 (2.88) Glu166-OE2:Ligand-N2 (3.23) Glu166-N:Ligand-O3 (2.81) Glu166-O:Ligand-N4 (2.78) Gln192-NE2:Ligand-F1 (3.11)	Cys145-SG:Ligand-C3	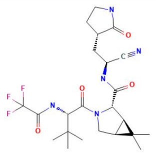	(311)

Table S11. Molecular docking results showing the binding interactions of a non-covalent inhibitor and the only approved covalent inhibitor of SARS-CoV-2 M^{pro}**.** The PDB IDs included 7l0d and 7te0, respectively. DockThor was used for molecular docking using the monomeric state of SARS-CoV-2 M^{pro}, PDB ID 6yb7 for the monomeric states and PDB ID 7ali for the dimeric state. LigPlot was used to reveal the residues involved in binding interactions as well as the mode of interactions.

Ligand/Inhibitor	Residues involved in hydrogen bonding (distance in Å)	Residues involved in hydrophobic interactions	Number of amino acids involved in hydrophobic interactions	Dockthor<br>a) Affinity (Kcal/mol)<br>b) Total energy<br>c) Van der Waals Energy<br>d) Electrostatic energy
**ML188**<br><br>**(A non-covalent inhibitor)**	1. His163-NE2:Ligand-N3 (2.89)<br><br>2. Glu166-N:Ligand-O1 (2.86)	His41; Ser46; Phe140; Asn142; Gly143; Cys145; His164; Met165; Gln189	9	a) -8.882<br>b) 72.557<br>c) -24.657<br>d) -8.171
**Nirmatrelvir**<br><br>**(An approved inhibitor)**<br><br>**Using monomeric state of M**^{pro}	1. Gly143-N:Ligand-O4 (2.79)	Ser46; Phe140; Asn142; Cys145; His163; Glu166; Leu167; Pro168; Gln189	9	a) -7.793<br>b) 39.877<br>c) -19.275<br>d) -11.458
**Nirmatrelvir**<br><br>**(An approved inhibitor)**<br><br>**Using dimeric state of M**^{pro}	1. Glu166-N:Ligand-F1 (3.30)	Thr25; Thr26; Leu27; His41; Ser46; Met49; Asn119; Asn142; Gly143; Cys145; Gln189	11	a) -8.404<br>b) 78.669<br>c) -4.267<br>d) 2.282

Table S12: ADMET analysis of savinin, betulinic acid, curcumin and Nirmatrelvir. LogS: Log of the aqueous solubility. Optimal -4to0.5 log mol/L. **LogP:** Log of the octanol/water partition coefficient. Optimal 0to3. **LogD:** LogP at physiological pH 7.4. Optimal: 1to3. **Caco-2 permeability:** Optimal: higher than -5.15 Log unit. **MDCK permeability:** low: <$2x10^{-6}$ cm/s; medium: 2-20x 10^{-6} cm/s; high passive permeability: >$20x10^{-6}$ cm/s. **PPB:** Plasma protein binding; optimal: <90%. **VD:** Volume distribution; optimal: 0.04-20L/kg. **Fu:** The fraction unbound in plasma; Low: <5%; Middle: 5-20%; High: >20%. **CYP1A2 inhibitor:** The output value is the probability of being inhibitor. **CL:** Clearance; High: >15 mL/min/kg; moderate: 5-15 mL/min/kg; low: <5 mL/min/kg. **Carcinogenicity:** The output value is the probability of being toxic. Green shows favoured values while red presents unfavoured values.

Compound	Mol. Weight g/mol	Physicochemical property			Absorption (Permeability)		Distribution			Metabolism	Excretion	Toxicity
		logS	LogD pH7.4	LogP	Caco Log unit	MDCK cm/s	PPB %	VD L/kg	Fu %	CYP1A2 inhibitor	CL mL/min/	Carcinogencity
**Savinin**	352.090	-5.474	3.809	4.186	-5.028	2.9e-05 High	99.89	0.765	1.468 Low	0.988	16.907 High	0.914
**Betulinic Acid**	456.360	-4.434	6.005	5.459	-5.248	1.4e-05 High	96.71	0.859	2.919 Low	0.01	3.87 Low	0.015
**Curcumin**	368.130	-3.921	2.742	2.82	-4.834	1.6e-05 High	99.79	0.369	1.049 Low	0.593	13.839 High	0.706
**Nirmatrelvir**	499.240	-3.789	2.013	2.033	-5.346	3.5e-05 High	51.77	0.669	51.9 High	0.003	3.416 Low	0.119

References

1. Jin Z, Du X, Xu Y, Deng Y, Liu M, Zhao Y, et al. Structure of Mpro from SARS-CoV-2 and discovery of its inhibitors. Nature. 2020;582(7811):289-93.
2. Yang H, Xie W, Xue X, Yang K, Ma J, Liang W, et al. Design of wide-spectrum inhibitors targeting coronavirus main proteases. PLoS biology. 2005;3(10):e324.
3. Xue X, Yu H, Yang H, Xue F, Wu Z, Shen W, et al. Structures of two coronavirus main proteases: implications for substrate binding and antiviral drug design. Journal of virology. 2008;82(5):2515-27.
4. Ren Z, Yan L, Zhang N, Guo Y, Yang C, Lou Z, Rao Z. The newly emerged SARS-like coronavirus HCoV-EMC also has an" Achilles' heel": current effective inhibitor targeting a 3C-like protease. Protein & cell. 2013;4(4):248.
5. Wang F, Chen C, Tan W, Yang K, Yang H. Structure of main protease from human coronavirus NL63: insights for wide spectrum anti-coronavirus drug design. Scientific reports. 2016;6(1):22677.
6. Zhang L, Lin D, Sun X, Curth U, Drosten C, Sauerhering L, et al. Crystal structure of SARS-CoV-2 main protease provides a basis for design of improved α-ketoamide inhibitors. Science. 2020;368(6489):409-12.
7. Ma C, Sacco MD, Hurst B, Townsend JA, Hu Y, Szeto T, et al. Boceprevir, GC-376, and calpain inhibitors II, XII inhibit SARS-CoV-2 viral replication by targeting the viral main protease. Cell research. 2020;30(8):678-92.
8. Dai W, Zhang B, Jiang X-M, Su H, Li J, Zhao Y, et al. Structure-based design of antiviral drug candidates targeting the SARS-CoV-2 main protease. Science. 2020;368(6497):1331-5.
9. Ballester P, Cerdá B, Arcusa R, García-Muñoz AM, Marhuenda J, Zafrilla P. Antioxidant Activity in Extracts from Zingiberaceae Family: Cardamom, Turmeric, and Ginger. Molecules. 2023;28(10):4024.
10. Kikuzaki H, Nakatani N. Antioxidant effects of some ginger constituents. Journal of food science. 1993;58(6):1407-10.
11. Nile SH, Park SW. Chromatographic analysis, antioxidant, anti-inflammatory, and xanthine oxidase inhibitory activities of ginger extracts and its reference compounds. Industrial Crops and Products. 2015;70:238-44.
12. Srivastava K. Effects of aqueous extracts of onion, garlic and ginger on platelet aggregation and metabolism of arachidonic acid in the blood vascular system: in vitro study. Prostaglandins, Leukotrienes and Medicine. 1984;13(2):227-35.
13. Grzanna R, Lindmark L, Frondoza CG. Ginger—an herbal medicinal product with broad anti-inflammatory actions. Journal of medicinal food. 2005;8(2):125-32.
14. Park M, Bae J, Lee DS. Antibacterial activity of [10]-gingerol and [12]-gingerol isolated from ginger rhizome against periodontal bacteria. Phytotherapy Research: An International Journal Devoted to Pharmacological and Toxicological Evaluation of Natural Product Derivatives. 2008;22(11):1446-9.
15. Katiyar SK, Agarwal R, Mukhtar H. Inhibition of tumor promotion in SENCAR mouse skin by ethanol extract of Zingiber officinale rhizome. Cancer research. 1996;56(5):1023-30.

16. Yamamoto-Ribeiro MMG, Grespan R, Kohiyama CY, Ferreira FD, Mossini SAG, Silva EL, et al. Effect of Zingiber officinale essential oil on Fusarium verticillioides and fumonisin production. Food chemistry. 2013;141(3):3147-52.
17. Bhandari U, Sharma J, Zafar R. The protective action of ethanolic ginger (Zingiber officinale) extract in cholesterol fed rabbits. Journal of Ethnopharmacology. 1998;61(2):167-71.
18. Ghayur MN, Gilani AH. Species differences in the prokinetic effects of ginger. International journal of food sciences and nutrition. 2006;57(1-2):65-73.
19. Ghayur MN, Gilani AH. Ginger lowers blood pressure through blockade of voltage-dependent calcium channels. Journal of cardiovascular pharmacology. 2005;45(1):74-80.
20. Chen B-H, Wu P-Y, Chen K-M, Fu T-F, Wang H-M, Chen C-Y. Antiallergic potential on RBL-2H3 cells of some phenolic constituents of Zingiber officinale (ginger). Journal of natural products. 2009;72(5):950-3.
21. Al-Amin ZM, Thomson M, Al-Qattan KK, Peltonen-Shalaby R, Ali M. Anti-diabetic and hypolipidaemic properties of ginger (Zingiber officinale) in streptozotocin-induced diabetic rats. British journal of nutrition. 2006;96(4):660-6.
22. Okamoto M, Irii H, Tahara Y, Ishii H, Hirao A, Udagawa H, et al. Synthesis of a new [6]-gingerol analogue and its protective effect with respect to the development of metabolic syndrome in mice fed a high-fat diet. Journal of Medicinal Chemistry. 2011;54(18):6295-304.
23. Chen J-C, Huang L-J, Wu S-L, Kuo S-C, Ho T-Y, Hsiang C-Y. Ginger and its bioactive component inhibit enterotoxigenic Escherichia coli heat-labile enterotoxin-induced diarrhea in mice. Journal of agricultural and food chemistry. 2007;55(21):8390-7.
24. Vishwakarma S, Pal S, Kasture VS, Kasture S. Anxiolytic and antiemetic activity of Zingiber officinale. Phytotherapy Research: An International Journal Devoted to Pharmacological and Toxicological Evaluation of Natural Product Derivatives. 2002;16(7):621-6.
25. Wilasrusmee C, Kittur S, Siddiqui J, Bruch D, Wilasrusmee S, Kittur DS. In vitro immunomodulatory effects of ten commonly used herbs on murine lymphocytes. The Journal of Alternative & Complementary Medicine. 2002;8(4):467-75.
26. Amran AZ, Jantan I, Dianita R, Buang F. Protective effects of the standardized extract of Zingiber officinale on myocardium against isoproterenol-induced biochemical and histopathological alterations in rats. Pharmaceutical biology. 2015;53(12):1795-802.
27. Jagetia GC, Baliga MS, Venkatesh P, Ulloor JN. Influence of ginger rhizome (Zingiber officinale Rosc) on survival, glutathione and lipid peroxidation in mice after whole-body exposure to gamma radiation. Radiation research. 2003;160(5):584-92.
28. Schnitzler P, Koch C, Reichling Jr. Susceptibility of drug-resistant clinical herpes simplex virus type 1 strains to essential oils of ginger, thyme, hyssop, and sandalwood. Antimicrobial agents and chemotherapy. 2007;51(5):1859-62.
29. Koch C, Reichling J, Schneele J, Schnitzler P. Inhibitory effect of essential oils against herpes simplex virus type 2. Phytomedicine. 2008;15(1-2):71-8.
30. San Chang J, Wang KC, Yeh CF, Shieh DE, Chiang LC. Fresh ginger (Zingiber officinale) has anti-viral activity against human respiratory syncytial virus in human respiratory tract cell lines. Journal of ethnopharmacology. 2013;145(1):146-51.
31. Ahmed I, Aslam A, Mustafa G, Masood S, Ali MA, Nawaz M. Anti-avian influenza virus H9N2 activity of aqueous extracts of Zingiber officinalis (Ginger) and Allium sativum (Garlic) in chick embryos. Pak J Pharm Sci. 2017;30(4):1341-4.
32. Sookkongwaree K, Geitmann M, Roengsumran S, Petsom A, Danielson UH. Inhibition of viral proteases by Zingiberaceae extracts and flavones isolated from Kaempferia parviflora. Die Pharmazie-An International Journal of Pharmaceutical Sciences. 2006;61(8):717-21.

33. Denyer CV, Jackson P, Loakes DM, Ellis MR, Young DA. Isolation of antirhinoviral sesquiterpenes from ginger (Zingiber officinale). Journal of natural products. 1994;57(5):658-62.
34. Kaushik S, Jangra G, Kundu V, Yadav JP, Kaushik S. Anti-viral activity of Zingiber officinale (Ginger) ingredients against the Chikungunya virus. Virusdisease. 2020;31:270-6.
35. Aboubakr HA, Nauertz A, Luong NT, Agrawal S, El-Sohaimy SA, Youssef MM, Goyal SM. In vitro antiviral activity of clove and ginger aqueous extracts against feline calicivirus, a surrogate for human norovirus. Journal of food protection. 2016;79(6):1001-12.
36. Camero M, Lanave G, Catella C, Capozza P, Gentile A, Fracchiolla G, et al. Virucidal activity of ginger essential oil against caprine alphaherpesvirus-1. Veterinary microbiology. 2019;230:150-5.
37. Jayaprakasha GK, Rao LJ, Sakariah KK. Antioxidant activities of curcumin, demethoxycurcumin and bisdemethoxycurcumin. Food chemistry. 2006;98(4):720-4.
38. Li C, Miao X, Li F, Adhikari BK, Liu Y, Sun J, et al. Curcuminoids: Implication for inflammation and oxidative stress in cardiovascular diseases. Phytotherapy Research. 2019;33(5):1302-17.
39. Kim KJ, Yu HH, Cha JD, Seo SJ, Choi NY, You YO. Antibacterial activity of Curcuma longa L. against methicillin-resistant Staphylococcus aureus. Phytotherapy Research: An International Journal Devoted to Pharmacological and Toxicological Evaluation of Natural Product Derivatives. 2005;19(7):599-604.
40. Yue GG, Chan BC, Hon P-M, Lee MY, Fung K-P, Leung P-C, Lau CB. Evaluation of in vitro anti-proliferative and immunomodulatory activities of compounds isolated from Curcuma longa. Food and chemical toxicology. 2010;48(8-9):2011-20.
41. Rasmussen HB, Christensen SB, Kvist LP, Karazmi A. A simple and efficient separation of the curcumins, the antiprotozoal constituents of Curcuma longa. Planta medica. 2000;66(04):396-8.
42. Lee S-H, Chang K-S, Su M-S, Huang Y-S, Jang H-D. Effects of some Chinese medicinal plant extracts on five different fungi. Food control. 2007;18(12):1547-54.
43. Nishiyama T, Mae T, Kishida H, Tsukagawa M, Mimaki Y, Kuroda M, et al. Curcuminoids and sesquiterpenoids in turmeric (Curcuma longa L.) suppress an increase in blood glucose level in type 2 diabetic KK-Ay mice. Journal of Agricultural and food Chemistry. 2005;53(4):959-63.
44. Ho JN, Jang JY, Yoon HG, Kim Y, Kim S, Jun W, Lee J. Anti-obesity effect of a standardised ethanol extract from Curcuma longa L. fermented with Aspergillus oryzae in ob/ob mice and primary mouse adipocytes. Journal of the Science of Food and Agriculture. 2012;92(9):1833-40.
45. Yu Z, Kong L, Chen Y. Antidepressant activity of aqueous extracts of Curcuma longa in mice. Journal of Ethnopharmacology. 2002;83(1-2):161-5.
46. Orellana-Paucar AM, Serruys A-SK, Afrikanova T, Maes J, De Borggraeve W, Alen J, et al. Anticonvulsant activity of bisabolene sesquiterpenoids of Curcuma longa in zebrafish and mouse seizure models. Epilepsy & behavior. 2012;24(1):14-22.
47. Song E-K, Cho H, Kim J-S, Kim N-Y, An N-H, Kim J-A, et al. Diarylheptanoids with free radical scavenging and hepatoprotective activity in vitro from Curcuma longa. Planta medica. 2001;67(09):876-7.
48. Kutluay SB, Doroghazi J, Roemer ME, Triezenberg SJ. Curcumin inhibits herpes simplex virus immediate-early gene expression by a mechanism independent of p300/CBP histone acetyltransferase activity. Virology. 2008;373(2):239-47.
49. Balasubramanyam K, Varier RA, Altaf M, Swaminathan V, Siddappa NB, Ranga U, Kundu TK. Curcumin, a novel p300/CREB-binding protein-specific inhibitor of acetyltransferase, represses the acetylation of histone/nonhistone proteins and histone acetyltransferase-dependent chromatin transcription. Journal of Biological Chemistry. 2004;279(49):51163-71.

50. Han S, Xu J, Guo X, Huang M. Curcumin ameliorates severe influenza pneumonia via attenuating lung injury and regulating macrophage cytokines production. Clinical and Experimental Pharmacology and Physiology. 2018;45(1):84-93.
51. Kim HJ, Yoo HS, Kim JC, Park CS, Choi MS, Kim M, et al. Antiviral effect of Curcuma longa Linn extract against hepatitis B virus replication. Journal of ethnopharmacology. 2009;124(2):189-96.
52. Colpitts CC, Schang LM, Rachmawati H, Frentzen A, Pfaender S, Behrendt P, et al. Turmeric curcumin inhibits entry of all hepatitis C virus genotypes into human liver cells. Gut. 2014;63(7):1137-49.
53. Ichsyani M, Ridhanya A, Risanti M, Desti H, Ceria R, Putri D, et al., editors. Antiviral effects of Curcuma longa L. against dengue virus in vitro and in vivo. IOP Conference Series: Earth and Environmental Science; 2017: IOP Publishing.
54. Haş IM, Teleky B-E, Szabo K, Simon E, Ranga F, Diaconeasa ZM, et al. Bioactive Potential of Elderberry (Sambucus nigra L.): Antioxidant, Antimicrobial Activity, Bioaccessibility and Prebiotic Potential. Molecules. 2023;28(7):3099.
55. Wu X, Gu L, Prior RL, McKay S. Characterization of anthocyanins and proanthocyanidins in some cultivars of Ribes, Aronia, and Sambucus and their antioxidant capacity. Journal of agricultural and food chemistry. 2004;52(26):7846-56.
56. Dawidowicz AL, Wianowska D, Baraniak B. The antioxidant properties of alcoholic extracts from Sambucus nigra L.(antioxidant properties of extracts). LWT-Food Science and Technology. 2006;39(3):308-15.
57. Ho GTT, Wangensteen H, Barsett H. Elderberry and elderflower extracts, phenolic compounds, and metabolites and their effect on complement, RAW 264.7 macrophages and dendritic cells. International journal of molecular sciences. 2017;18(3):584.
58. Krawitz C, Mraheil MA, Stein M, Imirzalioglu C, Domann E, Pleschka S, Hain T. Inhibitory activity of a standardized elderberry liquid extract against clinically-relevant human respiratory bacterial pathogens and influenza A and B viruses. BMC complementary and alternative medicine. 2011;11:1-6.
59. Goun EA, Petrichenko V, Solodnikov S, Suhinina T, Kline MA, Cunningham G, et al. Anticancer and antithrombin activity of Russian plants. Journal of ethnopharmacology. 2002;81(3):337-42.
60. Gray AM, Abdel-Wahab YH, Flatt PR. The traditional plant treatment, Sambucus nigra (elder), exhibits insulin-like and insulin-releasing actions in vitro. The Journal of nutrition. 2000;130(1):15-20.
61. Mahmoudi M, Ebrahimzadeh M, Dooshan A, Arimi A, Ghasemi N, Fathiazad F. Antidepressant activities of Sambucus ebulus and Sambucus nigra. Eur Rev Med Pharmacol Sci. 2014;18(22):3350-3.
62. Ataee R, Falahati A, Ebrahimzadeh M, Shokrzadeh M. Anticonvulsant activities of Sambucus nigra. Eur Rev Med Pharmacol Sci. 2016;20(14):3123-6.
63. Frøkiær H, Henningsen L, Metzdorff SB, Weiss G, Roller M, Flanagan J, et al. Astragalus root and elderberry fruit extracts enhance the IFN-β stimulatory effects of Lactobacillus acidophilus in murine-derived dendritic cells. PLoS One. 2012;7(10):e47878.
64. Chen C, Zuckerman DM, Brantley S, Sharpe M, Childress K, Hoiczyk E, Pendleton AR. Sambucus nigra extracts inhibit infectious bronchitis virus at an early point during replication. BMC veterinary research. 2014;10(1):1-12.
65. Serkedjieva J, Manolova N, Zgórniak-Nowosielska I, Zawilińska B, Grzybek J. Antiviral activity of the infusion (SHS-174) from flowers of Sambucus nigra L., aerial parts of Hypericum

perforatum L., and roots of Saponaria officinalis L. against influenza and herpes simplex viruses. Phytotherapy Research. 1990;4(3):97-100.
66. Kinoshita E, Hayashi K, Katayama H, Hayashi T, Obata A. Anti-influenza virus effects of elderberry juice and its fractions. Bioscience, biotechnology, and biochemistry. 2012;76(9):1633-8.
67. Zakay-Rones Z, Thom E, Wollan T, Wadstein J. Randomized study of the efficacy and safety of oral elderberry extract in the treatment of influenza A and B virus infections. Journal of International Medical Research. 2004;32(2):132-40.
68. Zakay-Rones Z, Varsano N, Zlotnik M, Manor O, Regev L, Schlesinger M, Mumcuoglu M. Inhibition of several strains of influenza virus in vitro and reduction of symptoms by an elderberry extract (Sambucus nigra L.) during an outbreak of influenza B Panama. The Journal of Alternative and Complementary Medicine. 1995;1(4):361-9.
69. Fink RC, Roschek Jr B, Alberte RS. HIV type-1 entry inhibitors with a new mode of action. Antiviral Chemistry and Chemotherapy. 2009;19(6):243-55.
70. Manganelli RU, Zaccaro L, Tomei P. Antiviral activity in vitro of Urtica dioica L., Parietaria diffusa M. et K. and Sambucus nigra L. Journal of ethnopharmacology. 2005;98(3):323-7.
71. Liu S, Zhao C, Cao Y, Li Y, Zhang Z, Nie D, et al. Comparison of Chemical Compositions and Antioxidant Activity of Essential Oils from Litsea Cubeba, Cinnamon, Anise, and Eucalyptus. Molecules. 2023;28(13):5051.
72. Shan B, Cai YZ, Sun M, Corke H. Antioxidant capacity of 26 spice extracts and characterization of their phenolic constituents. Journal of agricultural and food chemistry. 2005;53(20):7749-59.
73. Roussel A-M, Hininger I, Benaraba R, Ziegenfuss TN, Anderson RA. Antioxidant effects of a cinnamon extract in people with impaired fasting glucose that are overweight or obese. Journal of the American College of Nutrition. 2009;28(1):16-21.
74. Tung Y-T, Chua M-T, Wang S-Y, Chang S-T. Anti-inflammation activities of essential oil and its constituents from indigenous cinnamon (Cinnamomum osmophloeum) twigs. Bioresource technology. 2008;99(9):3908-13.
75. Zhang Y, Liu X, Wang Y, Jiang P, Quek S. Antibacterial activity and mechanism of cinnamon essential oil against Escherichia coli and Staphylococcus aureus. Food Control. 2016;59:282-9.
76. Ka H, Park H-J, Jung H-J, Choi J-W, Cho K-S, Ha J, Lee K-T. Cinnamaldehyde induces apoptosis by ROS-mediated mitochondrial permeability transition in human promyelocytic leukemia HL-60 cells. Cancer letters. 2003;196(2):143-52.
77. Ayatollahi Mousavi SA, Kazemi A. In vitro and in vivo antidermatophytic activities of some Iranian medicinal plants. Medical mycology. 2015;53(8):852-9.
78. Preuss HG, Echard B, Polansky MM, Anderson R. Whole cinnamon and aqueous extracts ameliorate sucrose-induced blood pressure elevations in spontaneously hypertensive rats. Journal of the American College of Nutrition. 2006;25(2):144-50.
79. Nagai H, SHIMAZAWA T, MATSUURA N, KODA A. Immunopharmacological studies of the aqueous extract of Cinnamomum cassia (CCAq) I. Anti-allergic Action. The Japanese Journal of Pharmacology. 1982;32(5):813-22.
80. Kim SH, Hyun SH, Choung SY. Anti-diabetic effect of cinnamon extract on blood glucose in db/db mice. Journal of ethnopharmacology. 2006;104(1-2):119-23.
81. Tanaka S, Yoon YH, Fukui H, Tabata M, Akira T, Okano K, et al. Antiulcerogenic compounds isolated from Chinese cinnamon. Planta medica. 1989;55(03):245-8.
82. Kurokawa M, Kumeda CA, Yamamura J-i, Kamiyama T, Shiraki K. Antipyretic activity of cinnamyl derivatives and related compounds in influenza virus-infected mice. European journal of pharmacology. 1998;348(1):45-51.

83. Premanathan M, Rajendran S, Ramanathan T, Kathiresan K. A survey of some Indian medicinal plants for anti-human immunodeficiency virus (HIV) activity. Indian Journal of medical research. 2000;112:73.
84. Hayashi K, Imanishi N, Kashiwayama Y, Kawano A, Terasawa K, Shimada Y, Ochiai H. Inhibitory effect of cinnamaldehyde, derived from Cinnamomi cortex, on the growth of influenza A/PR/8 virus in vitro and in vivo. Antiviral Research. 2007;74(1):1-8.
85. Lu J, Li N, Li S, Liu W, Li M, Zhang M, Chen H. Biochemical composition, antioxidant activity and antiproliferative effects of different processed garlic products. Molecules. 2023;28(2):804.
86. Imai J, Ide N, Nagae S, Moriguchi T, Matsuura H, Itakura Y. Antioxidant and radical scavenging effects of aged garlic extract and its constituents. Planta medica. 1994;60(05):417-20.
87. Ryu K, Ide N, Matsuura H, Itakura Y. N α-(1-deoxy-d-fructos-1-yl)-l-arginine, an antioxidant compound identified in aged garlic extract. The Journal of nutrition. 2001;131(3):972S-6S.
88. Park SY, Seetharaman R, Ko MJ, Kim TH, Yoon MK, Kwak JH, et al. Ethyl linoleate from garlic attenuates lipopolysaccharide-induced pro-inflammatory cytokine production by inducing heme oxygenase-1 in RAW264. 7 cells. International Immunopharmacology. 2014;19(2):253-61.
89. O'Gara EA, Hill DJ, Maslin DJ. Activities of garlic oil, garlic powder, and their diallyl constituents against Helicobacter pylori. Applied and environmental microbiology. 2000;66(5):2269-73.
90. Myneni AA, Chang S-C, Niu R, Liu L, Swanson MK, Li J, et al. Raw garlic consumption and lung cancer in a Chinese population. Cancer Epidemiology, Biomarkers & Prevention. 2016;25(4):624-33.
91. Dkhil M, Abdel-Baki A, Wunderlich F, Sies H, Al-Quraishy S. Anticoccidial and antiinflammatory activity of garlic in murine Eimeria papillata infections. Veterinary Parasitology. 2011;175(1-2):66-72.
92. Yousuf S, Ahmad A, Khan A, Manzoor N, Khan LA. Effect of garlic-derived allyl sulphides on morphogenesis and hydrolytic enzyme secretion in Candida albicans. Medical mycology. 2011;49(4):444-8.
93. Asdaq S, Inamdar M. Potential of garlic and its active constituent, S-allyl cysteine, as antihypertensive and cardioprotective in presence of captopril. Phytomedicine. 2010;17(13):1016-26.
94. Sobenin IA, Andrianova IV, Lakunin KY, Karagodin VP, Bobryshev YV, Orekhov AN. Anti-atherosclerotic effects of garlic preparation in freeze injury model of atherosclerosis in cholesterol-fed rabbits. Phytomedicine. 2016;23(11):1235-9.
95. Zhai B, Zhang C, Sheng Y, Zhao C, He X, Xu W, et al. Hypoglycemic and hypolipidemic effect of S-allyl-cysteine sulfoxide (alliin) in DIO mice. Scientific reports. 2018;8(1):3527.
96. Yang C, Li L, Yang L, Lǚ H, Wang S, Sun G. Anti-obesity and Hypolipidemic effects of garlic oil and onion oil in rats fed a high-fat diet. Nutrition & metabolism. 2018;15(1):1-8.
97. Li M, Yan YX, Yu QT, Deng Y, Wu DT, Wang Y, et al. Comparison of immunomodulatory effects of fresh garlic and black garlic polysaccharides on RAW 264.7 macrophages. Journal of food science. 2017;82(3):765-71.
98. Tsai Y, Cole LL, Davis LE, Lockwood SJ, Simmons V, Wild GC. Antiviral properties of garlic: in vitro effects on influenza B, herpes simplex and coxsackie viruses. Planta medica. 1985;51(05):460-1.
99. Weber ND, Andersen DO, North JA, Murray BK, Lawson LD, Hughes BG. In vitro virucidal effects of Allium sativum (garlic) extract and compounds. Planta medica. 1992;58(05):417-23.

100. Guo N-l, Lu D-p, Gail LW, Elizabeth R, Zhou G-z, Zhang L-b, Robert HW. Demonstration of the anti-viral activity of garlic extract against human cytomegalovirus in vitro. Chinese medical journal. 1993;106(02):93-6.
101. Tatarintsev A, Vrzhets P, Ershov D, Shchegolev A, Turgiev A, Karamov E, et al. The ajoene blockade of integrin-dependent processes in an HIV-infected cell system. Vestnik Rossiiskoi akademii meditsinskikh nauk. 1992(11-12):6-10.
102. Gouda M, Nassarawa SS, Gupta SD, Sanusi NIi, Nasiru MM. Evaluation of carbon dioxide elevation on phenolic compounds and antioxidant activity of red onion (Allium cepa L.) during postharvest storage. Plant Physiology and Biochemistry. 2023;200:107752.
103. Ramos FA, Takaishi Y, Shirotori M, Kawaguchi Y, Tsuchiya K, Shibata H, et al. Antibacterial and antioxidant activities of quercetin oxidation products from yellow onion (Allium cepa) skin. Journal of agricultural and food chemistry. 2006;54(10):3551-7.
104. Ye C-L, Dai D-H, Hu W-L. Antimicrobial and antioxidant activities of the essential oil from onion (Allium cepa L.). Food control. 2013;30(1):48-53.
105. Debaene J, Goldman I, Yandell B. Postharvest flux and genotype× environment effects for onion-induced antiplatelet activity, pungency, and soluble solids in long-day onion during postharvest cold storage. Journal of the American Society for Horticultural Science. 1999;124(4):366-72.
106. Wilson EA, Demmig-Adams B. Antioxidant, anti-inflammatory, and antimicrobial properties of garlic and onions. Nutrition & food science. 2007;37(3):178-83.
107. Galeone C, Pelucchi C, Levi F, Negri E, Franceschi S, Talamini R, et al. Onion and garlic use and human cancer. The American journal of clinical nutrition. 2006;84(5):1027-32.
108. Saleheen D, Ali SA, Yasinzai MM. Antileishmanial activity of aqueous onion extract in vitro. Fitoterapia. 2004;75(1):9-13.
109. Škerget M, Majhenič L, Bezjak M, Knez Ž. Antioxidant, radical scavenging and antimicrobial activities of red onion (Allium cepa L) skin and edible part extracts. Chemical and Biochemical Engineering Quarterly. 2009;23(4):435-44.
110. Srinivasan K. Plant foods in the management of diabetes mellitus: spices as beneficial antidiabetic food adjuncts. International journal of food sciences and nutrition. 2005;56(6):399-414.
111. Romeilah RM, Fayed SA, Mahmoud GI. Chemical compositions, antiviral and antioxidant activities of seven essential oils. Journal of Applied Sciences Research. 2010;6(1):50-62.
112. Van Damme EJ, Smeets K, Engelborghs I, Aelbers H, Balzarini J, Pusztai A, et al. Cloning and characterization of the lectin cDNA clones from onion, shallot and leek. Plant molecular biology. 1993;23:365-76.
113. Chen C-H, Chou T-W, Cheng L-H, Ho C-W. In vitro anti-adenoviral activity of five Allium plants. Journal of the Taiwan Institute of Chemical Engineers. 2011;42(2):228-32.
114. Harazem R, El Rahman SA, El-Kenawy A. Evaluation of Antiviral Activity of Allium Cepa and Allium Sativum Extracts Against Newcastle Disease Virus. Alexandria Journal for Veterinary Sciences. 2019;61(1).
115. Ismail N, Abd Ghafar SA, Bakar MZA. Antioxidant activity and phenolic content of black cumin seeds. Biochemistry, Nutrition, and Therapeutics of Black Cumin Seed: Elsevier; 2023. p. 169-88.
116. Hosseinzadeh H, Taiari S, Nassiri-Asl M. Effect of thymoquinone, a constituent of Nigella sativa L., on ischemia–reperfusion in rat skeletal muscle. Naunyn-Schmiedeberg's archives of pharmacology. 2012;385:503-8.
117. El Gazzar M, El Mezayen R, Marecki JC, Nicolls MR, Canastar A, Dreskin SC. Anti-inflammatory effect of thymoquinone in a mouse model of allergic lung inflammation. International immunopharmacology. 2006;6(7):1135-42.

118. Kumar TS, Negi P, Sankar KU. Antibacterial Activity of" Nigella sativa L." Seed Extracts. British journal of pharmacology and toxicology. 2010;1(2):96-100.
119. Schneider-Stock R, Fakhoury IH, Zaki AM, El-Baba CO, Gali-Muhtasib HU. Thymoquinone: fifty years of success in the battle against cancer models. Drug discovery today. 2014;19(1):18-30.
120. Okeola VO, Adaramoye OA, Nneji CM, Falade CO, Farombi EO, Ademowo OG. Antimalarial and antioxidant activities of methanolic extract of Nigella sativa seeds (black cumin) in mice infected with Plasmodium yoelli nigeriensis. Parasitology research. 2011;108:1507-12.
121. Aljabre SHM, Randhawa MA, Akhtar N, Alakloby OM, Alqurashi AM, Aldossary A. Antidermatophyte activity of ether extract of Nigella sativa and its active principle, thymoquinone. Journal of Ethnopharmacology. 2005;101(1-3):116-9.
122. Boskabady M, Mohsenpoor N, Takaloo L. Antiasthmatic effect of Nigella sativa in airways of asthmatic patients. Phytomedicine. 2010;17(10):707-13.
123. Kaatabi H, Bamosa AO, Badar A, Al-Elq A, Abou-Hozaifa B, Lebda F, et al. Nigella sativa improves glycemic control and ameliorates oxidative stress in patients with type 2 diabetes mellitus: placebo controlled participant blinded clinical trial. PloS one. 2015;10(2):e0113486.
124. Namazi N, Larijani B, Ayati MH, Abdollahi M. The effects of Nigella sativa L. on obesity: A systematic review and meta-analysis. Journal of ethnopharmacology. 2018;219:173-81.
125. Sayeed MSB, Shams T, Hossain SF, Rahman MR, Mostofa A, Kadir MF, et al. Nigella sativa L. seeds modulate mood, anxiety and cognition in healthy adolescent males. Journal of Ethnopharmacology. 2014;152(1):156-62.
126. Umar S, Zargan J, Umar K, Ahmad S, Katiyar CK, Khan HA. Modulation of the oxidative stress and inflammatory cytokine response by thymoquinone in the collagen induced arthritis in Wistar rats. Chemico-biological interactions. 2012;197(1):40-6.
127. Hosseinzadeh H, Parvardeh S. Anticonvulsant effects of thymoquinone, the major constituent of Nigella sativa seeds, in mice. Phytomedicine. 2004;11(1):56-64.
128. Al-Naggar T, Gomez-Serranillos M, Carretero M, Villar A. Neuropharmacological activity of Nigella sativa L. extracts. Journal of ethnopharmacology. 2003;88(1):63-8.
129. Ebru U, Burak U, Yusuf S, Reyhan B, Arif K, Faruk TH, et al. Cardioprotective effects of Nigella sativa oil on cyclosporine A-induced cardiotoxicity in rats. Basic & clinical pharmacology & toxicology. 2008;103(6):574-80.
130. El-Abhar H, Abdallah D, Saleh S. Gastroprotective activity of Nigella sativa oil and its constituent, thymoquinone, against gastric mucosal injury induced by ischaemia/reperfusion in rats. Journal of ethnopharmacology. 2003;84(2-3):251-8.
131. Yildiz F, Coban S, Terzi A, Ates M, Aksoy N, Cakir H, et al. Nigella sativa relieves the deleterious effects of ischemia reperfusion injury on liver. World journal of gastroenterology: WJG. 2008;14(33):5204.
132. Fararh K, Atoji Y, Shimizu Y, Shiina T, Nikami H, Takewaki T. Mechanisms of the hypoglycaemic and immunopotentiating effects of Nigella sativa L. oil in streptozotocin-induced diabetic hamsters. Research in veterinary science. 2004;77(2):123-9.
133. Salem ML, Hossain MS. Protective effect of black seed oil from Nigella sativa against murine cytomegalovirus infection. International journal of immunopharmacology. 2000;22(9):729-40.
134. Zihlif MA, Mahmoud IS, Ghanim MT, Zreikat MS, Alrabadi N, Imraish A, et al. Thymoquinone efficiently inhibits the survival of EBV-infected B cells and alters EBV gene expression. Integrative cancer therapies. 2013;12(3):257-63.

135. Onifade AA, Jewell AP, Adedeji WA. Nigella sativa concoction induced sustained seroreversion in HIV patient. African Journal of Traditional, Complementary and Alternative Medicines. 2013;10(5):332-5.
136. Umar S, Munir MT, Subhan S, Azam T, Nisa Q, Khan MI, et al. Protective and antiviral activities of Nigella sativa against avian influenza (H9N2) in turkeys. J Saudi Soc Agric Sci. 2016;10.
137. Dorra N, El-Berrawy M, Sallam S, Mahmoud R. Evaluation of antiviral and antioxidant activity of selected herbal extracts. Journal of High Institute of Public Health. 2019;49(1):36-40.
138. Barakat EMF, El Wakeel LM, Hagag RS. Effects of Nigella sativa on outcome of hepatitis C in Egypt. World journal of gastroenterology: WJG. 2013;19(16):2529.
139. Abdel-Moneim A, Morsy BM, Mahmoud AM, Abo-Seif MA, Zanaty MI. Beneficial therapeutic effects of Nigella sativa and/or Zingiber officinale in HCV patients in Egypt. EXCLI journal. 2013;12:943.
140. El-Shafi S. Preliminary studies on antibacterial and antiviral activities of five medicinal plants. J Plant Pathol Microb. 2013;4:190.
141. Wang B, Yuan X, Zhu Y, Wang Y, Li D, He J, Xiao Y. Low-level cadmium exposure induced hormesis in peppermint young plant by constantly activating antioxidant activity based on physiological and transcriptomic analyses. Frontiers in Plant Science. 2023;14:1088285.
142. Dragland S, Senoo H, Wake K, Holte K, Blomhoff R. Several culinary and medicinal herbs are important sources of dietary antioxidants. The Journal of nutrition. 2003;133(5):1286-90.
143. Singh R, Shushni MA, Belkheir A. Antibacterial and antioxidant activities of Mentha piperita L. Arabian Journal of Chemistry. 2015;8(3):322-8.
144. Sun Z, Wang H, Wang J, Zhou L, Yang P. Chemical composition and anti-inflammatory, cytotoxic and antioxidant activities of essential oil from leaves of Mentha piperita grown in China. PloS one. 2014;9(12):e114767.
145. Kumar A, Samarth R, Yasmeen S, Sharma A, Sugahara T, Terado T, Kimura H. Anticancer and radioprotective potentials of Mentha piperita. Biofactors. 2004;22(1-4):87-92.
146. Nikesh M, Binitha G, Rekha S, Ravindra N, Anto Shering M. Comparative in vitro anthelmintic activity of chloroform and acetone extracts of Mentha piperita. Drugs. 2010;19:20.
147. Tampieri MP, Galuppi R, Macchioni F, Carelle MS, Falcioni L, Cioni PL, Morelli I. The inhibition of Candida albicans by selected essential oils and their major components. Mycopathologia. 2005;159:339-45.
148. Asao T, Mochiki E, Suzuki H, Nakamura J-i, Hirayama I, Morinaga N, et al. An easy method for the intraluminal administration of peppermint oil before colonoscopy and its effectiveness in reducing colonic spasm. Gastrointestinal endoscopy. 2001;53(2):172-7.
149. Inoue T, Sugimoto Y, Masuda H, Kamei C. Antiallergic effect of flavonoid glycosides obtained from Mentha piperita L. Biological and Pharmaceutical Bulletin. 2002;25(2):256-9.
150. Barbalho SM, Damasceno DC, Spada APM, Silva VSd, Martuchi KA, Oshiiwa M, et al. Metabolic profile of offspring from diabetic Wistar rats treated with Mentha piperita (peppermint). Evidence-Based Complementary and Alternative Medicine. 2011;2011.
151. Atta A, Alkofahi A. Anti-nociceptive and anti-inflammatory effects of some Jordanian medicinal plant extracts. Journal of ethnopharmacology. 1998;60(2):117-24.
152. Morice A, Marshall A, Higgins K, Grattan T. Effect of inhaled menthol on citric acid induced cough in normal subjects. Thorax. 1994;49(10):1024-6.
153. Li Y, Liu Y, Ma A, Bao Y, Wang M, Sun Z. In vitro antiviral, anti-inflammatory, and antioxidant activities of the ethanol extract of Mentha piperita L. Food science and biotechnology. 2017;26:1675-83.

154. Schuhmacher A, Reichling J, Schnitzler P. Virucidal effect of peppermint oil on the enveloped viruses herpes simplex virus type 1 and type 2 in vitro. Phytomedicine. 2003;10(6-7):504-10.
155. Yamasaki K, Nakano M, Kawahata T, MORI H, OTAKE T, UEDA N, et al. Anti-HIV-1 activity of herbs in Labiatae. Biological and Pharmaceutical Bulletin. 1998;21(8):829-33.
156. HAMEED A, FATIMA N, IFTIKHAR H, MEHMOOD A, TARIQ MR, ALI SW, et al. Effect of different drying and cooking treatments on phytochemicals and antioxidant activity in broccoli: an experimental in vitro study. Food Science and Technology. 2023;43:e101622.
157. Sun T, Powers JR, Tang J. Evaluation of the antioxidant activity of asparagus, broccoli and their juices. Food chemistry. 2007;105(1):101-6.
158. Jang HW, Moon J-K, Shibamoto T. Analysis and antioxidant activity of extracts from broccoli (Brassica oleracea L.) sprouts. Journal of agricultural and food chemistry. 2015;63(4):1169-74.
159. López-Chillón MT, Carazo-Díaz C, Prieto-Merino D, Zafrilla P, Moreno DA, Villaño D. Effects of long-term consumption of broccoli sprouts on inflammatory markers in overweight subjects. Clinical Nutrition. 2019;38(2):745-52.
160. Moon J-K, Kim J-R, Ahn Y-J, Shibamoto T. Analysis and anti-Helicobacter activity of sulforaphane and related compounds present in broccoli (Brassica oleracea L.) sprouts. Journal of Agricultural and Food Chemistry. 2010;58(11):6672-7.
161. Li Y, Zhang T, Korkaya H, Liu S, Lee H-F, Newman B, et al. Sulforaphane, a dietary component of broccoli/broccoli sprouts, inhibits breast cancer stem cells. Clinical Cancer Research. 2010;16(9):2580-90.
162. Brown RH, Reynolds C, Brooker A, Talalay P, Fahey JW. Sulforaphane improves the bronchoprotective response in asthmatics through Nrf2-mediated gene pathways. Respiratory research. 2015;16:1-12.
163. Wu L, Ashraf MHN, Facci M, Wang R, Paterson PG, Ferrie A, Juurlink BH. Dietary approach to attenuate oxidative stress, hypertension, and inflammation in the cardiovascular system. Proceedings of the National Academy of Sciences. 2004;101(18):7094-9.
164. Axelsson AS, Tubbs E, Mecham B, Chacko S, Nenonen HA, Tang Y, et al. Sulforaphane reduces hepatic glucose production and improves glucose control in patients with type 2 diabetes. Science translational medicine. 2017;9(394):eaah4477.
165. Davidson R, Gardner S, Jupp O, Bullough A, Butters S, Watts L, et al. Isothiocyanates are detected in human synovial fluid following broccoli consumption and can affect the tissues of the knee joint. Scientific reports. 2017;7(1):3398.
166. Armah CN, Derdemezis C, Traka MH, Dainty JR, Doleman JF, Saha S, et al. Diet rich in high glucoraphanin broccoli reduces plasma LDL cholesterol: Evidence from randomised controlled trials. Molecular nutrition & food research. 2015;59(5):918-26.
167. Busato B, de Almeida Abreu EC, de Oliveira Petkowicz CL, Martinez GR, Noleto GR. Pectin from Brassica oleracea var. italica triggers immunomodulating effects in vivo. International Journal of Biological Macromolecules. 2020;161:431-40.
168. Rashan L, Hakkim FL, Idrees M, Essa MM, Velusamy T, Al-Baloshi M, et al. Boswellia gum resin and essential oils: Potential health benefits− An evidence based review. International Journal of Nutrition, Pharmacology, Neurological Diseases. 2019;9(2):53-71.
169. Mohammad Al-Ismail, K. and T. Aburjai, *Antioxidant activity of water and alcohol extracts of chamomile flowers, anise seeds and dill seeds.* Journal of the Science of Food and Agriculture, 2004. **84**(2): p. 173-178
170. Naseri M, Mojab F, Khodadoost M, Kamalinejad M, Davati A, Choopani R, et al. The study of anti-inflammatory activity of oil-based dill (Anethum graveolens L.) extract used topically in

formalin-induced inflammation male rat paw. Iranian journal of pharmaceutical research: IJPR. 2012;11(4):1169.
171. Singh G, Kapoor I, Pandey S, Singh U, Singh R. Studies on essential oils: part 10; antibacterial activity of volatile oils of some spices. Phytotherapy Research: An International Journal Devoted to Pharmacological and Toxicological Evaluation of Natural Product Derivatives. 2002;16(7):680-2.
172. Zheng G-q, Kenney PM, Lam LK. Anethofuran, carvone, and limonene: potential cancer chemoprotective agents from dill weed oil and caraway oil. Planta medica. 1992;58(04):338-41.
173. Naseri MG, Heidari A. Antispasmodic effect of Anethum graveolens fruit extract on rat ileum. Int J Pharmacol. 2007;3(3):260-4.
174. Tian J, Ban X, Zeng H, Huang B, He J, Wang Y. In vitro and in vivo activity of essential oil from dill (Anethum graveolens L.) against fungal spoilage of cherry tomatoes. Food Control. 2011;22(12):1992-9.
175. Hajhashemi V, Abbasi N. Hypolipidemic activity of Anethum graveolens in rats. Phytotherapy Research: An International Journal Devoted to Pharmacological and Toxicological Evaluation of Natural Product Derivatives. 2008;22(3):372-5.
176. Setorki M, Rafieian-Kopaei M, Merikhi A, Heidarian E, Shahinfard N, Ansari R, et al. Suppressive impact of anethum graveolens consumption on biochemical risk factors of atherosclerosis in hypercholesterolemic rabbits. International Journal of Preventive Medicine. 2013;4(8):889.
177. Panda S. The effect of Anethum graveolens L.(dill) on corticosteroid induced diabetes mellitus: involvement of thyroid hormones. Phytotherapy Research: An International Journal Devoted to Pharmacological and Toxicological Evaluation of Natural Product Derivatives. 2008;22(12):1695-7.
178. Hosseinzadeh H, Karimi GR, Ameri M. Effects of Anethum graveolens L. seed extracts on experimental gastric irritation models in mice. BMC pharmacology. 2002;2(1):1-5.
179. Orhan İE, ÖZÇELİK B, Kartal M, Kan Y. Antimicrobial and antiviral effects of essential oils from selected Umbelliferae and Labiatae plants and individual essential oil components. Turkish Journal of Biology. 2012;36(3):239-46.
180. Mollik M, Rahman MH, Al-Shaeri M, Ashraf GM, Alexiou A, Gafur MA. Isolation, characterization and in vitro antioxidant activity screening of pure compound from black pepper (Piper nigrum). Environmental Science and Pollution Research. 2022;29(34):52220-32.
181. Kapoor I, Singh B, Singh G, De Heluani CS, De Lampasona M, Catalan CA. Chemistry and in vitro antioxidant activity of volatile oil and oleoresins of black pepper (Piper nigrum). Journal of agricultural and food chemistry. 2009;57(12):5358-64.
182. Zhang C, Zhao J, Famous E, Pan S, Peng X, Tian J. Antioxidant, hepatoprotective and antifungal activities of black pepper (Piper nigrum L.) essential oil. Food Chemistry. 2021;346:128845.
183. Tasleem F, Azhar I, Ali SN, Perveen S, Mahmood ZA. Analgesic and anti-inflammatory activities of Piper nigrum L. Asian Pacific journal of tropical medicine. 2014;7:S461-S8.
184. Zhang J, Ye K-P, Zhang X, Pan D-D, Sun Y-Y, Cao J-X. Antibacterial activity and mechanism of action of black pepper essential oil on meat-borne Escherichia coli. Frontiers in microbiology. 2017;7:2094.
185. Sriwiriyajan S, Sukpondma Y, Srisawat T, Madla S, Graidist P. (−)-Kusunokinin and piperloguminine from Piper nigrum: An alternative option to treat breast cancer. Biomedicine & Pharmacotherapy. 2017;92:732-43.

186. Majdalawieh AF, Carr RI. In vitro investigation of the potential immunomodulatory and anti-cancer activities of black pepper (Piper nigrum) and cardamom (Elettaria cardamomum). Journal of Medicinal Food. 2010;13(2):371-81.
187. VIJAYAKUMAR RS, SURYA D, SENTHILKUMAR R, NALINI N. Hypolipidemic effect of black pepper (Piper nigrum Linn.) in rats fed high fat diet. Journal of clinical biochemistry and nutrition. 2002;32:31-42.
188. Hritcu L, Noumedem JA, Cioanca O, Hancianu M, Postu P, Mihasan M. Anxiolytic and antidepressant profile of the methanolic extract of Piper nigrum fruits in beta-amyloid (1–42) rat model of Alzheimer's disease. Behavioral and Brain Functions. 2015;11(1):1-13.
189. Priya N, Kumari PS. Antiviral activities and cytotoxicity assay of seed extracts of Piper longum and Piper nigrum on human cell lines. International Journal of Pharmaceutical Sciences Review and Research. 2017;44(1):197-202.
190. Mair C, Liu R, Atanasov A, Schmidtke M, Dirsch V, Rollinger J. Antiviral and anti-proliferative in vitro activities of piperamides from black pepper. Planta Medica. 2016;82(S 01):P807.
191. Zhang XD, Liu XQ, Kim YH, Whang WK. Chemical constituents and their acetyl cholinesterase inhibitory and antioxidant activities from leaves of Acanthopanax henryi: potential complementary source against Alzheimer's disease. Archives of pharmacal research. 2014;37:606-16.
192. Jong-Hwan K, Xiang-Qian L, Ling D, Chang-Soo Y, Kyung-Tae L. Cytotoxicity and anti-inflammatory effects of root bark extracts of Acanthopanax henryi. Chinese journal of natural medicines. 2014;12(2):121-5.
193. Li QQ, Luo J, Liu XQ, Kwon DY, Kang OH. Eleutheroside K isolated from Acanthopanax henryi (Oliv.) Harms suppresses methicillin resistance of Staphylococcus aureus. Letters in Applied Microbiology. 2021;72(6):669-76.
194. Han Y-H, Li Z, Um J-Y, Liu XQ, Hong S-H. Anti-adipogenic effect of Glycoside St-E2 and Glycoside St-C1 isolated from the leaves of Acanthopanax henryi (Oliv.) Harms in 3T3-L1 cells. Bioscience, Biotechnology, and Biochemistry. 2016;80(12):2391-400.
195. Kokkiripati PK, Bhakshu LM, Marri S, Padmasree K, Row AT, Raghavendra AS, Tetali SD. Gum resin of Boswellia serrata inhibited human monocytic (THP-1) cell activation and platelet aggregation. Journal of ethnopharmacology. 2011;137(1):893-901.
196. Mothana RA. Anti-inflammatory, antinociceptive and antioxidant activities of the endemic Soqotraen Boswellia elongata Balf. f. and Jatropha unicostata Balf. f. in different experimental models. Food and Chemical Toxicology. 2011;49(10):2594-9.
197. Mothana RA, Lindequist U. Antimicrobial activity of some medicinal plants of the island Soqotra. Journal of ethnopharmacology. 2005;96(1-2):177-81.
198. Khan MA, Ali R, Parveen R, Najmi AK, Ahmad S. Pharmacological evidences for cytotoxic and antitumor properties of Boswellic acids from Boswellia serrata. Journal of ethnopharmacology. 2016;191:315-23.
199. El-Nagerabi SA, Elshafie AE, AlKhanjari SS, Al-Bahry SN, Elamin MR. Biological activities of Boswellia sacra extracts on the growth and aflatoxins secretion of two aflatoxigenic species of Aspergillus species. Food control. 2013;34(2):763-9.
200. Gomaa AA, Farghaly HS, El-Sers DA, Farrag MM, Al-Zokeim NI. Inhibition of adiposity and related metabolic disturbances by polyphenol-rich extract of Boswellia serrata gum through alteration of adipo/cytokine profiles. Inflammopharmacology. 2019;27:549-59.
201. Ammon H. Modulation of the immune system by Boswellia serrata extracts and boswellic acids. Phytomedicine. 2010;17(11):862-7.

202. Mothana RA, Mentel R, Reiss C, Lindequist U. Phytochemical screening and antiviral activity of some medicinal plants from the island Soqotra. Phytotherapy Research: An International Journal Devoted to Pharmacological and Toxicological Evaluation of Natural Product Derivatives. 2006;20(4):298-302.
203. Hussein G, Miyashiro H, Nakamura N, Hattori M, Kakiuchi N, Shimotohno K. Inhibitory effects of Sudanese medicinal plant extracts on hepatitis C virus (HCV) protease. Phytotherapy Research: An International Journal Devoted to Pharmacological and Toxicological Evaluation of Natural Product Derivatives. 2000;14(7):510-6.
204. von Rhein C, Weidner T, Henß L, Martin J, Weber C, Sliva K, Schnierle BS. Curcumin and Boswellia serrata gum resin extract inhibit chikungunya and vesicular stomatitis virus infections in vitro. Antiviral Research. 2016;125:51-7.
205. Matsuda H, Ishikado A, Nishida N, Ninomiya K, Fujiwara H, Kobayashi Y, Yoshikawa M. Hepatoprotective, superoxide scavenging, and antioxidative activities of aromatic constituents from the bark of Betula platyphylla var. japonica. Bioorganic & medicinal chemistry letters. 1998;8(21):2939-44.
206. Huh J-E, Hong J-M, Baek Y-H, Lee J-D, Choi D-Y, Park D-S. Anti-inflammatory and anti-nociceptive effect of Betula platyphylla var. japonica in human interleukin-1β-stimulated fibroblast-like synoviocytes and in experimental animal models. Journal of Ethnopharmacology. 2011;135(1):126-34.
207. Duric K, Kovac-Besovic E, Niksic H, Sofic E. Antibacterial activity of methanolic extracts, decoction and isolated triterpene products from different parts of birch, Betula pendula, Roth. Journal of Plant Studies. 2013;2(2):61.
208. So HM, Eom HJ, Lee D, Kim S, Kang KS, Lee IK, et al. Bioactivity evaluations of betulin identified from the bark of Betula platyphylla var. japonica for cancer therapy. Archives of pharmacal research. 2018;41:815-22.
209. Germanò MP, Cacciola F, Donato P, Dugo P, Certo G, D'Angelo V, et al. Betula pendula Roth leaves: gastroprotective effects of an HPLC-fingerprinted methanolic extract. Natural product research. 2013;27(17):1569-75.
210. Gutermuth J, Bewersdorff M, Traidl-Hoffmann C, Ring J, Mueller MJ, Behrendt H, Jakob T. Immunomodulatory effects of aqueous birch pollen extracts and phytoprostanes on primary immune responses in vivo. Journal of allergy and clinical immunology. 2007;120(2):293-9.
211. Navid MH, Laszczyk-Lauer M, Reichling J, Schnitzler P. Pentacyclic triterpenes in birch bark extract inhibit early step of herpes simplex virus type 1 replication. Phytomedicine. 2014;21(11):1273-80.
212. Gong Y, Raj KM, Luscombe CA, Gadawski I, Tam T, Chu J, et al. The synergistic effects of betulin with acyclovir against herpes simplex viruses. Antiviral Research. 2004;64(2):127-30.
213. Hu J-N, Zhang B, Zhu X-M, Li J, Fan Y-W, Liu R, et al. Characterization of medium-chain triacylglycerol (MCT)-enriched seed oil from Cinnamomum camphora (Lauraceae) and its oxidative stability. Journal of agricultural and food chemistry. 2011;59(9):4771-8.
214. Lee HJ, Hyun E-A, Yoon WJ, Kim BH, Rhee MH, Kang HK, et al. In vitro anti-inflammatory and anti-oxidative effects of Cinnamomum camphora extracts. Journal of ethnopharmacology. 2006;103(2):208-16.
215. Marasini BP, Baral P, Aryal P, Ghimire KR, Neupane S, Dahal N, et al. Evaluation of antibacterial activity of some traditionally used medicinal plants against human pathogenic bacteria. BioMed research international. 2015;2015.
216. Pragadheesh V, Saroj A, Yadav A, Chanotiya C, Alam M, Samad A. Chemical characterization and antifungal activity of Cinnamomum camphora essential oil. Industrial crops and products. 2013;49:628-33.

217. Bakkali F, Averbeck S, Averbeck D, Zhiri A, Baudoux D, Idaomar M. Antigenotoxic effects of three essential oils in diploid yeast (Saccharomyces cerevisiae) after treatments with UVC radiation, 8-MOP plus UVA and MMS. Mutation Research/Genetic Toxicology and Environmental Mutagenesis. 2006;606(1-2):27-38.
218. Saikia S, Bordoloi M, Sarmah R, Kolita B. Antiviral compound screening, peptide designing, and protein network construction of influenza a virus (strain a/Puerto Rico/8/1934 H1N1). Drug Development Research. 2019;80(1):106-24.
219. Townsend EA, Siviski ME, Zhang Y, Xu C, Hoonjan B, Emala CW. Effects of ginger and its constituents on airway smooth muscle relaxation and calcium regulation. American journal of respiratory cell and molecular biology. 2013;48(2):157-63.
220. Yu Y, Huang T, Yang B, Liu X, Duan G. Development of gas chromatography–mass spectrometry with microwave distillation and simultaneous solid-phase microextraction for rapid determination of volatile constituents in ginger. Journal of pharmaceutical and biomedical analysis. 2007;43(1):24-31.
221. Chainani-Wu N. Safety and anti-inflammatory activity of curcumin: a component of tumeric (Curcuma longa). The Journal of Alternative & Complementary Medicine. 2003;9(1):161-8.
222. Senanayake UM, Lee TH, Wills RB. Volatile constituents of cinnamon (Cinnamomum zeylanicum) oils. Journal of agricultural and food chemistry. 1978;26(4):822-4.
223. Chericoni S, Prieto JM, Iacopini P, Cioni P, Morelli I. In vitro activity of the essential oil of Cinnamomum zeylanicum and eugenol in peroxynitrite-induced oxidative processes. Journal of agricultural and food chemistry. 2005;53(12):4762-5.
224. Rabinkov A, Miron T, Konstantinovski L, Wilchek M, Mirelman D, Weiner L. The mode of action of allicin: trapping of radicals and interaction with thiol containing proteins. Biochimica et Biophysica Acta (BBA)-General Subjects. 1998;1379(2):233-44.
225. Butt MS, Sultan MT, Butt MS, Iqbal J. Garlic: nature's protection against physiological threats. Critical reviews in food science and nutrition. 2009;49(6):538-51.
226. Yamazaki Y, Iwasaki K, Mikami M, Yagihashi A. Distribution of eleven flavor precursors, S-alk (en) yl-L-cysteine derivatives, in seven Allium vegetables. Food science and technology research. 2010;17(1):55-62.
227. Sellappan S, Akoh CC. Flavonoids and antioxidant capacity of Georgia-grown Vidalia onions. Journal of agricultural and food chemistry. 2002;50(19):5338-42.
228. Burits M, Bucar F. Antioxidant activity of Nigella sativa essential oil. Phytotherapy research. 2000;14(5):323-8.
229. İşcan G, Kirimer N, Kürkcüoğlu Mn, Başer HC, Demirci F. Antimicrobial screening of Mentha piperita essential oils. Journal of agricultural and food chemistry. 2002;50(14):3943-6.
230. González F, Quintero J, Del Río R, Mahn A. Optimization of an extraction process to obtain a food-grade sulforaphane-rich extract from broccoli (Brassica oleracea var. italica). Molecules. 2021;26(13):4042.
231. Justesen U, Knuthsen P. Composition of flavonoids in fresh herbs and calculation of flavonoid intake by use of herbs in traditional Danish dishes. Food chemistry. 2001;73(2):245-50.
232. Meghwal M, Goswami T. Piper nigrum and piperine: an update. Phytotherapy Research. 2013;27(8):1121-30.
233. Li X-J, Kim K-W, Oh H, Liu X-Q, Kim Y-C. Chemical constituents and an antineuroinflammatory lignan, savinin from the roots of Acanthopanax henryi. Evidence-Based Complementary and Alternative Medicine. 2019;2019.
234. Al-Harrasi A, Rehman NU, Khan AL, Al-Broumi M, Al-Amri I, Hussain J, et al. Chemical, molecular and structural studies of Boswellia species: β-Boswellic Aldehyde and 3-epi-11β-Dihydroxy BA as precursors in biosynthesis of boswellic acids. PLoS One. 2018;13(6):e0198666.

235. Krasutsky PA. Birch bark research and development. Natural product reports. 2006;23(6):919-42.
236. Mbaze LMa, Lado JA, Wansi JD, Shiao TC, Chiozem DD, Mesaik MA, et al. Oxidative burst inhibitory and cytotoxic amides and lignans from the stem bark of Fagara heitzii (Rutaceae). Phytochemistry. 2009;70(11-12):1442-7.
237. Cho JY, Park J, Kim PS, YOO ES, BAIK KU, PARK MH. Savinin, a lignan from Pterocarpus santalinus inhibits tumor necrosis factor-α production and T cell proliferation. Biological and Pharmaceutical Bulletin. 2001;24(2):167-71.
238. Woo KW, Choi SU, Park JC, Lee KR. A new lignan glycoside from Juniperus rigida. Archives of Pharmacal Research. 2011;34:2043-9.
239. Lee S, Yoo HH, Piao XL, Kim JS, Kang SS, Shin KH. Anti-estrogenic activity of lignans from Acanthopanax chiisanensis root. Archives of pharmacal research. 2005;28:186-9.
240. Jung HJ, Jung HA, Min B-S, Choi JS. Anticholinesterase and β-site amyloid precursor protein cleaving enzyme 1 inhibitory compounds from the heartwood of Juniperus chinensis. Chemical and Pharmaceutical Bulletin. 2015;63(11):955-60.
241. Wen C-C, Kuo Y-H, Jan J-T, Liang P-H, Wang S-Y, Liu H-G, et al. Specific plant terpenoids and lignoids possess potent antiviral activities against severe acute respiratory syndrome coronavirus. Journal of medicinal chemistry. 2007;50(17):4087-95.
242. Adesanwo JK, Makinde OO, Obafemi CA. Phytochemical analysis and antioxidant activity of methanol extract and betulinic acid isolated from the roots of Tetracera potatoria. Journal of Pharmacy Research. 2013;6(9):903-7.
243. Tzakos AG, Kontogianni VG, Tsoumani M, Kyriakou E, Hwa J, Rodrigues FA, Tselepis AD. Exploration of the antiplatelet activity profile of betulinic acid on human platelets. Journal of agricultural and food chemistry. 2012;60(28):6977-83.
244. Mukherjee PK, Saha K, Das J, Pal M, Saha B. Studies on the anti-inflammatory activity of rhizomes of Nelumbo nucifera. Planta medica. 1997;63(04):367-9.
245. Shin S-J, Park C-E, Baek N-I, Chung IS, Park C-H. Betulinic and oleanolic acids isolated from Forsythia suspensa Vahl inhibit urease activity of Helicobacter pylori. Biotechnology and Bioprocess Engineering. 2009;14:140-5.
246. Pisha E, Chai H, Lee I-S, Chagwedera TE, Farnsworth NR, Cordell GA, et al. Discovery of betulinic acid as a selective inhibitor of human melanoma that functions by induction of apoptosis. Nature medicine. 1995;1(10):1046-51.
247. Krogh R, Kroth R, Berti C, Madeira A, Souza M, Cechinel-Filho V, et al. Isolation and identification of compounds with antinociceptive action from Ipomoea pes-caprae (L.) R. Br. Die pharmazie. 1999;54(6):464-6.
248. Bringmann G, Saeb W, Assi LA, Francois G, Narayanan AS, Peters K, Peters E-M. Betulinic acid: isolation from Triphyophyllum peltatum and Ancistrocladus heyneanus, antimalarial activity, and crystal structure of the benzyl ester. Planta medica. 1997;63(03):255-7.
249. Zhang Z, ElSohly HN, Jacob MR, Pasco DS, Walker LA, Clark AM. Natural products inhibiting Candida albicans secreted aspartic proteases from Tovomita krukovii. Planta Medica. 2002;68(01):49-54.
250. Kim J, Lee YS, Kim CS, Kim JS. Betulinic acid has an inhibitory effect on pancreatic lipase and induces adipocyte lipolysis. Phytotherapy Research. 2012;26(7):1103-6.
251. Yi J, Xia W, Wu J, Yuan L, Wu J, Tu D, et al. Betulinic acid prevents alcohol-induced liver damage by improving the antioxidant system in mice. Journal of veterinary science. 2014;15(1):141-8.

252. Fu J-Y, Qian L-B, Zhu L-G, Liang H-T, Tan Y-N, Lu H-T, et al. Betulinic acid ameliorates endothelium-dependent relaxation in L-NAME-induced hypertensive rats by reducing oxidative stress. European journal of pharmaceutical sciences. 2011;44(3):385-91.
253. Ríos JL, Máñez S. New pharmacological opportunities for betulinic acid. Planta medica. 2018;84(01):8-19.
254. Machado DG, Cunha MP, Neis VB, Balen GO, Colla A, Bettio LE, et al. Antidepressant-like effects of fractions, essential oil, carnosol and betulinic acid isolated from Rosmarinus officinalis L. Food Chemistry. 2013;136(2):999-1005.
255. Yi J-e, Obminska-Mrukowicz B, Yuan L-y, Yuan H. Immunomodulatory effects of betulinic acid from the bark of white birch on mice. Journal of veterinary science. 2010;11(4):305-13.
256. Fujioka T, Kashiwada Y, Kilkuskie RE, Cosentino LM, Ballas LM, Jiang JB, et al. Anti-AIDS agents, 11. Betulinic acid and platanic acid as anti-HIV principles from Syzigium claviflorum, and the anti-HIV activity of structurally related triterpenoids. Journal of natural products. 1994;57(2):243-7.
257. Reutrakul V, Chanakul W, Pohmakotr M, Jaipetch T, Yoosook C, Kasisit J, et al. Anti-HIV-1 constituents from leaves and twigs of Cratoxylum arborescens. Planta medica. 2006;72(15):1433-5.
258. Ryu SY, Lee C-K, Lee CO, Kim HS, Zee OP. Antiviral triterpenes from Prunella vulgaris. Archives of Pharmacal Research. 1992;15:242-5.
259. Pavlova N, Savinova O, Nikolaeva S, Boreko E, Flekhter O. Antiviral activity of betulin, betulinic and betulonic acids against some enveloped and non-enveloped viruses. Fitoterapia. 2003;74(5):489-92.
260. Hong E-H, Song JH, Kang KB, Sung SH, Ko H-J, Yang H. Anti-influenza activity of betulinic acid from Zizyphus jujuba on influenza A/PR/8 virus. Biomolecules & therapeutics. 2015;23(4):345.
261. Yao D, Li H, Gou Y, Zhang H, Vlessidis AG, Zhou H, et al. Betulinic acid-mediated inhibitory effect on hepatitis B virus by suppression of manganese superoxide dismutase expression. The FEBS journal. 2009;276(9):2599-614.
262. Lin CK, Tseng CK, Chen KH, Wu SH, Liaw CC, Lee JC. Betulinic acid exerts anti-hepatitis C virus activity via the suppression of NF-κ B-and MAPK-ERK 1/2-mediated COX-2 expression. British journal of pharmacology. 2015;172(18):4481-92.
263. Sharma O. Antioxidant activity of curcumin and related compounds. Biochemical pharmacology. 1976;25(15):1811-2.
264. Kim D-C, Ku S-K, Bae J-S. Anticoagulant activities of curcumin and its derivative. BMB reports. 2012;45(4):221-6.
265. Srimal R, Dhawan B. Pharmacology of diferuloyl methane (curcumin), a non-steroidal anti-inflammatory agent. Journal of pharmacy and pharmacology. 1973;25(6):447-52.
266. Rai D, Singh JK, Roy N, Panda D. Curcumin inhibits FtsZ assembly: an attractive mechanism for its antibacterial activity. Biochemical Journal. 2008;410(1):147-55.
267. Aggarwal BB, Kumar A, Bharti AC. Anticancer potential of curcumin: preclinical and clinical studies. Anticancer research. 2003;23(1/A):363-98.
268. Nose M, Koide T, Ogihara Y, YABU Y, OHTA N. Trypanocidal effects of curcumin in vitro. Biological and Pharmaceutical Bulletin. 1998;21(6):643-5.
269. Martins C, Da Silva D, Neres A, Magalhaes T, Watanabe G, Modolo L, et al. Curcumin as a promising antifungal of clinical interest. Journal of Antimicrobial Chemotherapy. 2009;63(2):337-9.
270. Kang H-C, Nan J-X, Park P-H, Kim J-Y, Lee SH, Woo SW, et al. Curcumin inhibits collagen synthesis and hepatic stellate cell activation in-vivo and in-vitro. Journal of pharmacy and pharmacology. 2002;54(1):119-26.

271. Jana S, Paul S, Swarnakar S. Curcumin as anti-endometriotic agent: Implication of MMP-3 and intrinsic apoptotic pathway. Biochemical pharmacology. 2012;83(6):797-804.
272. Mahesh T, Balasubashini MMS, Menon VP. Photo-irradiated curcumin supplementation in streptozotocin-induced diabetic rats: effect on lipid peroxidation. Therapies. 2004;59(6):639-44.
273. Alappat L, Awad AB. Curcumin and obesity: evidence and mechanisms. Nutrition reviews. 2010;68(12):729-38.
274. Xu Y, Ku B-S, Yao H-Y, Lin Y-H, Ma X, Zhang Y-H, Li X-J. The effects of curcumin on depressive-like behaviors in mice. European journal of pharmacology. 2005;518(1):40-6.
275. Panahi Y, Rahimnia AR, Sharafi M, Alishiri G, Saburi A, Sahebkar A. Curcuminoid treatment for knee osteoarthritis: A randomized double-blind placebo-controlled trial. Phytotherapy research. 2014;28(11):1625-31.
276. Inano H, Onoda M. Radioprotective action of curcumin extracted from Curcuma longa LINN: inhibitory effect on formation of urinary 8-hydroxy-2′-deoxyguanosine, tumorigenesis, but not mortality, induced by γ-ray irradiation. International Journal of Radiation Oncology* Biology* Physics. 2002;53(3):735-43.
277. Wang R, Li Y-H, Xu Y, Li Y-B, Wu H-L, Guo H, et al. Curcumin produces neuroprotective effects via activating brain-derived neurotrophic factor/TrkB-dependent MAPK and PI-3K cascades in rodent cortical neurons. Progress in Neuro-Psychopharmacology and Biological Psychiatry. 2010;34(1):147-53.
278. Gao X, Kuo J, Jiang H, Deeb D, Liu Y, Divine G, et al. Immunomodulatory activity of curcumin: suppression of lymphocyte proliferation, development of cell-mediated cytotoxicity, and cytokine production in vitro. Biochemical pharmacology. 2004;68(1):51-61.
279. Peschel D, Koerting R, Nass N. Curcumin induces changes in expression of genes involved in cholesterol homeostasis. The Journal of nutritional biochemistry. 2007;18(2):113-9.
280. Sidhu GS, Singh AK, Thaloor D, Banaudha KK, Patnaik GK, Srimal RC, Maheshwari RK. Enhancement of wound healing by curcumin in animals. Wound Repair and Regeneration. 1998;6(2):167-77.
281. Lim GP, Chu T, Yang F, Beech W, Frautschy SA, Cole GM. The curry spice curcumin reduces oxidative damage and amyloid pathology in an Alzheimer transgenic mouse. Journal of Neuroscience. 2001;21(21):8370-7.
282. Li Y, Wang J, Liu Y, Luo X, Lei W, Xie L. Antiviral and virucidal effects of curcumin on transmissible gastroenteritis virus in vitro. Journal of General Virology. 2020;101(10):1079-84.
283. Obata K, Kojima T, Masaki T, Okabayashi T, Yokota S, Hirakawa S, et al. Curcumin prevents replication of respiratory syncytial virus and the epithelial responses to it in human nasal epithelial cells. PLoS One. 2013;8(9):e70225.
284. Du T, Shi Y, Xiao S, Li N, Zhao Q, Zhang A, et al. Curcumin is a promising inhibitor of genotype 2 porcine reproductive and respiratory syndrome virus infection. BMC veterinary research. 2017;13(1):1-9.
285. Zandi K, Ramedani E, Mohammadi K, Tajbakhsh S, Deilami I, Rastian Z, et al. Evaluation of antiviral activities of curcumin derivatives against HSV-1 in Vero cell line. Natural product communications. 2010;5(12):1934578X1000501220.
286. Ferreira VH, Nazli A, Dizzell SE, Mueller K, Kaushic C. The anti-inflammatory activity of curcumin protects the genital mucosal epithelial barrier from disruption and blocks replication of HIV-1 and HSV-2. PloS one. 2015;10(4):e0124903.
287. Li H, Zhong C, Wang Q, Chen W, Yuan Y. Curcumin is an APE1 redox inhibitor and exhibits an antiviral activity against KSHV replication and pathogenesis. Antiviral research. 2019;167:98-103.

288. Hergenhahn M, Soto U, Weninger A, Polack A, Hsu CH, Cheng AL, Rösl F. The chemopreventive compound curcumin is an efficient inhibitor of Epstein-Barr virus BZLF1 transcription in Raji DR-LUC cells. Molecular Carcinogenesis: Published in cooperation with the University of Texas MD Anderson Cancer Center. 2002;33(3):137-45.
289. Lv Y, Gong L, Wang Z, Han F, Liu H, Lu X, Liu L. Curcumin inhibits human cytomegalovirus by downregulating heat shock protein 90. Molecular Medicine Reports. 2015;12(3):4789-93.
290. Mazumder A, Raghavan K, Weinstein J, Kohn KW, Pommier Y. Inhibition of human immunodeficiency virus type-1 integrase by curcumin. Biochemical pharmacology. 1995;49(8):1165-70.
291. Zhang HS, Ruan Z, Sang WW. HDAC1/NFκB pathway is involved in curcumin inhibiting of Tat-mediated long terminal repeat transactivation. Journal of cellular physiology. 2011;226(12):3385-91.
292. Ali A, Banerjea AC. Curcumin inhibits HIV-1 by promoting Tat protein degradation. Scientific reports. 2016;6(1):27539.
293. Vajragupta O, Boonchoong P, Morris GM, Olson AJ. Active site binding modes of curcumin in HIV-1 protease and integrase. Bioorganic & medicinal chemistry letters. 2005;15(14):3364-8.
294. Barthelemy S, Vergnes L, Moynier M, Guyot D, Labidalle S, Bahraoui E. Curcumin and curcumin derivatives inhibit Tat-mediated transactivation of type 1 human immunodeficiency virus long terminal repeat. Research in virology. 1998;149(1):43-52.
295. Mounce BC, Cesaro T, Carrau L, Vallet T, Vignuzzi M. Curcumin inhibits Zika and chikungunya virus infection by inhibiting cell binding. Antiviral research. 2017;142:148-57.
296. Si X, Wang Y, Wong J, Zhang J, McManus BM, Luo H. Dysregulation of the ubiquitin-proteasome system by curcumin suppresses coxsackievirus B3 replication. Journal of virology. 2007;81(7):3142-50.
297. Qin Y, Lin L, Chen Y, Wu S, Si X, Wu H, et al. Curcumin inhibits the replication of enterovirus 71 in vitro. Acta Pharmaceutica Sinica B. 2014;4(4):284-94.
298. Rechtman MM, Har-Noy O, Bar-Yishay I, Fishman S, Adamovich Y, Shaul Y, et al. Curcumin inhibits hepatitis B virus via down-regulation of the metabolic coactivator PGC-1α. FEBS letters. 2010;584(11):2485-90.
299. Wei Z-Q, Zhang Y-H, Ke C-Z, Chen H-X, Ren P, He Y-L, et al. Curcumin inhibits hepatitis B virus infection by down-regulating cccDNA-bound histone acetylation. World journal of gastroenterology. 2017;23(34):6252.
300. Kim K, Kim KH, Kim HY, Cho HK, Sakamoto N, Cheong J. Curcumin inhibits hepatitis C virus replication via suppressing the Akt-SREBP-1 pathway. FEBS letters. 2010;584(4):707-12.
301. Chen M-H, Lee M-Y, Chuang J-J, Li Y-Z, Ning S-T, Chen J-C, Liu Y-W. Curcumin inhibits HCV replication by induction of heme oxygenase-1 and suppression of AKT. International journal of molecular medicine. 2012;30(5):1021-8.
302. Chen D-Y, Shien J-H, Tiley L, Chiou S-S, Wang S-Y, Chang T-J, et al. Curcumin inhibits influenza virus infection and haemagglutination activity. Food Chemistry. 2010;119(4):1346-51.
303. Dai J, Gu L, Su Y, Wang Q, Zhao Y, Chen X, et al. Inhibition of curcumin on influenza A virus infection and influenzal pneumonia via oxidative stress, TLR2/4, p38/JNK MAPK and NF-κB pathways. International immunopharmacology. 2018;54:177-87.
304. Yang M, Lee G, Si J, Lee S-J, You HJ, Ko G. Curcumin shows antiviral properties against norovirus. Molecules. 2016;21(10):1401.
305. Dutta K, Ghosh D, Basu A. Curcumin protects neuronal cells from Japanese encephalitis virus-mediated cell death and also inhibits infective viral particle formation by dysregulation of ubiquitin–proteasome system. Journal of Neuroimmune Pharmacology. 2009;4:328-37.

306. Padilla-s L, Rodríguez A, Gonzales MM, Gallego-g JC, Castaño-o JC. Inhibitory effects of curcumin on dengue virus type 2-infected cells in vitro. Archives of virology. 2014;159:573-9.
307. Narayanan A, Kehn-Hall K, Senina S, Lundberg L, Van Duyne R, Guendel I, et al. Curcumin inhibits Rift Valley fever virus replication in human cells. Journal of Biological Chemistry. 2012;287(40):33198-214.
308. Sordillo PP, Helson L. Curcumin suppression of cytokine release and cytokine storm. A potential therapy for patients with Ebola and other severe viral infections. in vivo. 2015;29(1):1-4.
309. Jeong E-H, Vaidya B, Cho S-Y, Park M-A, Kaewintajuk K, Kim SR, et al. Identification of regulators of the early stage of viral hemorrhagic septicemia virus infection during curcumin treatment. Fish & Shellfish Immunology. 2015;45(1):184-93.
310. Lockbaum GJ, Reyes AC, Lee JM, Tilvawala R, Nalivaika EA, Ali A, et al. Crystal structure of SARS-CoV-2 main protease in complex with the non-covalent inhibitor ML188. Viruses. 2021;13(2):174.
311. Yang KS, Leeuwon SZ, Xu S, Liu WR. Evolutionary and structural insights about potential SARS-CoV-2 evasion of nirmatrelvir. Journal of Medicinal Chemistry. 2022;65(13):8686-98.
